# Multiplex CRISPR/Cas9-mediated knockout of the *phytoene desaturase* gene in *Coffea canephora*

**DOI:** 10.1038/s41598-022-21566-w

**Published:** 2022-10-14

**Authors:** Tatiane Casarin, Natália Chagas Freitas, Renan Terassi Pinto, Jean‑Christophe Breitler, Leonardo Augusto Zebral Rodrigues, Pierre Marraccini, Hervé Etienne, Leandro Eugenio Cardamone Diniz, Alan Carvalho Andrade, Luciano Vilela Paiva

**Affiliations:** 1 LCBM, UFLA, Lavras, 3037 Brazil; 2 CIRAD, UMR DIADE, 34398 Montpellier, France; 3 CIRAD, IRD, UMR DIADE, University of Montpellier, 34398 Montpellier, France; 4 Embrapa Soybean, Londrina, 86085-981 Brazil; 5 Embrapa Coffee - Inovacafé, Lavras, 3037 Brazil

**Keywords:** Plant biotechnology, Molecular engineering in plants

## Abstract

*Coffea canephora* (2n = 2x = 22 chromosomes) is a species with extensive genetic diversity and desirable agronomic traits for coffee breeding programs. However, obtaining a new coffee cultivar through conventional breeding techniques may require more than 30 years of crossing cycles and selection, which hampers the effort of keeping up with market demands and rapidly proposing more resilient to climate change varieties. Although, the application of modern biotechnology tools such as precision genetic engineering technologies may enable a faster cultivar development process. Therefore, we aimed to validate the CRISPR/Cas9 system to generate mutations on a selected genotype of *C. canephora*, the clone 14. Embryogenic calli and a multiplex binary vector containing two sgRNAs targeting different exons of the *CcPDS* gene were used. The sgRNAs were under the *C. canephora* U6 promoter regulation. The target gene encodes phytoene desaturase, an enzyme essential for photosynthesis involved in *β*-carotene biosynthesis. Somatic seedlings and embryos with albino, variegated and green phenotypes regenerated after *Agrobacterium tumefaciens*-mediated genetic transformation were analyzed by verifying the insertion of the *Cas9* gene and later by sequencing the sgRNAs target regions in the genome of Robusta modified seedlings. Among them, 77% had the expected mutations, and of which, 50% of them had at least one target with a homozygous mutation. The genotype, temperature of co-cultivation with the bacteria, and light intensity used for subsequent embryo regeneration appeared to strongly influence the successful regeneration of plants with a mutated *CcPDS* gene in the *Coffea* genus.

## Introduction

Coffee is among the most profitable product in the global economy with a significant social role, as the great majority of growers worldwide are small farmers. The coffee business and industry are worth more than 90 billion dollars annually and is the income source for more than 125 million people in many countries in Asia, Africa, and Latin America^1,2^. Coffee has a rich chemical and sensory constitution. Therefore, it can be consumed in different forms and preparations: hot and cold drinks, as an ingredient in the preparation of food products, and as a constituent in cosmetics and medicines, owing to its nutraceutical properties. Although the *Coffea* genus consists of more than 125 species, only two of them are commercially cultivated, *Coffea arabica* and *Coffea canephora*^2,3^.

*C. canephora* (also known as Robusta) is cultivated in low to medium altitude intertropical regions of Africa, America, and Asia and is a self-incompatible diploid species (2n = 2x = 22 chromosomes) with high genetic diversity^4–6^. Since the accessions from this species present resistance to most pests and diseases and greater tolerance to high temperatures and water deficit than most *C. arabica* genotypes, it has been widely used in coffee breeding programs^7^. One such example, the clone 14 (Instituto Capixaba de Pesquisa, Assistência Técnica e Extensão Rural—INCAPER), has been extensively studied for its resistance to drought^8–13^ and multi-resistance to nematodes^14^.

Due to frequent changes in consumer preferences and increasing climatic variations^15^, it is essential to develop improved genotypes from available breeding programs at a fast pace and to propose innovative selection methods that are faster and more accurate. Therefore, biotechnological tools can help accelerate the acquisition of elite varieties, as it is possible to skip crossing cycles by inserting new characteristics into a chosen genotype^1^. A recent technology with great potential for application in plant breeding is the CRISPR/Cas genomic editing system, which has high robustness, specificity, and programmability, as well as constant improvements and adaptations for novel applications^16^. This technique is based on an adaptive immune system from bacteria and Archaea, where small RNA fragments (sgRNAs) guide the Cas9 endonuclease to induce double-strand breaks (DSBs) in the targeted DNA. Depending on errors in the DNA repair system of the cell, indels can be generated^17^.

CRISPR technology has evolved rapidly and successfully applied to several model plants and crops, but some optimization is still needed for other plant species, including trees^18^. The first report demonstrating the system's efficiency in woody plants involved silencing the *phytoene desaturase* (*PDS*) gene in *Populus tomentosa.* This gene is part of the carotenoid biosynthetic pathway and is commonly used to validate gene silencing techniques due to the albino-like phenotype expressed in the mutants^19^*.* In coffee, an extensive survey of all possible target regions for CRISPR/Cas9 in the genome of *C. canephora* has already been carried out through the development of a web tool. Although homozygous and heterozygous mutations were obtained from experiments to validate the system, the mutants did not present the expected albino phenotype when targeting the *CcPDS* (Cc04_g00540) gene^20^ (BREITLER et al., 2018).

In this study, we validated the CRISPR/Cas9 genomic editing system in coffee using vectors for inducing multiple mutations and obtained mutant plants with the expected albino phenotype in *C. canephora* clone 14, a genotype with important agronomic traits.

## Results

### Somatic embryo regeneration after transformation and altered albino phenotypes

**Figure 1:**
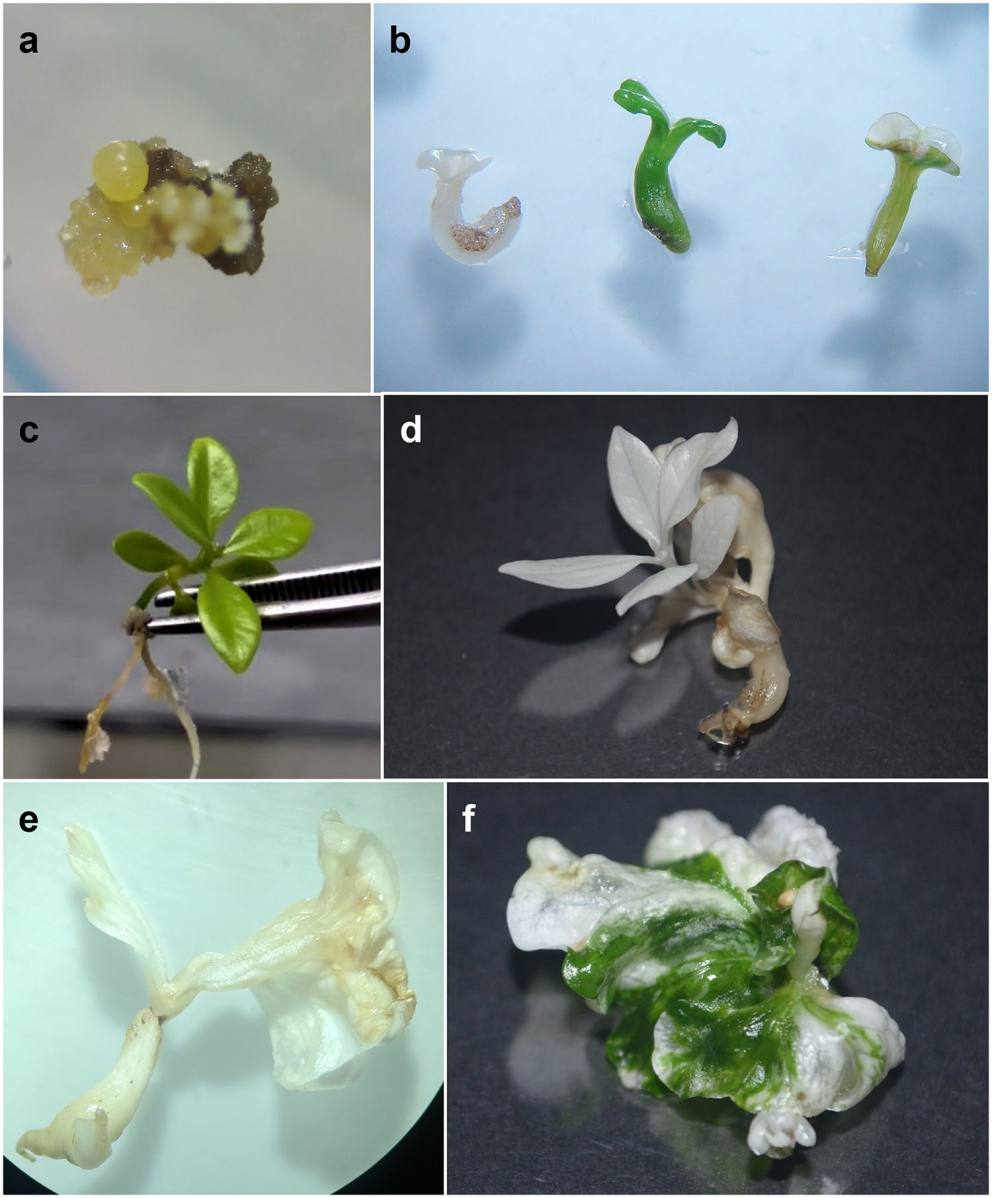
Coffee plant material regenerated after CRISPR/Cas9-mediated knockout of the *phytoene desaturase* gene using *Agrobacterium tumefaciens*-mediated transformation. Images representing the development of embryogenic calli, transformed somatic embryos and somatic seedlings and the different embryo phenotypes obtained. (**A)** Hygromycin-resistant yellowish embryogenic calli growing from oxidized primary calli formed on the explant. (**B**) Albino, green, and variegated cotyledonary transformed embryos. (**C**) WT somatic seedling. (**D**) One of the albino somatic seedlings obtained after CRISPR/Cas9 transformation; (**E**,**F**) Aspect of transformed embryos with abnormal development of albino (**E**) and variegated (**F**) cotyledons.

Due to the action of the selective agent (hygromycin), immediately after transformation, the embryogenic calli showed intense oxidation. After four months, some hygromycin-resistant yellowish calli were observed, as expected according to the protocol adopted by Ribas et al.^21^. Four months later, the first globular embryos were observed from these yellow calli (Fig. 1A). Embryos with three different phenotypes were obtained at the cotyledonary developmental stage: completely albino, completely green, and partially albino (variegated) (Fig. 1B). Approximately one year after co-cultivation with the Agrobacteria, cotyledonary embryos displayed significant growth (Fig. 1C).

Some albino and variegated embryos developed into seedlings (Fig. 1D) that produced new pairs of leaves. Under a very low light intensity of approximately 2 µmol m^−2^ s^−1^, these seedlings could be maintained in vitro at this developmental stage (2–4 pairs of albino leaves) for approximately 12 months until they showed intense photooxidation and necrosis due to the absence of chlorophyll. At this point, healthy tissues were collected and frozen in liquid nitrogen for future molecular analysis. Other albino and variegated seedlings (Fig. 1E,F, respectively) showed abnormal cotyledon development and maintained this phenotype until they also began to oxidize and necrotize.

### Confirmation of T-DNA insertion and mutation sequencing

The confirmation of genetic transformation was performed based on detecting the insertion of T-DNA in seven seedlings with the albino phenotype, four seedlings with the variegated phenotype, and three with the normal green phenotype, all of which had resistance to hygromycin (6 months of cultivation on 100 mg L^−1^ of hygromycin). All transformed seedlings showed amplification of the approximately 500 bp band corresponding to the expected amplicon size for the *Cas9* gene in the cassette (Supplementary Figs. S1 and S2 online). From albino seedlings, six were used in the subsequent analyses due to the availability of biological material from one of the samples. For the two wild-type (WT) seedlings analyzed, as expected, the 500 bp amplicon was absent. Afterward, the transformed plants were evaluated for the occurrence of mutations by cloning and sequencing the target regions of the *CcPDS* gene.

**Figure 2:**
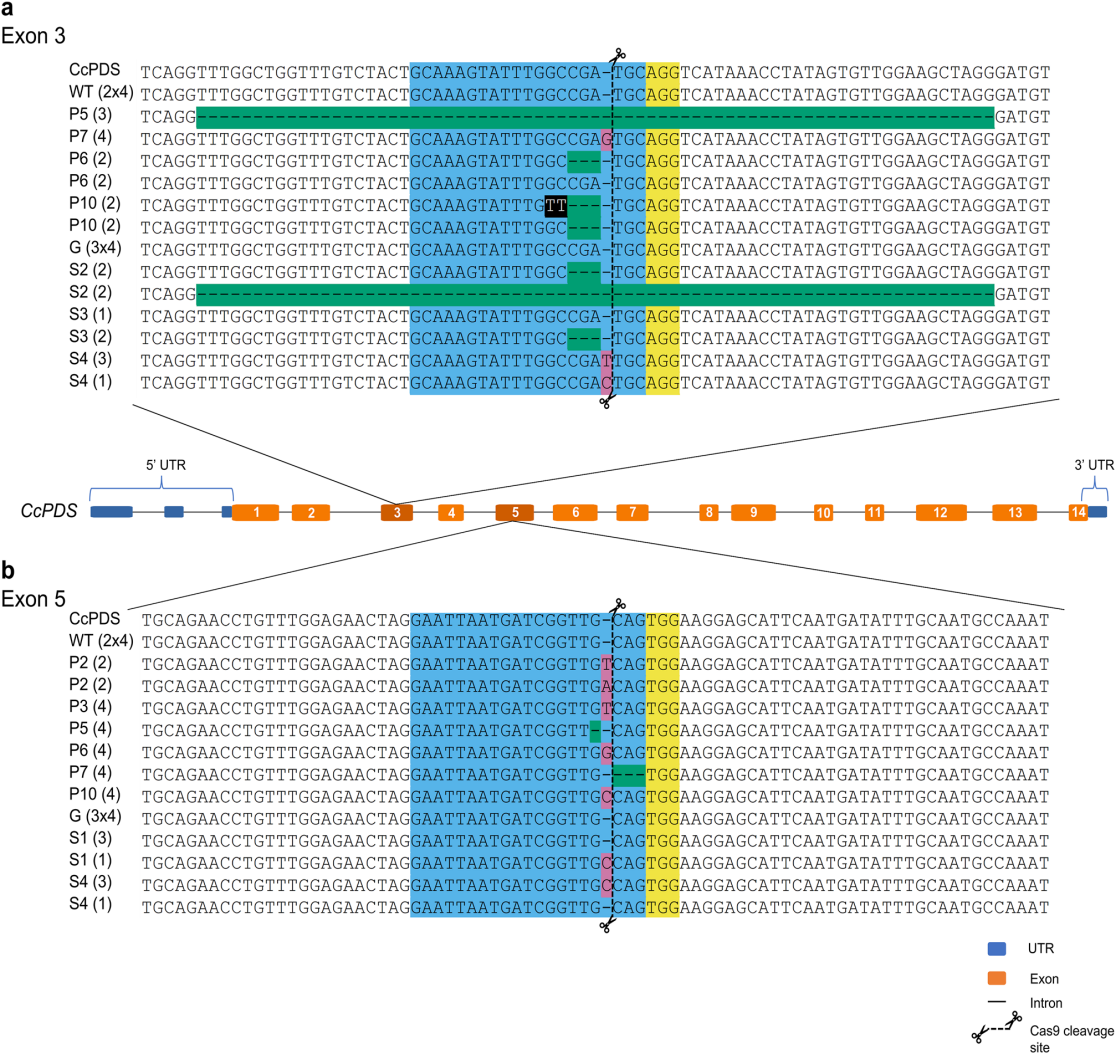
Schematic representation of *CcPDS* gene structure. The alignment results for the reference gene (Cc04_g00540—chr4:412113..419155, Coffee Genome Hub, http://coffee-genome.org/), control (WT), and transformed seedlings *CcPDS* sequences. *CcPDS*: reference gene; a: comparisons of sequencing results for target 1, at the exon 3; b: comparisons of sequencing results for target 2, at exon 5. Abbreviations refer to the following samples: CcPDS: reference gene sequence from Coffee Genome Hub; WT: control, untransformed; P: albino seedlings (transformed); G: green seedlings (transformed); and S: variegated seedlings (transformed). At the sequence results, each color represents the following: Blue: sgRNA target sequence; Yellow: PAM sequence; Black: base substitutions; Pink: base insertions; Green: base deletions; Black scissor with vertical dashed lines: Cas9 cleavage site. Each number in parentheses represents the number of sequenced colonies for a given sample in which the same sequencing result was found. In the case of WT and green seedlings, the number of sequenced seedlings is also represented.

**Figure 3:**
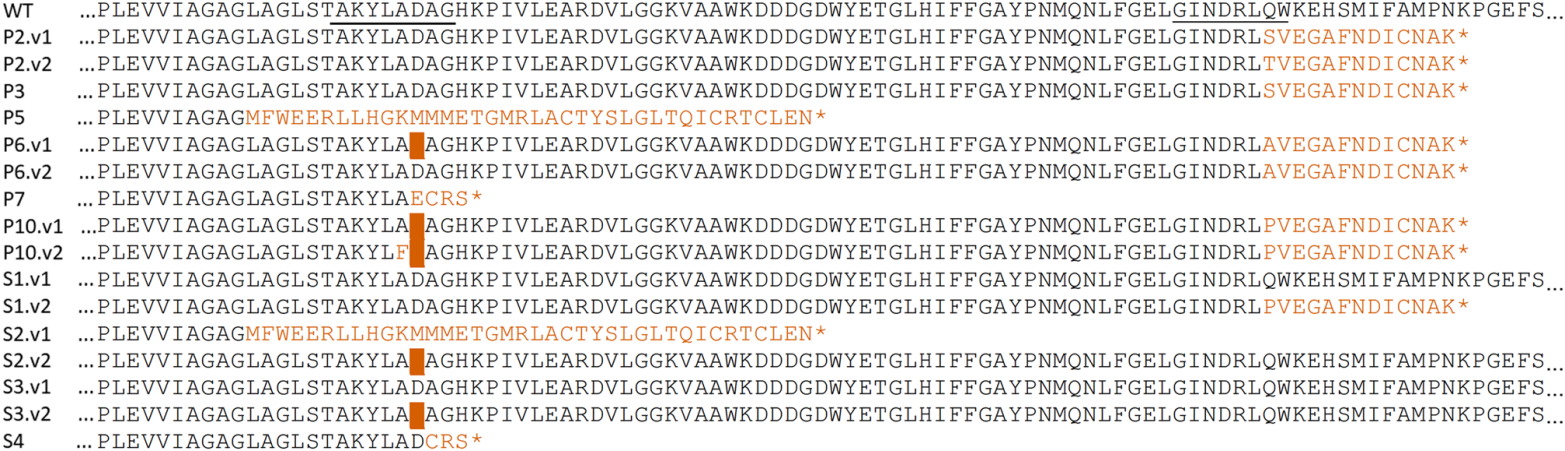
Schematic representation of the amino acid sequences translated from the edited *CcPDS* gene sequences after coffee genetic transformation using the CRISPR/Cas9 methodology. Altered amino acids are represented in orange, the bar represents an amino acid deletion, and the asterisk represents a stop codon. The underlined regions in the control sequence (WT) represent the amino acids corresponding to the regions of the two sgRNAs.

The sequence alignment results for the reference (*CcPDS*), control untransformed somatic seedlings (WT), and transformed seedlings (P for albino and S for variegated) are shown in Fig. 2. For target 1 (Fig. 2A), mutations were observed in four of the six albino somatic seedlings. Two of them had homozygous mutations (P5 with a 70 nt deletion and P7 with a one nt addition), one had a heterozygous mutation (P6 with a three nt deletion in only one allele), and one had a biallelic mutation (P10 with a deletion of 3 nt in both alleles and base swaps in two positions on only one allele). However, after verifying the effect of these mutations on protein translation (Fig. 3), the mutations generated with the PDS3 sgRNA were correlated with the albino phenotype only for the P5 and P7 seedlings, probably due to the generation of an early stop codon immediately after the mutated positions. As shown in Fig. 3, the mutations found in the P6 (deletion of one amino acid) and P10 (one deleted and one replaced amino acid) seedlings only resulted in a stop codon after the mutation in target 2. None of the three transformed seedlings with a green phenotype presented mutations in any target (G). Among the somatic seedlings showing the variegated phenotype, two presented biallelic mutations (S2, with deletions of 3 and 70 nt in each allele; S4, with an insertion of 1 different base in each allele), and one presented a heterozygous mutation (S3, with a deletion of 3 nt in only one allele).

For target 2 (Fig. 2B), six transformed somatic seedlings (P2, P3, P5, P6, P7, P10) with an albino phenotype showed genomic mutations at the targeted sequence. Five mutations were homozygous, and one was biallelic; additionally, four mutations were insertions of a single nucleotide (P2, P3, P6, and P10), one was the deletion of a single nucleotide (P5), and one was a three nt deletion (P7). Thus, the PDS5 sgRNA exhibited greater effectiveness in inducing mutations in both alleles and generating the albino phenotype since, in all these cases, the observed mutations generated early stop codons (Fig. 3), resulting in a truncated protein and loss of function. Furthermore, the transformed somatic seedlings with a green phenotype did not have mutations in this target. Finally, for transformed somatic seedlings with variegated phenotype, two showed heterozygous mutations, with the insertion of one nucleotide in only one allele in both cases (S1 and S4).

Transformed somatic seedlings P2 and P3 had mutations only in target 2 but presented a complete albino phenotype. However, transformed seedlings S2 and S4 presented variegated phenotypes with biallelic mutations for target 1 (S4 also with heterozygous mutation in target 2). In the case of transformed seedling S2, the deletion of 3 nt from one of the alleles resulted in the deletion of one amino acid but did not generate an early stop codon; thus, the variegated phenotype may be a result of the partial functionality of the PDS protein generated.

The mutation rates are summarized in Table 1. For target 1 (exon 3), 53.9% of the analyzed seedling presented mutations, with most mutations observed being biallelic. For target 2 (exon 5), 61.5% of the seedlings presented mutations, most of which were homozygous. 77% of seedlings had some mutation in at least one of the target regions. For all seedlings analyzed, it was possible to amplify the regions corresponding to the sgRNAs individually. Therefore, large mutations due to the combination of two sgRNAs on the target gene were not identified.

**Table 1:** Analysis of the rate and type of mutations on the *CcPDS* gene obtained from the transformation of coffee seedlings with the pCOFEDIT-2 vector.

Target	No analyzed seedlings	No mutant seedlings	% Mutant seedlings	Mutations		
				Homozygosity	Heterozygosity	Biallelic
Exon 3	13	7	53.9	2	2	3
Exon 5	13	8	61.5	5	2	2
Total	13	10	76.9			

## Discussion

Our results demonstrate the effectiveness of using this system in coffee plants for inducing mutations and obtaining the expected albino phenotype (Figs. 1, 2). In a previous study aimed at validating gene editing in coffee, it was possible to obtain mutants for exon 14 of the *PDS* gene in *C. canephora*; however, no complete albino phenotype could be observed^20^. The previous hypothesis was that coffee somatic embryos have a high photosynthetic rate, and in the absence of photosynthetic pigments, these embryos would not be able to complete development.

In the above-cited study, mutants for targets in exons 3 and 5 were not obtained, as the authors only edited exon 14. Mutations in exons that are further downstream in the gene may be related to the milder phenotypic effect observed since mutations at the very beginning of the gene, such as those generating early stop codons, tend to cause drastic changes in protein translation.

Another difference to highlight between the two studies is the genotype used to obtain embryogenic calli. In the present study, the *C. canephora* clone 14 used may be less affected by the absence of photosynthetic pigments. However, more importantly, in the present study, the light intensity was reduced from approximately 20 µmol m^−2^ s^−1^ to 2 µmol m^−2^ s^−1^ for embryogenic regeneration callus and subsequent somatic embryo and plant development steps. This strategy may have minimized the harmful effects of the absence of chlorophyll and alleviated the photooxidation due to the absence of these pigments during the regeneration of mutants with more severe edits for protein functionality.

Another possibility that may have influenced the efficiency of obtaining albino embryos was the explant infection temperature applied. In the present study, the co-cultivation temperature with *Agrobacterium tumefaciens* was 28 °C, while in the study of Breitler et al.^20^, it was only 20 °C. There are reports of greater efficiency in the transformation of embryogenic calli of *C. arabica* when using an *A. tumafaciens* and *A. rhizogenes* co-cultivation temperature of 20 °C ^21,22^. However, these conditions may not be the most suitable for transforming *C. canephora* species using the EHA105 strain. These factors that directly affect the transformation efficiency can directly interfere with the number of mutant events and, consequently, with the diversity of genotypes and phenotypes of the transformed coffee plants regenerated at the end of the process. It is also worth mentioning that the temperature difference could have increased the editing efficiency since Cas9 nuclease activity is affected by temperature. Heat treatments^23,24^ and temporary increases in temperature during incubation^25^ are consistently effective at increasing the number of somatic mutations using different CRISPR systems. This gain in efficiency is related to the higher temperatures being closer to the optimal growth temperature of *S. pyogenes* (40 °C)^24^.

Somatic embryos and somatic seedlings of *C. canephora* showing mutations in the last exon of the *CcPDS* gene (exon 14) under the CRISPR/Cas9 system did not show a fully albino phenotype but did show a series of morphological changes, such as a reduction in size; changes in positioning, organization, and pigmentation (yellowish, chlorotic or anthocyanin); and changes in the number of leaves. These embryos also presented rooting abnormalities^20^. The first report of stable transformation and CRISPR/Cas9-induced mutagenesis in woody plants was mentioned in *Populus tomentosa*, in which the mutants displayed diverse phenotypes, including the fully albino phenotype^19^. This phenomenon was also observed in apple trees, where transformed plants with a fully albino phenotype had limited growth compared to normal plants^26^. In this study, besides albinism, we also observed seedlings with abnormal development of cotyledons (Fig. 1), rooting abnormalities, and limited growth.

The evidence of phenotypic alteration induced by gene editing via CRISPR/Cas9 presented in this work reinforces expectations related to the ability to obtain desirable characteristics via molecular design for the rapid development of new improved varieties^27^, which is particularly interesting for agriculturally important species such as *C. canephora*. This technology can modify the only targeted agronomic trait in a single step while keeping the other characteristics of the variety known and appreciated by farmers and the entire industry. In a conservative agricultural world, this is a crucial point that guarantees the rapid adoption of the mutated variety. The cultivation record of *C. canephora* began approximately 100 years ago^28^ and suggests a very recent domestication process, although the use of this species for coffee production has the potential to help with sustainable agriculture goals. In addition, breeding programs can exploit the intraspecific variability arising from variation in wild species.

Furthermore, *C. canephora* is diploid (unlike *C. arabica*, which is allotetraploid), a characteristic that also allows the exploration of diversity within the *Coffea* genus as this genus is composed of other diploid species such as the recently identified *Coffea humboldtiana*, with interesting characteristics which can be incorporated into coffee breeding and agriculture. For instance, *C. humboldtiana* produces naturally decaffeinated beans, probably due to mutations in genes encoding N-methyltransferases^29^. This agronomically important trait could be transferred to *C. canephora* through CRISPR/Cas9 technology to create naturally decaffeinated plants. Recent studies have been performed to improve gene editing methodologies, such as using other CRISPR-Cas systems and applying ribonucleoproteins (RNPs) for DNA-free genome editing^30,31^. Those strategies may accelerate the generation of new varieties in the market since they would not be considered genetically modified organisms in many countries and could be commercially released more quickly.

Phytoene desaturase is an essential plant biosynthetic protein that catalyzes the addition of two double bonds to 15-cis-phytoene, generating 9,15,9′-tri-cis-ζ-carotene through the intermediate 9,15-di-cis-phytofluene^32^. The loss of function of this gene results in albino plant production, which is easily detectable by the naked eye and has been used in research to facilitate the establishment of gene editing protocols. The *C. canephora CcPDS* gene is 7042 bp long with 14 exons, and the targets for gene silencing were designed for exons 3 and 5. Most of the small mutations observed for both targets occurred three to four nucleotides upstream of the Proto-spacer Adjacent Motif (PAM) (Fig. 2). They probably resulted from the repair of nonhomologous ends (NHEJ), as observed in previous studies^19,20^. Some small, induced mutations generated changes in the reading frame and premature stop codons (Fig. 3). When they were present in both alleles (homozygous or biallelic), they promoted the development of the homogeneous albino phenotype. Larger deletions such as those observed in plants P5 and S2 could result from alternative mechanisms to the canonical NHEJ (cNHEJ). They are known as backup-NHEJ (b-NHEJ), alternative-NHEJ (a-NHEJ), and microhomology-mediated end joining (MMEJ), which could take place in the absence of cNHEJ factors and lead to larger deletions^33^.

The breeding of coffee can primarily benefit from the adoption of the strategy presented in this work, as genetic knowledge, and genomic resources for *C. canephora*^34^ already exist and can be combined with what has already been explored in other plant species to generate targets for gene editing. The clearest example of this applicability would be the loss of function of enzymes involved in the last three steps of caffeine biosynthesis, which are *N*-methyltransferases with high similarity encoded by already identified genes^34–36^. This strategy could result in selected varieties producing naturally decaffeinated beans to meet the demand more simply and efficiently for decaffeinated coffee compared to the industrial decaffeination process. Additionally, regarding the sensory attributes of coffee, modifications to ORFs (open reading frames) located in the untranslated regions (uORFs) of bZIP gene family members may allow changes in the sugar content in fruits, as demonstrated in strawberry^37^. In addition to these opportunities, it is worth emphasizing the possibilities of changing the plant architecture and flowering regulation, which are also crucial for the cultivation of coffee and have already been explored in gene editing approaches in other species, mainly tomatoes^38–40^.

In the case of variegated plants, such as S4, the mutations probably occurred after the early cell divisions during the formation of somatic embryos. Thus, generating plantlets with different sets of cells (with and without mutations) and the number of sequenced clones made it impossible to detect all these variations. Nevertheless, the formation of phenotypic and genotypic chimeras for mutations in the *PDS* gene has already been observed in different plant species^41,42^. One of the main reasons for the occurrence of these chimeras is the regeneration strategy involving organogenesis, but the progressive activity of Cas9 can further enhance this phenomenon during the regeneration process^43^. In addition to demonstrating a reduction in the number of chimeras through an extra regeneration step using first-generation transgenic plants, Malabarba et al.^43^ observed new editing patterns in the second generation of plants. The non-detection of these profiles confirmed it at initial analyses since Cas9 and sgRNAs continued to be expressed, and endonuclease activity was maintained as long as there were unedited cells. Regarding the seedlings with the variegated phenotype S1 and S3, both had a heterozygous mutation in only one of the targets (target 2 and target 1, respectively). Thus, we infer that at least one of the alleles did not suffer any mutation, which allowed the gene to be expressed and the function of the phytoene desaturase enzyme to remain normal.

The mutation rates observed in the perennial cultures explored in methodology establishment studies with the *PDS* gene as the target vary depending on the species. For example, *Vitis vinifera* had 30.6%^44^, 35% in *Citrus sinensis* Osbeck^45^, 30% in *C. canephora*^20^, 52% in *Populus tomentosa*^19^, 84% in *Malus x domestica* Bork.^41^, and *Gossypium hirsutum* ​​between 66 and 100%^46^. Moreover, using promoters from the species instead of those from *Arabidopsis thaliana* and adopting multiplex strategies seemed to improve the mutation probability in the target region^41^. Additionally, the delivery strategy of the genome editing components to the plant cell and its efficiency, as well as the selection of suitable target sites and the design of efficient sgRNAs, are factors that affect the success of generating point mutations^47^. In this study, 77% of the analyzed seedlings had some mutation in at least one allele of the *CcPDS* gene. This mutation rate shows this process's applicability for improving *C. canephora* and, in particular, clone 14. This clone has important characteristics for coffee cultivation, such as resistance to the nematodes which cause the most damage in coffee plantations, *Meloidogyne exigua* and *M. incognita*^14^, and drought tolerance^8,11^. Coupled with the possibility of modifying characteristics associated with the beverage quality, such as those already mentioned in this work, via CRISPR/Cas9, we argue that coffee breeding programs could also invest in *C. canephora* genotypes with desirable characterized agronomic traits such as the one explored in this work. *C. canephora* possesses adaptive characteristics with an unknown genetic base which are essential for the sustainable maintenance of the coffee production chain.

CRISPR/Cas9 technology has revolutionized plant breeding by faster developing plant ideotypes, either by deleting genetic elements responsible for undesirable characteristics or by introducing mutations to gain function. Through the application of this technology, it is possible to improve productivity, food/beverage quality, and resistance to diseases and herbicides^16^. The speed of obtaining traits of interest is desirable for improving perennial species such as coffee, reducing the time to obtain an improved variety from 25 to 30 years to approximately six years^20^. Our results support the efficiency of this technology in *C. canephora*, demonstrating not only the editing of the *CcPDS* gene but also the generation of the expected phenotype for these mutations. Obtaining such results with an essential genotype with important desirable traits such as drought tolerance and nematode multi-resistance is an important step towards developing new varieties for coffee cultivation through gene editing technology.

## Methods

### Plant material

The leaves used as explants were derived from plants cultivated at the Federal University of Lavras greenhouse, part of the Central Laboratory of Molecular Biology (LCBM), where the first author conducted the experiments. These cultivated plants were derived from cuttings propagated clonally from *C. canephora* varieties which the authors have the rights and all permissions to explore. *Coffea canephora* embryogenic calli were obtained from clone 14 leaves from greenhouse-growing plants and were superficially disinfected in a laminar flow chamber. Next, the edges and midrib were removed, and explants containing secondary ribs measuring approximately 1 cm^2^ were inoculated onto Yasuda semi-solid medium^48^. After nine months, embryogenic calli were isolated from the initial explants and subcultured onto Yasuda semi-solid medium every three weeks. These calli were then used in the transformation procedure. During the entire embryogenic callus induction and multiplication process, the explants were cultivated in sterile disposable Petri dishes in the dark in a growth room at 25 ± 2 °C.

### Binary vector

The transformation vector pCOFEDIT-2^20^ was used. It was obtained by cloning the plant-optimized synthetic sequence of the Cas9 enzyme (KR154349.1—a synthetic construct of the *S. pyogenes* Cas9 gene^49^) and the sequences of the PDS3 sgRNA guide (GCAAAGTATTTGGCCGATGCAGG) and PDS5 (GAATTAATGATCGGTTGCAGTGG) (GenScript, HK Limited, Hong Kong) in the binary vector pCAMBIA 5300^50^.

### Co-cultivation with *Agrobacterium* and selection of putatively transformed embryogenic calli

Embryogenic callus transformation mediated by *A. tumefaciens* strain EHA105 followed the protocol established by Ribas et al.^21^, with minor modifications. This method consists of incubating the embryogenic calli with the bacterial suspension at an OD of 0.6–0.8 (adding 200 µM acetosyringone) for 10 min, followed by co-cultivation for five days at 28 °C at obscurity, and finally, wash steps to remove traces of *A. tumefaciens* altogether. Then, the calli were transferred to 15 cm diameter Petri dishes containing Yasuda medium at 500 mg L^−1^ timentin and kept in the dark for four weeks in a growth room (25 ± 2 °C). After this period, the cultures were transferred to the same medium with 300 mg L^−1^ timentin and the selection agent hygromycin at 50 mg L^−1^ were kept under indirect light at approximately 2 µmol m^−2^ s^−1^. After four weeks, the hygromycin concentration was increased to 100 mg L^−1^, and the material was subcultured every four weeks using the same basal medium. Finally, after six months of callus selection, the selective agent was removed from the culture medium.

### Regeneration of transformed somatic embryos and plantlets

After regeneration, embryos were transferred to maturation medium^51^ and subcultured every four weeks until the complete expansion of the cotyledons. The embryos were then transferred to glass flasks containing a rooting induction medium, which consisted of MS salts^52^ with 100 mg L^−1^ adenine, 400 mg L^−1^ malt extract, and 30 g L^−1^ sucrose without the addition of growth regulators and solidified with 2.5 g L^−1^ Phytagel ™ (Sigma–Aldrich). The germinated somatic embryos and somatic seedlings were subcultured every 30 days. After rooting, the seedlings were transferred to T6 medium^53^, where they were maintained for further analysis; Two-to-five leaf pair seedlings were conserved by repetitive transfers onto fresh medium every 60 days.

### Genomic DNA extraction and PCR

Whole putatively transformed somatic embryos or leaves from in vitro grown putatively transformed somatic were collected for genomic DNA extraction. Since the amount of plant material available was quite limited, TissueLyserLT (QIAGEN) equipment was used to grind the samples. For DNA extraction, a protocol using CTAB (cationic hexadecyl trimethyl ammonium bromide) buffer^54^ was followed, with minor modifications. First, the CTAB isolation buffer (2% CTAB [Sigma H-5882], 1.4 M NaCI, 0.2% 2-mercaptoethanol, 20 mM EDTA, 100 µM Tris-HCI, pH 8.0) was added to the microtubes containing the ground plant tissue in a volume of 1.7 mL. Then, after the precipitation of the DNA in isopropanol at − 20 °C for 1 h, the pellet was rinsed twice with 70% ethanol, vacuum-dried, dissolved in 25 µl UltraPure™ DNase/RNase-Free Distilled Water (Invitrogen), quantified by NanoDrop™ (ThermoFisher) and stored at − 20 °C until its use in the following analysis.

To confirm the insertion of the T-DNA in the genome of *C. canephora* somatic seedlings/embryos, PCR was performed using primers targeting the *Cas9* gene, with the following sequences: Fw1: 5′ TATTCACGGGGTGCCTGCGG 3′; Rv1: 5′ TGTGCCAGAGCGAGGTAGATCA 3′.

### Detection of mutations

Genomic DNA previously extracted from putatively transgenic, and control somatic plantlets and embryos were used to amplify a region of approximately 500 bp containing the target region of the sgRNAs, using specific primers. For Target 1, located in exon 3, the primers used were Fv1: TGAAAGGTTTCGTCACTGTACATGCA and Rv1: TGCACACATATAGACCAACTCCCACA, with an annealing temperature of 66 °C. For Target 2, in exon 5, the primers used were Fw2: ATTTGCCTTATCTTGTTCCGTGCTT and Rv2: TGGTTGCATACTTGCTTTCTCATCC, with an annealing temperature of 69 °C.

PCRs were performed using the Phusion High-Fidelity DNA Polymerase (Thermo Scientific). PCR products were cloned in pGEM®-T Easy Vector Systems (Promega) and propagated on *E. coli*. Mutations were identified by Sanger sequencing of individual clones (ACTGene Molecular Analysis), with four clones from each target region being sequenced for each sample.

Using the combination of sequences obtained from putative mutant somatic seedlings/embryos, wild-type plants, and known references (Cc04_g00540 from Coffee Genome Hub, http://coffee-genome.org/) was used ClustalW alignment with MEGA-X software^55^. The images of the alignment results were made using GeneDoc software^56^.

## Supplementary Information

Supplementary Information.

## Data Availability

All data generated or analyzed during this study are included in this published article (and its Supplementary Information files).
