# Supplementary material for: Multiplex CRISPR/Cas9-mediated knockout of the phytoene desaturase gene in Coffea canephora
Source: Sci Rep. 2022 Oct 14;12:17270. doi: 10.1038/s41598-022-21566-w (PMC9568650; doi:10.1038/s41598-022-21566-w)
Supplement: Supplementary file 1 — Supplementary Information. [file 41598_2022_21566_MOESM1_ESM.pdf]

Multiplex CRISPR/Cas9-mediated knockout of the *phytoene desaturase* gene in *Coffea canephora*

Tatiane Casarin[1*], Natália Chagas Freitas[1], Renan Terassi Pinto[1], Jean-Christophe Breitler[2,3], Leonardo Augusto Zebral Rodrigues[1], Pierre Marraccini[2,3], Hervé Etienne[2,3], Leandro Eugenio Cardamone Diniz[4], Alan Carvalho Andrade[5], Luciano Vilela Paiva[1*]

1 LCBM, UFLA, Lavras, 3037 Brazil;

2 CIRAD, UMR DIADE, Montpellier, F-34398, France;

3 UMR DIADE, University of Montpellier, CIRAD, IRD, Montpellier, F-34398, France.

4 Embrapa Soybean, Londrina, 86085-981, Brazil;

5 Embrapa Coffee - Inovacafé, Lavras, 3037, Brazil;

*casarintatiane@gmail.com; luciano@ufla.br

Supplementary Information

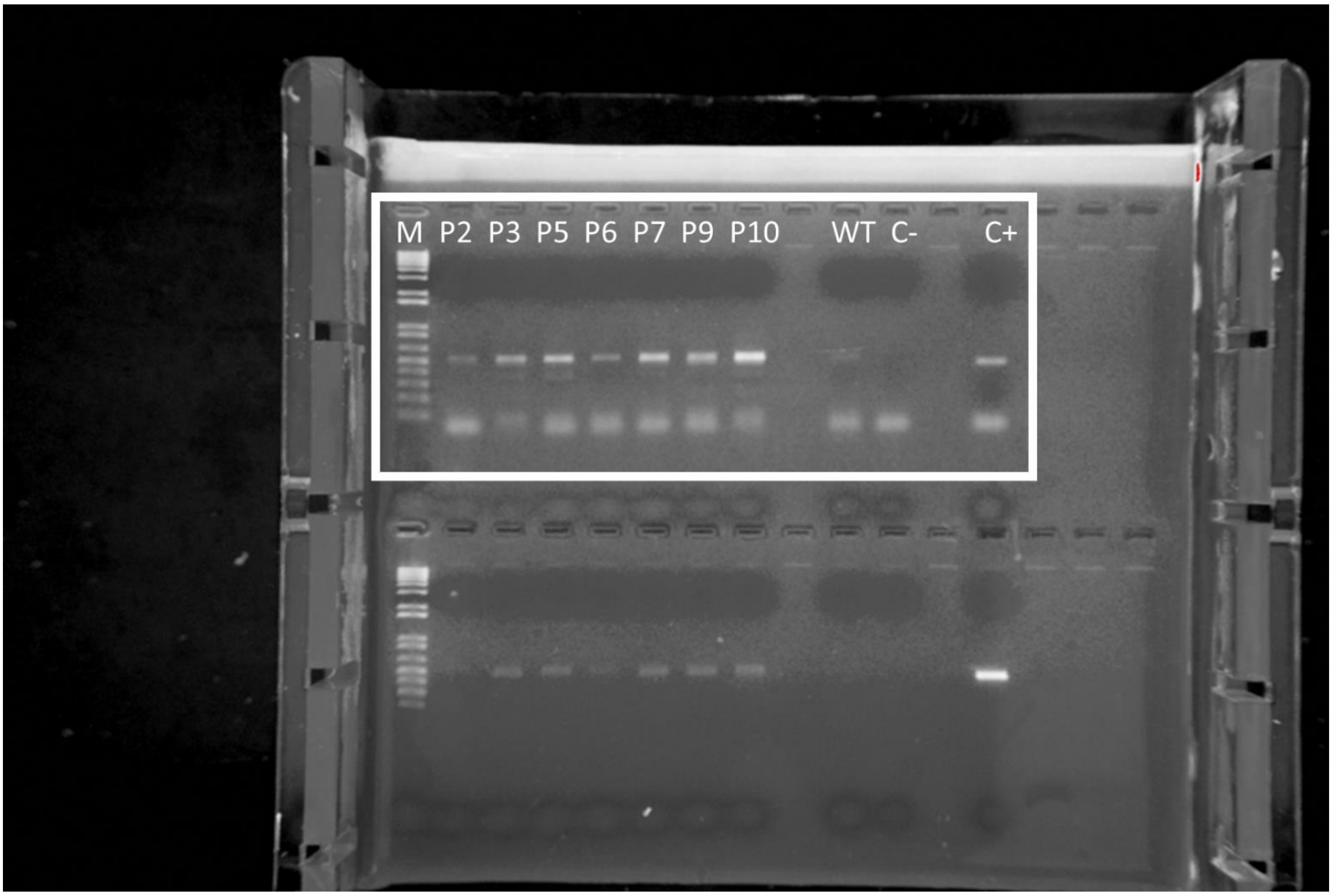

Supplementary fig. S1. Unprocessed image of the electrophoretic gel of the PCR to confirm the transformation of *C. canephora* albino seedlings with the pCOFEDIT-2 vector. In the area delimited by white lines, it is possible to observe

the following: M: molecular weight marker; P2-P10: transformed seedlings; WT: WT seedling; C-: negative control (water); C+: positive control (plasmid DNA).

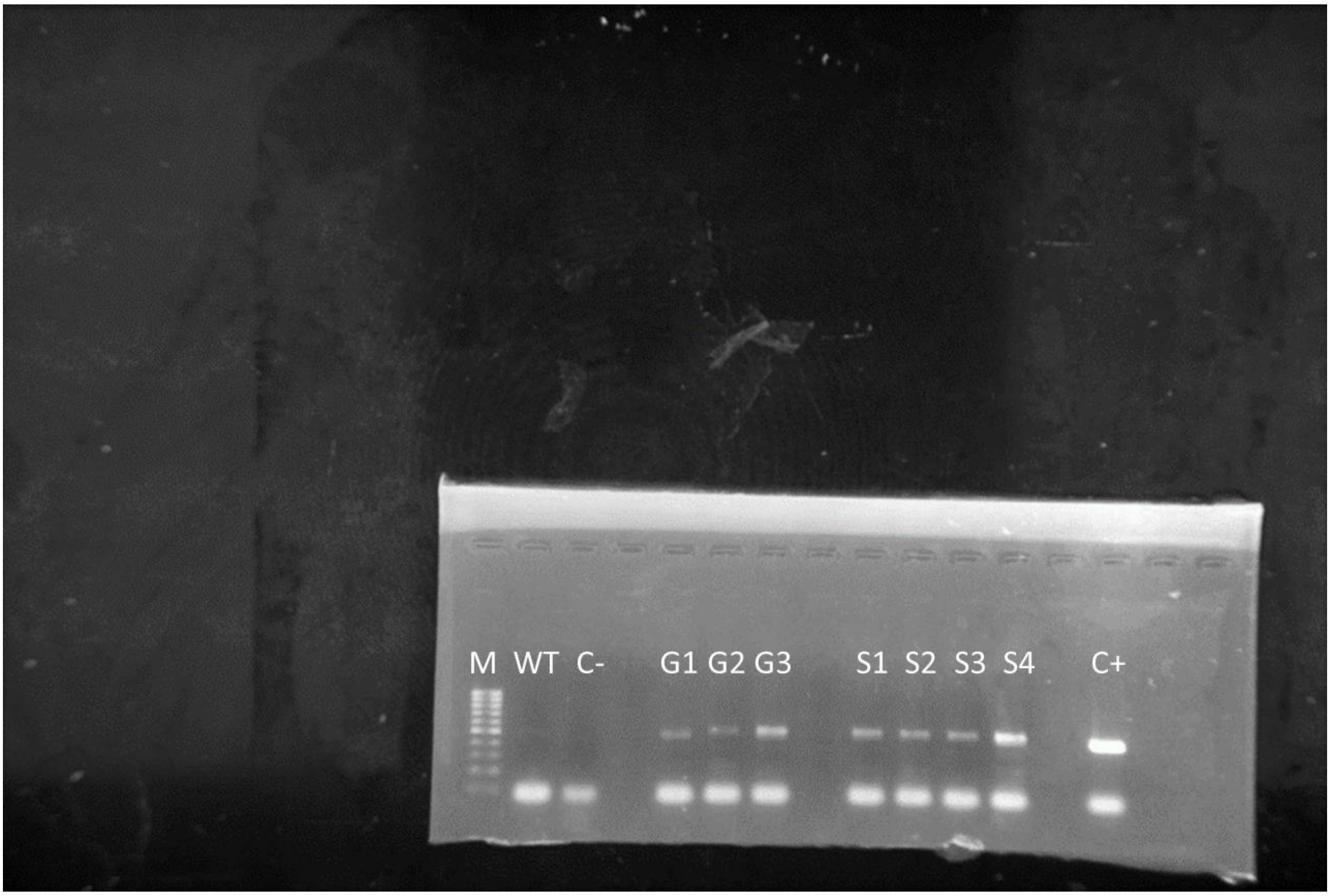

Supplementary fig. S2. Unprocessed image of the electrophoretic gel of the PCR to confirm the transformation of *C. canephora* green and variegated seedlings with the pCOFEDIT-2 vector. Legend: M: molecular weight marker; WT: WT seedling; C-: negative control (water); G1-G3: green seedlings; S1-S4: variegated seedlings; C+: positive control (plasmid DNA).

Fasta sequences of the sequencing results used to analyze the mutations generated in the CcPDS gene.

Supplementary dataset 1: Partial genomic DNA sequencing of *CcPDS* corresponding to sgRNA target 1 (exon 3). Abbreviations refer to the following samples: P: albino somatic seedlings, WT: control, untransformed; G: green seedlings (transformed); S: variegated seedlings (transformed); and C: different clones sequenced.

>P2.C3
AGGSCCTKKGKGRAAKKAAWTTTGGGGCTGTCTTATTTAGGAGTCCTATCAGATGATTATCTGGTTTGCAAGACGGATCA
CTTTTTATAGCTGATATTTTATATGTTTTAGCCTCCATTAGAACCTATGTTGTCTTATTTTGGTATTTTGTCATAAATTT
GTATCATCGGATGTTATAAGTCAATTGCTTCTGAAAATAAGTCAAGGTATGACATACAGAACAAAGTCTGTTATGAAATA
AATTTCCACTTACTTGATTAAGTTTTATACTTTCAGGTTTGGCTGGTTTGTCTACTGCAAAGTATTTGGCCGATGCAGGT
CATAAACCTATAGTGTTGGAAGCTAGGGATGTTCTGGGAGGAAAGGTAGCCAAATTATTACTCATTAGTGTTCATGAATT
CCTTGTGGCATAATGGACTGTGTCAAAGTTCAAGGAAAGTCTTTCAAAATTTTCCAGTATATGGATGTGGGAGTTGGTCT
ATATGTGTGCAAATCGAATTCCCGCGGCCGCCATGGCGGCCGGGAGCATGCGACGTCGGGCCCAATTCGCCCTATAGTGA
GTCGTATTACAATTCACTGGCCGTCGTTTTACAACGTCGTGACTGGGAAAACCCTGGCGTTACCCAACTTAATCGCCTTG
CAGCACATCCCCCTTTCGCCAGCTGGCGTAATAGCGAAGAGGCCCGCACCGATCGCCCTTCCCAACAGTTGCGCAGCCTG
AATGGCGAATGGACGCGCCCTGTAGCGGCGCATTAAGCGCGGCGGGTGTGGTGGTTACGCGCAGCGTGACCGCTACACTT
GCCAGCGCCCTAGCGCCCGCTCCTTTCGCTTTCTTCCCTTCCTTTCTCGCCACGTTCGCGGGCTTTCCCGGTMAGCTCTA
ATCGGGGGGCTCCCTTTAGGGTTCCGATTAGTGCCTTTACGGCACTCGACCCCCAAAAACTTGGAATTWRGGGGGKGAAT
GGGTTYTMSAASG
>P2.C4
GGGCTGTATGATTTGGGGTGCTTTTAGGATCTATCAATGTTTCTGGTTGCAACGGTCCTTTTTAACTGTATTTAATGTTT
ACCTCCTTAAACTATGTGCTATTTGTATTTGCTAATTTGACTCGGTGTAAATCATTGTTCTGAAAATCAGTTGCTAAAAA
ATCTGTTGAAAATTTCATTATTGATATTTAATTTAGTTGGTGGTTGCATGAAKATTGGCGTGAGCTAACTAATGTGAACA
GGTGTTGGAGAAGGACCATTATATCTTATGTCTATTCTTGGCTATGATGGCAATTCAGGAATCTTCAATTTCATTTGGTG
GGATTGGCAATTGGAATCAATCCCGGCCCTGCGCCGGACTCACTCGCCATCCCTAATGGCKATAATTATGGCTCTTAACT
CTGCGGAACCTGCTTCCATATCCCTGACCTCCTTCCACGCTAACAAAGCCCCACCCTCACKTCCCTGAGCATGCCCCGAC
GCTACCGGGGGGTCCCCTGCCAATGACCCACCCCCTCTTTCTCTCCCCTCCGTCCKASCACGTCTAGCATAGTCGCCCCA
ATAAGAGCKTGCCCAAGTCCTRTGCTTATATTCYAACCYKTTTAGTCTSTGARTATCTAAATTRTCCSTCMGTASCTAAC
AAAAACTSTCAATACWCGMMSS
>P2.C5
GTKKRATRAWTTGGGGTKCTTTTTAGGATCTATCAGTGTTATCTGGTTGCAGACGGTCCTTTTTAACTGTTTTTAATGTT
TACCTCCTTAAACCTATGTGCTATTTTGGATTTGCTAAATTGATCTCGGTGTTAATCATTGTTCTGAAAAATCAGTTGCT
AAAACAATCTGTTGAAAATTTCATTATTGATATTTAACTTAGTTGGTGGTTGCATGAATATTGCCGTGAGCTAACTAATG
TGAACAGGTGTCTGGAGAAGGACCATTATATCTTATGTCTGATTCTTGGCTATGATGGCAATTCAGGAATCTTCAATTTT
CATAATGGTGGGATTGTCAATTGGAATCAATCCCGCCCCTGCGCCGGACTCACTCGGCCATCCCTAATATCTATAATTAT
GGCTCTTAACTCTGCGGAACCTGCTTCCATATCCCTGACCTCCTTCCACGCTAACAAAGCCCCACCCTCACTTCCCCGTG
CATGCCCCGACCCTACCGGGGGGTCCCCTGCCAATGACCCACCCCCTCTTTCTCTCCCCTCCGTCCKASYACGCCTAGCA
TTGTCGCCCCAATGAGAGCKTGCCCAAGTYCTRTGCTTAAGCTAGAYCWCKTTTAGTCTSTKARTATCTAAAYTRTYYGM
MGTGSCTAWCAAAAATTTCAGCTTGARRR
>P2.C6
TGAAKCAKCKATGATTTGGGGCTGTCTTATTTAGGAGTCCTATCAGATGATTATCTGGTTTGCAAGACGGATCACTTTTT
ATAGCTGATATTTTATATGTTTTAGCCTCCATTAGAACCTATGTTGTCTTATTTTGGTATTTTGTCATAAATTTGTATCA
TCGGATGTTATAAGTCAATTGCTTCTGAAAATAAGTCAAGGTATGACATACAGAACAAAGTCTGTTATGAAATAAATTTC
CACTTACTTGATTAAGTTTTATACTTTCAGGTTTGGCTGGTTTGTCTACTGCAAAGTATTTGGCCGATGCAGGTCATAAA
CCTATAGTGTTGGAAGCTAGGGATGTTCTGGGAGGAAAGGTAGCCAAATTATTACTCATTAGTGTTCATGAATTCCTTGT
GGCATAATGGACTGTGCCAAAGTTCAAGGAAAGTCTTTCAAAATTTTCCAGTATATGGATGTGGGAGTTGGTCTATATGT
GTGCAAATCGAATTCCCGCGGCCGCCATGGCGGCCGGGAGCATGCGACGTCGGGCCCAATTCGCCCTATAGTGAGTCGTA
TTACAATTCACTGGCCGTCGTTTTACAACGTCGTGACTGGGAAAACCCTGGCGTTACCCAACTTAATCGCCTTGCAGCAC
ATCCCCCTTTCGCCAGCTGGCGTAATAGCGAAGAGGCCCGCACCGATCGCCCTTCCCAACAGTTGCGCAGCCTGAATGGC
GAATGGACGCGCCCTGTAGCGGCGCATTAAGCGCGGCGGGTGTGGTGGTTACGCGCAGCGTGACCGCTACACTTGCCAGC
GCCCTAGCGCCCGCTCCTTTCGCTTTCTTCCCTTTCCTTTCTCGCCACGTTCGCCGGCTTTCCCGTCAGGCTCTAAATCG
GGGGGCTCCCGTTTAGGGATTTCCGAATTTTAGTKGCCTTTTAACGGCMAC
>P3.C5

TGATGCAGTGATGATTTGGGGCTGTCTTATTTAGGAGTCCTATCAGATGATTATCTGGTTTGCAAGACGGATCACTTTTT
ATAGCTGATATTTTATATGTTTTAGCCTCCATTAGAACCTATGTTGTCTTATTTTGGTATTTTGTCATAAATTTGTATCA
TCGGATGTTATAAGTCAATTGCTTCTGAAAATAAGTCAAGGTATGACATACAGAACAAAGTCTGTTATGAAATAAATTTC
CACTTACTTGATTAAGTTTTATACTTTCAGGTTTGGCTGGTTTGTCTACTGCAAAGTATTTGGCCGATGCAGGTCATAAA
CCTATAGTGTTGGAAGCTAGGGATGTTCTGGGAGGAAAGGTAGCCAAATTATTACTCATTAGTGTTCATGAATTCCTTGT
GGCATAATGGACTGTGTCAAAGTTCAAGGAAAGTCTTTCAAAATTTTCCAGTATATGGATGTGGGAGTTGGTCTATATGT
GTGCAAATCGAATTCCCGCGGCCGCCATGGCGGCCGGGAGCATGCGACGTCGGGCCCAATTCGCCCTATAGTGAGTCGTA
TTACAATTCACTGGCCGTCGTTTTACAACGTCGTGACTGGGAAAACCCTGGCGTTACCCAACTTAATCGCCTTGCAGCAC
ATCCCCCTTTCGCCAGCTGGCGTAATAGCGAAGAGGCCCGCACCGATCGCCCTTCCCAACAGTTGCGCAGCCTGAATGGC
GAATGGACGCGCCCTGTAGCGGCGCATTAAGCGCGGCGGGTGTGGTGGTTACGCGCAGCGTGACCGCTACACTTGCCAGC
GCCCTAGCGCCCGCTCCTTTCGCTTTCTTCCTTCCTTTCTCGCCACGTTCGCCGGCTTCCCGTCAAGCTCTAATCGGGGC
TCCCTTTAGGTTCCGATTTAGKGGCTTTACGGCACCCTCCGAMCCCCCAGTATAC
>P3.C6
TCATGCAKCKATGATTTGGGGCTGTCTTATTTAGGAGTCCTATCAGATGATTATCTGGTTTGCAAGACGGATCACTTTTT
ATAGCTGATATTTTATATGTTTTAGCCTCCATTAGAACCTATGTTGTCTTATTTTGGTATTTTGTCATAAATTTGTATCA
TCGGATGTTATAAGTCAATTGCTTCTGAAAATAAGTCAAGGTATGACATACAGAACAAAGTCTGTTATGAAATAAATTTC
CACTTACTTGATTAAGTTTTATACTTTCAGGTTTGGCTGGTTTGTCTACTGCAAAGTATTTGGCCGATGCAGGTCATAAA
CCTATAGTGTTGGAAGCTAGGGATGTTCTGGGAGGAAAGGTAGCCAAATTATTACTCATTAGTGTTCATGAATTCCTTGT
GGCATAATGGACTGTGTCAAAGTTCAAGGAAAGTCTTTCAAAATTTTCCAGTATATGGATGTGGGAGTTGGTCTATATGT
GTGCAAATCGAATTCCCGCGGCCGCCATGGCGGCCGGGAGCATGCGACGTCGGGCCCAATTCGCCCTATAGTGAGTCGTA
TTACAATTCACTGGCCGTCGTTTTACAACGTCGTGACTGGGAAAACCCTGGCGTTACCCAACTTAATCGCCTTGCAGCAC
ATCCCCCTTTCGCCAGCTGGCGTAATAGCGAAGAGGCCCGCACCGATCGCCCTTCCCAACAGTTGCGCAGCCTGAATGGC
GAATGGACGCGCCCTGTAGCGGCGCATTAAGCGCGGCGGGTGTGGTGGTTACGCGCAGCGTGACCGCTACACTTGCAGCG
CCCTAGCGCCCGCTCCTTTTCGCTTTCTCCCTTTCCTTTCYCGCCACGTTCGGCCGGCTTCCCCGTCAAGCTCTAAATCG
GGGGCTCCGTTAGGGGTTCCGATTAGTGCCTTACCGGCCACCCTTCGAACYCCCAAAKAAAA
>P3.C7
TRKGCTGTGATGATTTGGGGTGCTTTTTAGGATCTATCAATGATATCTGGTTGCAGACGGTCCTTTTTAACTGTATTTAT
ATGTTTACCTCCTTAAACCTATGTTGTCTTATTTTGGTATTTTGTCATAAATTTGTATCATCGGATGTTATAAGTCAATT
GCTTCTGAAAATAATCAAGGTATGACATACAGAACAAATCTGTATGAATAAATTTCCACTTACTTGTTAATTTTATACTT
TCAGGTTGGTGGTTGCTACTGCAAATATTTGGCGATGCAGTCTAAACCTATATGTGGAACTAGGGTGTCTGGGAGGAAAG
GACCAATTATTACTCTTATGTCTGATTCCTTGTGCTATGATGTGCAATTCAGGAAATCTTCAATTTTCATAATGGTGGGA
TTGGCTTTTGGAACAATCCCGCCCCTGCGCCGGACTCACTCGCCATCCCTAATATCKATAATTATGGCTCTTTCACTCTG
CGGAACCTGCTACACTATCCCTGACCTCCCTCCACGCTAACAAAGCCCCACCCTCCAATGCACTGTGCAGGCCCCGACGC
TACCGGGGGGTCCCCKGCCAATGACCCACSCCCTTCTTTCTCTTCCMTCCGTCCKACYACGCCTAGCATTGTCGCCCCAA
TTAGAGCAGCCCAAWTMCTATACTTAWAYCAMACCCMRTTAAG
>P3.C8
TAKKGCAKCKATGATTTGGGGCTGTCTTATTTAGGAGTCCTATCAGATGATTATCTGGTTTGCAAGACGGATCACTTTTT
ATAGCTGATATTTTATATGTTTTAGCCTCCATTAGAACCTATGTTGTCTTATTTTGGTATTTTGTCATAAATTTGTATCA
TCGGATGTTATAAGTCAATTGCTTCTGAAAATAAGTCAAGGTATGACATACAGAACAAAGTCTGTTATGAAATAAATTTC
CACTTACTTGATTAAGTTTTATACTTTCAGGTTTGGCTGGTTTGTCTACTGCAAAGTATTTGGCCGATGCAGGTCATAAA
CCTATAGTGTTGGAAGCTAGGGATGTTCTGGGAGGAAAGGTAGCCAAATTATTACTCATTAGTGTTCATGAATTCCTTGT
GGCATAATGGACTGTGTCAAAGTTCAAGGAAAGTCTTTCAAAATTTTCCAGTATATGGATGTGGGAGTTGGTCTATATGT
GTGCAAATCGAATTCCCGCGGCCGCCATGGCGGCCGGGAGCATGCGACGTCGGGCCCAATTCGCCCTATAGTGAGTCGTA
TTACAATTCACTGGCCGTCGTTTTACAACGTCGTGACTGGGAAAACCCTGGCGTTACCCAACTTAATCGCCTTGCAGCAC
ATCCCCCTTTCGCCAGCTGGCGTAATAGCGAAGAGGCCCGCACCGATCGCCCTTCCCAACAGTTGCGCAGCCTGAATGGC
GAATGGACGCGCCCTGTAGCGGCGCATTAAGCGCGGCGGGTGTGGTGGTTACGCGCAGCGTGACCGCTACACTTGCCAGC
GCCCTAGCGCCCGCTCCTTTCGCTTTCTTCCCTTCCTTTCTCGCCACGTTCGCCGGCTTTCCCCGTCAAGCTCTAATCGG
GGGGCTCCCGTTTAAGGTTTCCGATTTAGTGCCTTTTACGGCMCCCCYYCGACCCCCAGAAAA
>P6.C1
TKAWGGCTKKATGATTTGGGGCTGTCTTATTTAGGAGTCCTATCAGATGATTATCTGGTTTGCAAGACGGATCACTTTTT
ATAGCTGATATTTTATATGTTTTAGCCTCCATTAGAACCTATGTTGTCTTATTTTGGTATTTTGTCATAAATTTGTATCA
TCGGATGTTATAAGTCAATTGCTTCTGAAAATAAGTCAAGGTATGACATACAGAACAAAGTCTGTTATGAAATAAATTTC
CACTTACTTGATTAAGTTTTATACTTTCAGGTTTGGCTGGTTTGTCTACTGCAAAGTATTTGGCTGCAGGTCATAAACCT
ATAGTGTTGGAAGCTAGGGATGTTCTGGGAGGAAAGGTAGCCAAATTATTACTCATTAGTGTTCATGAATTCCTTGTGGC
ATAATGGACTGTGCCAAAGTTCAAGGAAAGTCTTTCAAAATTTTCCAGTATATGGATGTGGGAGTTGGTCTATATGTGTG
CAAATCGAATTCCCGCGGCCGCCATGGCGGCCGGGAGCATGCGACGTCGGGCCCAATTCGCCCTATAGTGAGTCGTATTA
CAATTCACTGGCCGTCGTTTTACAACGTCGTGACTGGGAAAACCCTGGCGTTACCCAACTTAATCGCCTTGCAGCAMATC

CCCCTTTCGCCAGCTGGCGTAATAGCGAAGAGGCCCGCACCGATCGCCCTTCCCAACAGTTGCGCAGCCTGAATGGCGAA
TGGACGCGCCCTGTARCGGCGCATTAAGCGCGGCGGGGTGTGGTGGTTACGCGCAGCGTGACCGCTACACTTGCCAGCGC
CCTAGCGCCCGCTCCTTTCGCTTTTCTTCCCTTCTTTCTCGCCACGTTCGCCGGCTTTCCCGWMAGMTCWAATCAGGGGG
GCTCCCMTATAGGGTTCMGAATTTAGATGCCTTTACGGSACMCCYTCTSAACCCCCCATAYAATAG
>P6.C2
TWRKKCCKKCGKATGATTTGGGGCTGTCTTATTTAGGAGTCCTATCAGATGATTATCTGGTTTGCAAGACGGATCACTTT
TTATAGCTGATATTTTATATGTTTTAGCCTCCATTAGAACCTATGTTGTCTTATTTTGGTATTTTGTCATAAATTTGTAT
CATCGGATGTTATAAGTCAATTGCTTCTGAAAATAAGTCAAGGTATGACATACAGAACAAAGTCTGTTATGAAATAAATT
TCCACTTACTTGATTAAGTTTTATACTTTCAGGTTTGGCTGGTTTGTCTACTGCAAAGTATTTGGCCGATGCAGGTCATA
AACCTATAGTGTTGGAAGCTAGGGATGTTCTGGGAGGAAAGGTAGCCAAATTATTACTCATTAGTGTTCATGAATTCCTT
GTGGCATAATGGACTGTGCCAAAGTTCAAGGAAAGTCTTTCAAAATTTTCCAGTATATGGATGTGGGAGTTGGTCTATAT
GTGTGCAAATCGAATTCCCGCGGCCGCCATGGCGGCCGGGAGCATGCGACGTCGGGCCCAATTCGCCCTATAGTGAGTCG
TATTACAATTCACTGGCCGTCGTTTTACAACGTCGTGACTGGGAAAACCCTGGCGTTACCCAACTTAATCGCCTTGCAGC
ACATCCCCCTTTCGCCCAGCTGGGCGTAATAGCGAAASAGGCCCGCACCGATCGCCCCTTCCCAACAGTTGCGCA
>P6.C3
TGAATGCATGCKATGATTTGGGGCTGTCTTATTTAGGAGTCCTATCAGATGATTATCTGGTTTGCAAGACGGATCACTTT
TTATAGCTGATATTTTATATGTTTTAGCCTCCATTAGAACCTATGTTGTCTTATTTTGGTATTTTGTCATAAATTTGTAT
CATCGGATGTTATAAGTCAATTGCTTCTGAAAATAAGTCAAGGTATGACATACAGAACAAAGTCTGTTATGAAATAAATT
TCCACTTACTTGATTAAGTTTTATACTTTCAGGTTTGGCTGGTTTGTCTACTGCAAAGTATTTGGCCGATGCAGGTCATA
AACCTATAGTGTTGGAAGCTAGGGATGTTCTGGGAGGAAAGGTAGCCAAATTATTACTCATTAGTGTTCATGAATTCCTT
GTGGCATAATGGACTGTGCCAAAGTTCAAGGAAAGTCTTTCAAAATTTTCCAGTATATGGATGTGGGAGTTGGTCTATAT
GTGTGCAAATCGAATTCCCGCGGCCGCCATGGCGGCCGGGAGCATGCGACGTCGGGCCCAATTCGCCCTATAGTGAGTCG
TATTACAATTCACTGGCCGTCGTTTTACAACGTCGTGACTGGGAAAACCCTGGCGTTACCCAACTTAATCGCCTTGCAGC
ACATCCCCCTTTCGCCAGCTGGCGTAATAGCGAAGAGGCCCGCACCGATCGCCCTTCCCAACAGTTGCGCAGCCTGAATG
GCGAATGGACGCGCCCTGTAGCGGCGCATTAAGCGCGGCGGGTGTGGTGGTTACGCGCAGCGTGACCGCTACACTTGCCA
GCGCCCTAGCGCCCGCTCCTTTCGCTTTCTTCCTTCCTTTCTCGCACGTTCGCGGCTTCCCGTCAGCTCTAAATCGGGGC
TCCTTAGGGTTCCGATTAGTGCTTTACCGGCCACCCTCGAACCCCAAAAAAAMTCT
>P6.C4
TKGAKGCTGCKATGATTTGGGGCTGTCTTATTTAGGAGTCCTATCAGATGATTATCTGGTTTGCAAGACGGATCACTTTT
TATAGCTGATATTTTATATGTTTTAGCCTCCATTAGAACCTATGTTGTCTTATTTTGGTATTTTGTCATAAATTTGTATC
ATCGGATGTTATAAGTCAATTGCTTCTGAAAATAAGTCAAGGTATGACATACAGAACAAAGTCTGTTATGAAATAAATTT
CCACTTACTTGATTAAGTTTTATACTTTCAGGTTTGGCTGGTTTGTCTACTGCAAAGTATTTGGCTGCAGGTCATAAACC
TATAGTGTTGGAAGCTAGGGATGTTCTGGGAGGAAAGGTAGCCAAATTATTACTCATTAGTGTTCATGAATTCCTTGTGG
CATAATGGACTGTGTCAAAGTTCAAGGAAAGTCTTTCAAAATTTTCCAGTATATGGATGTGGGAGTTGGTCTATATGTGT
GCAAATCGAATTCCCGCGGCCGCCATGGCGGCCGGGAGCATGCGACGTCGGGCCCAATTCGCCCTATAGTGAGTCGTATT
ACAATTCACTGGCCGTCGTTTTACAACGTCGTGACTGGGAAAACCCTGGCGTTACCCAACTTAATCGCCTTGCAGCACAT
CCCCCTTTCGCCAGCTGGCGTAATAGCGAAGAGGCCCGCACCGATCGCCCTTCCCAACAGTTGCGCAGCCTGAATGGCGA
ATGGACGCGCCCTGTAGCGGCGCATTAAGCGCGGCGGGTGTGGTGGTTACGCGCAGCGTGACCGCTACACTTGCCAGCGC
CCTAGCGCCCGCTCCTTTCGCTTTCTTCCCTTCCTTTCTCGCCACGTTCGCCGGCTTCCGTCAGCTCTAAATCGGGGGCT
CCCTTTAAGGTTCCGATTTAGTGCCTTTTACGGCMCCCYTCSAAACMCCCCAAA
>P7.C1
TRAWKGCTKCKATGAATTTGGGGCTGTCTTATTTAGGAGTCCTATCAGATGATTATCTGGTTTGCAAGACGGATCACTTT
TTATAGCTGATATTTTATATGTTTTAGCCTCCATTAGAACCTATGTTGTCTTATTTTGGTATTTTGTCATAAATTTGTAT
CATCGGATGTTATAAGTCAATTGCTTCTGAAAATAAGTCAAGGTATGACATACAGAACAAAGTCTGTTATGAAATAAATT
TCCACTTACTTGATTAAGTTTTATACTTTCAGGTTTGGCTGGTTTGTCTACTGCAAAGTATTTGGCCGAGTGCAGGTCAT
AAACCTATAGTGTTGGAAGCTAGGGATGTTCTGGGAGGAAAGGTAGCCAAATTATTACTCATTAGTGTTCATGAATTCCT
TGTGGCATAATGGACTGTGCCAAAGTTCAAGGAAAGTCTTTCAAAATTTTCCAGTATATGGATGTGGGAGTTGGTCTATA
TGTGTGCAAATCGAATTCCCGCGGCCGCCATGGCGGCCGGGAGCATGCGACGTCGGGCCCAATTCGCCCTATAGTGAGTC
GTATTACAATTCACTGGCCGTCGTTTTACAACGTCGTGACTGGGAAAACCCTGGCGTTACCCAACTTAATCGCCTTGCAG
CACATCCCCCTTTCGCCAGCTGGCGTAATAGCGAAGAGGCCCGCACCGATCGCCCTTCCCAACAGTTGCGCAGCCTGAAT
GGCGAATGGACGCGCCCTGTAGCGGCGCATTAAGCGCGGCGGGTGTGGTGGTTACGCGCAGCGTGACCGCTACACTTGCC
AGCGCCCTAGCGCCCGCTCCTTTCGCTTTCTTCCTTCCTTTCTCGCCACGTTCGCCGGCTTCCCGTCAGCTCTAATCGGG
GCTCCTTAGGTTCCGATTTAGTGCTTTWTACCGGCACCTYCGAACCCYCAAAAAAAACT
>P7.C2
CKRARWWGCCTTKTGKGAATGATTTGGGGCTGTCTTATTTAGGAAGTCCTATCAGATGAATTATCTGGTTTGCAAGACGG
ATCMCTTTTTATAGCTGATATTTTATATGTTTTAGCCTCCATTAGAACCTATGTTGTCTTATTTTGGTATTTTGTCATAA
ATTTGTATCATCGGATGTTATAAGTCAATTGCTTCTGAAAATAAGTCAAGGTATGACATACAGAACAAAGTCTGTTATGA

AATAAATTTCCACTTACTTGATTAAGTTTTATACTTTCAGGTTTGGCTGGTTTGTCTACTGCAAAGTATTTGGCCGAGTG
CAGGTCATAAACCTATAGTGTTGGAAGCTAGGGATGTTCTGGGAGGAAAGGTAGCCAAATTATTACTCATTAGTGTTCAT
GAATTCCTTGTGGCATAATGGACTGTGCCAAAGTTCAAGGAAAGTCTTTCAAAATTTTCCAGTATATGGATGTGGGAGTT
GGTCTATATGTGTGCAAATCGAATTCCCGCGGCCGCCATGGCGGCCGGGAGCATGCGACGTCGGGCCCAATTCGCCCTAT
AGTGAGTCGTATTACAATTCACTGGCCGTCGTTTTACAACGTCGTGACTGGGAAAACCCTGGCGTTACCCAACTTAATCG
CCTTGCAGCACATCCCCCTTTCGCCAGCTGGCGTAATAGCGAAGAGGCCCGCACCGATCGCCCTTCCCAACAGTTGCGC
AGCCTGAATGGCGAATGGACGCGCCCTGTAGCGGCGCATTAAGCGCGGCGGGTGTGGTGGTTACGCGCARCGGTGACCCG
CTACAYTTGCCAGCGCCCTARSCGCCCGCTCCTTTTCSYTTTCTTTC
>P7.C4
TGARWKGGGCTTGGTKAATGAATTTGGGGCTGTCTTATTTAGGAAGTCCTATCAGATGATTATCTGGTTTGCAAGACGGA
TCACTTTTTATAGCTGATATTTTATATGTTTTAGCCTCCATTAGAACCTATGTTGTCTTATTTTGGTATTTTGTCATAAA
TTTGTATCATCGGATGTTATAAGTCAATTGCTTCTGAAAATAAGTCAAGGTATGACATACAGAACAAAGTCTGTTATGAA
ATAAATTTCCACTTACTTGATTAAGTTTTATACTTTCAGGTTTGGCTGGTTTGTCTACTGCAAAGTATTTGGCCGAGTGC
AGGTCATAAACCTATAGTGTTGGAAGCTAGGGATGTTCTGGGAGGAAAGGTAGCCAAATTATTACTCATTAGTGTTCATG
AATTCCTTGTGGCATAATGGACTGTGCCAAAGTTCAAGGAAAGTCTTTCAAAATTTTCCAGTATATGGATGTGGGAGTTG
GTCTATATGTGTGCAAATCGAATTCCCGCGGCCGCCATGGCGGCCGGGAGCATGCGACGTCGGGCCCAATTCGCCCTATA
GTGAGTCGTATTACAATTCACTGGCCGTCGTTTTACAACGTCGTGACTGGGAAAACCCTGGCGTTACCCAACTTAATCGC
CTTGCAGCACATCCCCCTTTCGCCAGCTGGCGTAATAGCGAAGAGGCCCGCACCGATCGCCCTTCCCAACAGTTGCGCAG
CCTGAATGGCGAATGGACGCGCCCTGTAGCGGCGCATTAAGCGCGGCGGGTGTGGTGGTTACGCGCAGCGTGACCGCTAC
ACTTGCCAGCGCCCTAGCGCCCGCTCCTTTCGCTTTCTTCCCTTCCTTTCTCGCACGTTCGCCGGCTTTCCCCGTCAGCT
CTAAATCGGGGGCTCCTTAGGGTTCCGAATTAGTGCCTTACGGCACCTCGACCCCAAAAAACTTGAATAAGGGKGAAKGG
GTTCMACG
>P7.C5
AGWKWWGSCTTGKGGATKGAATTTGGGGGCTGTCTTATTTAGGAGTCCWATCAGATGATTATCTGGTTTGCAAGACGGAT
CMCTTTTTATAGCTGATATTTTATATGTTTTAGCCTCCATTAGAACCTATGTTGTCTTATTTTGGTATTTTGTCATAAAT
TTGTATCATCGGATGTTATAAGTCAATTGCTTCTGAAAATAAGTCAAGGTATGACATACAGAACAAAGTCTGTTATGAAA
TAAATTTCCACTTACTTGATTAAGTTTTATACTTTCAGGTTTGGCTGGTTTGTCTACTGCAAAGTATTTGGCCGAGTGCA
GGTCATAAACCTATAGTGTTGGAAGCTAGGGATGTTCTGGGAGGAAAGGTAGCCAAATTATTACTCATTAGTGTTCATGA
ATTCCTTGTGGCATAATGGACTGTGCCAAAGTTCAAGGAAAGTCTTTCAAAATTTTCCAGTATATGGATGTGGGAGTTGG
TCTATATGTGTGCAAATCGAATTCCCGCGGCCGCCATGGCGGCCGGGAGCATGCGACGTCGGGCCCAATTCGCCCTATAG
TGAGTCGTATTACAATTCACTGGCCGTCGTTTTACAACGTCGTGACTGGGAAAACCCTGGCGTTACCCAACTTAATCGCC
TTGCAGCACATCCCCCTTTCGCCAGCTGGCGTAATAGCGAAGAGGCCCGCACCGATCGCCCTTCCCAACAGTTGCGCAGC
CTGAATGGCGAATGGACGCGCCCTGTAGCGGCGCATTAAGCGCGGCGGGTGTGGTGGTTACGCGCAGCGTGACCGCTACA
CTTGCCAGCGCCCTAGCGCCCGCTCCTWTTCSCTTTCTTCCCTTCCTTTCTYCGMCASATCGCCGGCTTTCCCGTCAAGC
TCTAAATCGGGGGCTYCCTTTTAGGGTTYCCGATTAGGTGCTTTACGGGCACCTYCAACCCCCTAAAACTGTATAGGTTG
AATGTTCACGCATAGGTGGGCACACTGACGSACTMWG
>P10.C4
TGRRWWGGCCTGTGATGAATTTGGGGCTGTCTTATTTAGGAGTCCTATCAGATGATTATCTGGTTTGCAAGACGGATCAC
TTTTTATAGCTGATATTTTATATGTTTTAGCCTCCATTAGAACCTATGTTGTCTTATTTTGGTATTTTGTCATAAATTTG
TATCATCGGATGTTATAAGTCAATTGCTTCTGAAAATAAGTCAAGGTATGACATACAGAACAAAGTCTGTTATGAAATAA
ATTTCCACTTACTTGATTAAGTTTTATACTTTCAGGTTTGGCTGGTTTGTCTACTGCAAAGTATTTGGCTGCAGGTCATA
AACCTATAGTGTTGGAAGCTAGGGATGTTCTGGGAGGAAAGGTAGCCAAATTATTACTCATTAGTGTTCATGAATTCCTT
GTGGCATAATGGACTGTGTCAAAGTTCAAGGAAAGTCTTTCAAAATTTTCCAGTATATGGATGTGGGAGTTGGTCTATAT
GTGTGCAAATCGAATTCCCGCGGCCGCCATGGCGGCCGGGAGCATGCGACGTCGGGCCCAATTCGCCCTATAGTGAGTCG
TATTACAATTCACTGGCCGTCGTTTTACAACGTCGTGACTGGGAAAACCCTGGCGTTACCCAACTTAATCGCCTTGCAGC
ACATCCCCCTTTCGCCAGCTGGCGTAATAGCGAAGAGGCCCGCACCGATCGCCCTTCCCAACAGTTGCGCAGCCTGAATG
GCGAATGGACGCGCCCTGTAGCGGCGCATTAAGCGCGGCGGGTGTGGTGGTTACGCGCAGCGTGACCGCTACACTTGCCA
GCGCCCTAGCGCCGCTCCTTTCGCTTTCTTCCCTTTCCTTTCTCGCCACGTTCGCCGGGCTTTCCCGRTCAAGCTCTAAA
TCGGGGGGCTCCCTTTAAGGGTTCCCGATTAGTGCTTACGGGCACCTCGACCCAAAACCTTGGATTAAGGGGGKGATGAT
TTMCASGCTARGTTG
>P10.C5
GMGWRGWMYCCTGGGKGAWGGATTTGGGGCTGTCTTATTTAGGAGTCCTATCAGATGATTATCTGGTTTGCAAGACGGAT
CMCTTTTTATAGCTGATATTTTATATGTTTTAGCCTCCATTAGAACCTATGTTGTCTTATTTTGGTATTTTGTCATAAAT
TTGTATCATCGGATGTTATAAGTCAATTGCTTCTGAAAATAAGTCAAGGTATGACATACAGAACAAAGTCTGTTATGAAA
TAAATTTCCACTTACTTGATTAAGTTTTATACTTTCAGGTTTGGCTGGTTTGTCTACTGCAAAGTATTTGTTTGCAGGTC
ATAAACCTATAGTGTTGGAAGCTAGGGATGTTCTGGGAGGAAAGGTAGCCAAATTATTACTCATTAGTGTTCATGAATTC
CTTGTGGCATAATGGACTGTGCCAAAGTTCAAGGAAAGTCTTTCAAAATTTTCCAGTATATGGATGTGGGAGTTGGTCTA

TATGTGTGCAAATCGAATTCCCGCGGCCGCCATGGCGGCCGGGAGCATGCGACGTCGGGCCCAATTCGCCCTATAGTGAG
TCGTATTACAATTCACTGGCCGTCGTTTTACAACGTCGTGACTGGGAAAACCCTGGCGTTACCCAACTTAATCGCCTTG
CAGCACATCCCCCTTTCGCCAGCTGGCGTAAWAGCRAAGAGGCCCGCACCGATCGCCCTTCCCAACAGTTGCGCAGCCTG
AATGGCGAATGGACGCGCCCCTGTAGCGGGCGCATTAAGCGCGGCGGGTGTGGTGGTTACGCGCARSCGTGACCGCTACA
CTTGCMAGCGCCCTAGCGSCCGCTCCTTTCGCTTTCTTCCCTTCCTTTCYSSCMCGTTMGCCGGCTTTCCCCGTCAGCTC
WAATCGGGGGGCYCYCTTARGGGTTTCGATTRTGCCTTACCGCCMCCCTCGAACCCCTAAAACACTGGACTTACGGCGTA
AKGTGCTTYACAC
>P10.C6
CTTAATTTTTTTAATAATGGTTTTAARCCTCCATTAGAMMCCTWTGTTGTCTTATTTTGGTATTTTGTCATAAATTTGTA
TCATCGGATGTTATAAGTCAATTGCTTCTGAAAATAAGTCMAGGTATGACATACARAACAAAGTCTGTTATGAAATAAAT
TTCCACTTACTTGATTAAGTTTTATACTTTCAGGTTTGGCTGGTTTGTCTACTGCAAAGTATTTGGCTGCAGGTCATAAA
CCTATAGTGTTGGAAGCTAGGGATGTTCTGGGARGAAAGGTAGCCAAATTATTACTCATTAGTGTTCATGAATTCCTTGT
GGCATAATGGAYTGTGCCAAAGTTCAAGGAAAGYCTT
>P10.C7
CKRAKKKSSTKKKKGATKGAATTTGGGGCTGTCTTATTTAGGAGTCCTATCAGATGATTATCTGGTTTGCAAGACGGATC
ATTAAWWWATAGCTGATATTTTATATGTTTTAGCCTCCATTAGAACCTATGTTGTCTTATTTTGGTATTTTGTCATAAAT
TTGTATCATCGGATGTTATAAGTCAATTGCTTCTGAAAATAAGTCAAGGTATGACATACAGAACAAAGTCTGTTATGAAA
TAAATTTCCACTTACTTGATTAAGTTTTATACTTTCAGGTTTGGCTGGTTTGTCTACTGCAAAGTATTTGGCTGCAGGTC
ATAAACCTATAGTGTTGGAAGCTAGGGATGTTCTGGGAGGAAAGGTAGCCAAATTATTACTCATTAGTGTTCATGAATTC
CTTGTGGCATAATGGACTGTGTCAAAGTTCAAGGAAAGTCTTTCAAAATTTTCCAGTATATGGATGTGGGAGTTGGTCTA
TATGTGTGCAAATCGAATTCCCGCGGCCGCCATGGCGGCCGGGAGCATGCGACGTCGGGCCCAATTCGCCCTATAGTGAG
TCGTATTACAATTCACTGGCCGTCGTTTTACAACGTCGTGACTGGGAAAACCCTGGCGTTACCCAACTTAATCGCCTTGC
AGCACATCCCCCTTTCGCCAGCTGGCGTAATAKCGAAGAGGCCCCGCACCGATCGCCCTTTCCCAACAGTTGCCCAGCCT
GAATGGCAATGGACGCGCCCTGTAGCGGCGCAWTTAAGCGCGGCGGGTGGTGGTGGTTACGCGCAGCGTGACCGCTACAY
TTG
>P5.C1
TRGGGGGGSCTGTTYYTTWWTTTTTAGGGAGTCCTATCAGATGATTATCTGGTTTGCAAGACSSMTYWCTTTTTATAGCT
GATATTTTATATGTTTTAGCCTCCATYAGAACCTATGTTGTCTTATTTTGGTATTTTGTCATAAATTTGTATCATCGGAT
GTTATAAGTCAATTGCTTCTGAAAATAAGTCAAGGTATGACATACAGAACAAAGTCTGTTATGAAATAAATTTCCACTTA
CTTGATTAAGTTTTATACTTTCAGGGATGTTCTGGGAGGAAAGGTAGCCAAATTATTACTCATTAGTGTTCATGAATTCC
TTGTGGCATAATGGACTGTGTCAAAGTTCAAGGAAAGTCTTTCAAAATTTTCCAGTATATGGATGTGGGAGTTGGTCTAT
ATGTGTGCAAATCGAATTCCCGCGGCCGCCATGGCGGCCGGGAGCATGCGACGTCGGGCCCAATTCGCCCTATAGTGAGT
CGTATTACAATTCACTGGCCGTCGTTTTACAACGTCGTGACTGGGAAAACCCTGGCGTTACCCAACTTAATCGCCTTGCA
GCACATCCCCCTTTCGCCAGCTGGCGTAATAGCGAAGAGGCCCGCACCGATCGCCCTTCCCAACAGTTGCGCAGCCTGAA
TGGCGAATGGACGCRCCCTGTAGCGGCGCATTAAGCGCGGCGGGTGTGGTGGTTACGCGCAGCGTGACCGCTACACTTGT
CAGCGCCCTAGCGCCGCTCCTTTCRCTTWCTTCCCTTCCTTCTCGMCACRATCGCCGGCTTTCCCCGTCAGCTCYAAAMC
GGGGGCTCCCYTWAGGGTMCCSATTTAGTGCTTTACKGCCWCCTCGACCCCCAWAAAACCTTGATT
>P5.C2
GWKGSMTKGGKMAWTGAWTTTTGGGGCTGTCTTATTTAGGAGTCCTATCAGATGATTATCTGGTTTGCAAGACGGATCAC
TTTTTATAGCTGATATTTTATATGTTTTAGCCTCCATTAGAACCTATGTTGTCTTATTTTGGTATTTTGTCATAAATTTG
TATCATCGGATGTTATAAGTCAATTGCTTCTGAAAATAAGTCAAGGTATGACATACAGAACAAAGTCTGTTATGAAATAA
ATTTCCACTTACTTGATTAAGTTTTATACTTTCAGGGATGTTCTGGGAGGAAAGGTAGCCAAATTATTACTCATTAGTGT
TCATGAATTCCTTGTGGCATAATGGACTGTGTCAAAGTTCAAGGAAAGTCTTTCAAAATTTTCCAGTATATGGATGTGGG
AGTTGGTCTATATGTGTGCAAATCGAATTCCCGCGGCCGCCATGGCGGCCGGGAGCATGCGACGTCGGGCCCAATTCGCC
CTATAGTGAGTCGTATTACAATTCACTGGCCGTCGTTTTACAACGTCGTGACTGGGAAAACCCTGGCGTTACCCAACTTA
ATCGCCTTGCAGCACATCCCCCTTTCGCCAGCTGGCGTAATAGCGAAGAGGCCCGCACCGATCGCCCTTCCCAACAGTTG
CGCAGCCTGAATGGCGAATGGACGCGCCCTGTAGCGGCGCATTAAGCGCGGCGGGTGTGGTGGTTACGCGCAGCGTGACC
GCTACACTTGCCAGCGCCCTAGCGCCCGCTCCTTTCGCTTTCTTCCCTTCCTTTCTCGCCACGTTCGCCGGCTTTCCCCG
TCAAGCTCTAATCGGGGGCTCCCTTTAGGGTTCCGATTAGTGCTTACGGCACCTCGAMCCCAAAAACTTGATAGGTGATG
GTTCACGTAGTGGGCCATCGCCCTGATAGACGGCTTTCGCCCTTGGACGTTGGARGTCCMSGATTCCTTTAATWRGTGGG
GAACT
>P5.C4
GMCYTTTGRTTCMTTTATYTTTAGGGAAGTCCTATCAGATGATTATCTGGTTTGCAAGACGGCATCWYTTTTTATAGCTG
ATATTTTATATGTTTTAGCCTCCATTMKAACCTATGTTGTCTTATTTTGGTATTTTGTCATAAATTTGTATCATCGGATG
TTATAAGTCAATTGCTTCTGAAAATAAGTCAAGGTATGACATACAGAACAAAGTCTGTTATGAAATAAATTTCCACTTAC
TTGATTAAGTTTTATACTTTCAGGGATGTTCTGGGAGGAAAGGTAGCCAAATTATTACTCATTAGTGTTCATGAATTCCT
TGTGGCATAATGGACTGTGTCAAAGTTCAAGGAAAGTCTTTCAAAATTTTCCAGTATATGGATGTGGGAGTTGGTCTATA

TGTGTGCAAATCGAATTCCCGCGGCCGCCATGGCGGCCGGGAGCATGCGACSTCGGGCCCAATTCGCCCTATAGTGAGTC
GTATTACAATTCACTGGCCGTCGTTTTACAACSYCGTGACTGGGAAAACCCTGGCGTTACCCAACTTAATCGMCTTGCAR
ACATCCCCCTTTCGCCAGCTGGCGTAATAGCGAAGAGGCCCGCACCGATCGCCCTTCCCAACAGTTGCGCASCCTGAATG
G
>WT1.C9
GKGCTGTATGATTGGGTGCTATTAGGATCTATCAAGATTCTGGTTGAACGTCCTTTTTAACTGTTTTTTTGTTTACCCCT
TAAACCATGTGCTTATTTTGTATTTTGTCATAAATTTKATCATCGGATGTTATAAGTCAATTGCTTCTGAAAATAATCAA
GGTATGACATACAAACAAATCTGTATGAATAAATTTCCCTTACTTGATAATTTTATACTTTCAGTTGGTGGTTGCTACTG
CAATATTGGCATGCAGCTAAACCTATATGTGAACTAGGGTGTCTGGAGAAAGGACCATTATATCTTATGTCTGATTCTTG
GCTATGATGGCAATTCAGAAGCTTAATTTCATAATGGTGGGATTGTCAATTGGAACAATCCCGCCCCTGCGCGACTCACT
CGCCATCCCAATGTCTTTCATATGGCTCTTAACTCTGCGGAACCGCTACACTACCCTGACCTCCTTCCACGCTAACAAAG
CGACACCCTCCAATCCCTGTGCATGCCCTGACCCTACCCGTGGTGACACKGCCAATGACCTACCCCTCCTCCTCTCCCCT
CCGTCKACCACGCCTAGCATTGTCGCCCCAATGAGAGCTTGCCCAAGTCCTATACTTATATTCYGACCTCKTTTAGTCTG
TTAGAAAAAAAATTRTCMGTCMGTGGCTTAACAATATCTTCATGTCAGTGCGTCGYRG
>WT1.C2
GRRKGCTKKKGKWAATGATTTGGGGCTGTCTTATTTAGGARTCCTAATCAGATGATTATCTGGTTTGCAAGACGGATCAC
TTTTTATAGCTGATATTTTATATGTTTTAGCCTCCATTAGAACCTATGTTGTCTTATTTTGGTATTTTGTCATAAATTTG
TATCATCGGATGTTATAAGTCAATTGCTTCTGAAAATAAGTCAAGGTATGACATACAGAACAAAGTCTGTTATGAAATAA
ATTTCCACTTACTTGATTAAGTTTTATACTTTCAGGTTTGGCTGGTTTGTCTACTGCAAAGTATTTGGCCGATGCAGGTC
ATAAACCTATAGTGTTGGAAGCTAGGGATGTTCTGGGAGGAAAGGTAGCCAAATTATTACTCATTAGTGTTCATGAATTC
CTTGTGGCATAATGGACTGTGTCAAAGTTCAAGGAAAGTCTTTCAAAATTTTCCAGTATATGGATGTGGGAGTTGGTCTA
TATGTGTGCAAATCGAATTCCCGCGGCCGCCATGGCGGCCGGGAGCATGCGACGTCGGGCCCAATTCGCCCTATAGTGAG
TCGTATTACAATTCACTGGCCGTCGTTTTACAACGTCGTGACTGGGAAAACCCTGGCGTTACCCAACTTAATCGCCTTGC
AGCACATCCCCCTTTCGCCAGCTGGCGTAATAGCGAAGAGGCCCGCACCGATCGCCCTTCCCAACAGTTGCGCAGCCTGA
ATGGCGAATGGACGCGCCCTGTAGCGGCGCATTAAGCGCGGCGGGTGTGGTGGTTACGCGCAGCGTGACCGCTACACTTG
CCAGCGCCCTAGCGCCCGCTCCTTTCGCTTTCTTCCCTTCCTTTCTCGCACGTTCGCCGGCTTTCCCGTCAAGCTCTAAT
CGGGGCTCCTTAGGGTTCCGATTTAGTGCTTACCGCACCTCGACCCCCAAAAACTTGATTTAGGGGGTGAATGGGTTCTM
MRCGGTTAG
>WT1.C3
TRKKGGSCTKKKKKRAWTGAATTTGGGGGCTGTCTTATTTAGGAGTCCTATCAGATGATTATCTGGTTTGCAAGACGGAT
CACTTTTTATAGCTGATATTTTATATGTTTTAGCCTCCATTAGAACCTATGTTGTCTTATTTTGGTATTTTGTCATAAAT
TTGTATCATCGGATGTTATAAGTCAATTGCTTCTGAAAATAAGTCAAGGTATGACATACAGAACAAAGTCTGTTATGAAA
TAAATTTCCACTTACTTGATTAAGTTTTATACTTTCAGGTTTGGCTGGTTTGTCTACTGCAAAGTATTTGGCCGATGCAG
GTCATAAACCTATAGTGTTGGAAGCTAGGGATGTTCTGGGAGGAAAGGTAGCCAAATTATTACTCATTAGTGTTCATGAA
TTCCTTGTGGCATAATGGACTGTGTCAAAGTTCAAGGAAAGTCTTTCAAAATTTTCCAGTATATGGATGTGGGAGTTGGT
CTATATGTGTGCAAATCGAATTCCCGCGGCCGCCATGGCGGCCGGGAGCATGCGACGTCGGGCCCAATTCGCCCTATAGT
GAGTCGTATTACAATTCACTGGCCGTCGTTTTACAACGTCGTGACTGGGAAAACCCTGGCGTTACCCAACTTAATCGCCT
TGCAGCACATCCCCCTTTCGCCAGCTGGCGTAATAGCGAAGAGGCCCGCACCGATCGCCCTTCCCAACAGTTGCGCAGCC
TGAATGGCGAATGGACGCGCCCTGTAGCGGCGCATTAAGCGCGGCGGGTGTGGTGGTTACGCGCAGCGTGACCGCTACAC
TTGCCAGCGCCCTAGCGCCCGCTCCTTTCGCTTTCTTCCCTTTCCTTTCTCGCACGTTCGCCGGCTTTCCCCGTCAGCTC
TAAATCGGGGGGCTCCCTTTAAGGTTTCGATTTAGTGCCTTWCGCACTCGACCCCAAAAACTTGATTAGGGGGTGAGAKG
TGGTTTACCAG
>WT1.C4
CTCCMWYTTWATTTTWAGGGARGTCCCTATCAGATGATTATCTGGTTTGCAAGACGGATCACTTTTTATAGCTGATATTT
TATATGTTTTAGCCTCCATTAGAACCTATGTTGTCTTATTTTGGTATTTTGTCATAAATTTGTATCATCGGATGTTATAA
GTCAATTGCTTCTGAAAATAAGTCAAGGTATGACATACAGAACAAAGTCTGTTATGAAATAAATTTCCACTTACTTGATT
AAGTTTTATACTTTCAGGTTTGGCTGGTTTGTCTACTGCAAAGTATTTGGCCGATGCAGGTCATAAACCTATAGTGTTGG
AAGCTAGGGATGTTCTGGGAGGAAAGGTAGCCAAATTATTACTCATTAGTGTTCATGAATTCCTTGTGGCATAATGGACT
GTGTCAAAGTTCAAGGAAAGTCTTTCAAAATTTTCCAGTATATGGATGTGGGAGTTGGTCTATATGTGTGCAAATCGAAT
TCCCGCGGCCGCCATGGCGGCCGGGAGCATGCGACGTCGGGCCCAATTCGCCCTATAGTGAGTCGTATTACAATTCACTG
GCCGTCGTTTTACAACGTCGTGACTGGGAAAACCCTGGCGTTACCCAACTTAATCGCCTTGCAGCACATCCCCCTTTCGC
CAGCTGGCGTAATAGCGAAGAGGCCCGCACCGATCGCCCTTCCCAACAGTTGCGCAGCCTGAATGGCGAATGGACGCGCC
CTGTAGCGGCGCATTAAGCGCGGCSGGGTGTGGTGGTTACGCGCAGCGTGACCGCTACACTTGCCAGCGCCCTAGCRGCC
GCTCCTTTCGCTTCTTCCCTTCCTTTCTCGCMACGTTCGCCGGCTTTCCCCGTCAGCTCTAATCGGGGSCTCCCCTTAGG
GTCCGAATTAGTGCTTACGGCAMCMTCGAMCCCAAAACTTGAATAGGGKRATGATCACGGTRRCTGSGCCATYGCCCCKG
TAAMRGCA
>WT2.C1

GGSMTTKGKRAWKAWTTTGGGGCTGTCTTATTTAGGAGTCCTATCAGATGATTATCTGGTTTGCAAGACGGATCACTTTT
TATARCTGATATTTTATATGTTTTAGCCTCCATTASAACCTATGTTGTCTTATTTTGGTATTTTGTCATAAATTTGTATC
ATCGGATGTTATAAGTCAATTGCTTCTGAAAATAAGTCAAGGTATGACATACASAACAAAGTCTGTTATGAAATAAATTT
CCACTTACTTGATTAAGTTTTATACTTTCAGGTTTGGCTGGTTTGTCTACTGCAAAGTATTTGGCCGATGCAGGTCATAA
MCCTATAGTGTTGGAAGCTAGGGATGTTCTGGGAGGAAAGGTAGCCAAATTATTACTCATTAGTGTTCATGAATTCCTTG
TGGCATAATGGACTGTGTCAAASTTCAAGGAAAGTCTTTCAAAATTTTCCAGKATATGGATGTGGGAGTTGGTCTATATG
TGTGCAAATCCMATTCCCGCGGCCGCCATGGCRGCCGG
>WT2.C5
TGAWKGCTGKGATGATTTGGGGCTGTCTTATTTAGGAGTCCTATCAGATGATTATCTGGTTTGCAAGACGGATCACTTTT
TATAGCTGATATTTTATATGTTTTAGCCTCCATTAGAACCTATGTTGTCTTATTTTGGTATTTTGTCATAAATTTGTATC
ATCGGATGTTATAAGTCAATTGCTTCTGAAAATAAGTCAAGGTATGACATACAGAACAAAGTCTGTTATGAAATAAATTT
CCACTTACTTGATTAAGTTTTATACTTTCAGGTTTGGCTGGTTTGTCTACTGCAAAGTATTTGGCCGATGCAGGTCATAA
ACCTATAGTGTTGGAAGCTAGGGATGTTCTGGGAGGAAAGGTAGCCAAATTATTACTCATTAGTGTTCATGAATTCCTTG
TGGCATAATGGACTGTGTCAAAGTTCAAGGAAAGTCTTTCAAAATTTTCCAGTATATGGATGTGGGAGTTGGTCTATATG
TGTGCAAATCGAATTCCCGCGGCCGCCATGGCGGCCGGGAGCATGCGACGTCGGGCCCAATTCGCCCTATAGTGAGTCGT
ATTACAATTCACTGGCCGTCGTTTTACAACGTCGTGACTGGGAAAACCCTGGCGTTACCCAACTTAATCGCCTTGCAGCA
CATCCCCCTTTCGCCAGCTGGCGTAATAGCGAAGAGGCCCGCACCGATCGCCCTTCCCAACAGTTGCGCAGCCTGAATGG
CGAATGGACGCGCCCTGTAGCGGCGCATTAAGCGCGGCGGGTGTGGTGGTTACGCGCAGCGTGACCGCTACACTTGCCAG
CGCCCTAGCGCCCGCTCCTTTCGCTTTCTTCCCTTCCTTTCTCGCACGTTCGCCGGCTTTCCCCGTCAAGCTCTAATCGG
GGGCTCCTTAGGGTTCGATTTAGTGCTTTACCGCACTCGACCCAAAAACTTGATAGGGTATGGTTCMGGTTATGGCCATS
ACCCKTGTAWWTGAGARCACG
>WT2.C8
AMMMMWMGSRTRKCKGRAAKGRAWTTTGGGGGCTGTCTTATTTAGGAGTCCTATCAGATGATTATCTGGTTTGCAAGACG
GATCACTTTTTATAGCTGATATTTTATATGTTTTAGCCTCCATTAGAACCTATGTTGTCTTATTTTGGTATTTTGTCATA
AATTTGTATCATCGGATGTTATAAGTCAATTGCTTCTGAAAATAAGTCAAGGTATGACATACAGAACAAAGTCTGTTATG
AAATAAATTTCCACTTACTTGATTAAGTTTTATACTTTCAGGTTTGGCTGGTTTGTCTACTGCAAAGTATTTGGCCGATG
CAGGTCATAAACCTATAGTGTTGGAAGCTAGGGATGTTCTGGGAGGAAAGGTAGCCAAATTATTACTCATTAGTGTTCAT
GAATTCCTTGTGGCATAATGGACTGTGTCAAAGTTCAAGGAAAGTCTTTCAAAATTTTCCAGTATATGGATGTGGGAGTT
GGTCTATATGTGTGCAAATCGAATTCCCGCGGCCGCCATGGCGGCCGGGAGCATGCGACGTCGGGCCCAATTCGCCCTAT
AGTGAGTCGTATTACAATTCACTGGCCGTCGTTTTACAACGTCGTGACTGGGAAAACCCTGGCGTTACCCAACTTAATCG
CCTTGCAGCACATCCCCCTTTCGCCAGCTGGCGTAATAGCGAAGAGGCCCGCACCGATCGCCCTTYCCCAACAGTTGCGC
AGCCTGAATGGCGAATGGACGCGCCCCTGTAGCGGCGCATTAAGCGCGGCGGGTGTGGTGGTTACGCGCARCGTGACCGC
TACACTTGMCAGCGCCCTAGCSCCGCTCCTTTCGCTTTCTTCCCTTCCTTCTCGCACGTTCGCCGGCTTTCCCCGTCARY
TCTAATCGGGGGCTCCCTTTAGGTTCCGATTTAGTGCTTACCGCACCTCGACCCTAAAACTTGATAARGGGGWATGGCTT
CWACRMKAGCC
>WT2.C9
TRATGGCCTGTGATGATTTGGGGCTGTCTTATTTAGGAGTCCTATCAGATGATTATCTGGTTTGCAAGACGGATCACTTT
TTATAGCTGATATTTTATATGTTTTAGCCTCCATTAGAACCTATGTTGTCTTATTTTGGTATTTTGTCATAAATTTGTAT
CATCGGATGTTATAAGTCAATTGCTTCTGAAAATAAGTCAAGGTATGACATACAGAACAAAGTCTGTTATGAAATAAATT
TCCACTTACTTGATTAAGTTTTATACTTTCAGGTTTGGCTGGTTTGTCTACTGCAAAGTATTTGGCCGATGCAGGTCATA
AACCTATAGTGTTGGAAGCTAGGGATGTTCTGGGAGGAAAGGTAGCCAAATTATTACTCATTAGTGTTCATGAATTCCTT
GTGGCATAATGGACTGTGTCAAAGTTCAAGGAAAGTCTTTCAAAATTTTCCAGTATATGGATGTGGGAGTTGGTCTATAT
GTGTGCAAATCGAATTCCCGCGGCCGCCATGGCGGCCGGGAGCATGCGACGTCGGGCCCAATTCGCCCTATAGTGAGTCG
TATTACAATTCACTGGCCGTCGTTTTACAACGTCGTGACTGGGAAAACCCTGGCGTTACCCAACTTAATCGCCTTGCAGC
ACATCCCCCTTTCGCCAGCTGGCGTAATAGCGAAGAGGCCCGCACCGATCGCCCTTCCCAACAGTTGCGCAGCCTGAATG
GCGAATGGACGCGCCCTGTAGCGGCGCATTAAGCGCGGCGGGTGTGGTGGTTACGCGCAGCGTGACCGCTACACTTGCCA
GCGCCCTAGCGCCCGCTCCTTTCGCTTTCTTCCCTTCCTTTCTCGCACGTTCGCCGGCTTTCCCCGTCAAGCTCTAATCG
GGGGGCTCCCTTTAGGGTTCCGATTTAGTGCTTTACGGCACTCGACCCCAAAAAACTGATTAGGGTGAATGGATTCACSG
TWGAGGKGGGG
>G1.C4
TAKGGCTTKTGATGAATTTGGGGCTGTCTTATTTAGGAGTCCTATCAGATGATTATCTGGTTTGCAAGACGGATCACTTT
TTATAGCTGATATTTTATATGTTTTAGCCTCCATTASAACCTATGTTGTCTTATTTTGGTATTTTGTCATAAATTTGTAT
CATCGGATGTTATAAGTCAATTGCTTCTGAAAMYAAGTCAAGGTATGACATACAGAACAAAGTCTGTTATGAAATAAATT
TCCACTTACTTGATTAAGTTTTATACTTTCAGGTTTGGCTGGTTTGTCTACTGCAAAGTATTTGGCCGATGCAGGTCATA
AACCTATAGTGTTGGAAGCTAGGGATGTTCTGGGAGGAAAGGTAGCCAAATTATTATTCATTAKTGTTCACGAATTCC
>G1.C5
CGKKGCTKGTGATGAATTTGGGGCTGTCTTATTTAGGAGTCCTATCAGATGATTATCTGGTTTGCAAGACGGATCACTTT

TTATAGCTGATATTTTATATGTTTTAGCCTCCATTAGAACCTATGTTGTCTTATTTTGGTATTTTGTCATAAATTTGTAT
CATCGGATGTTATAAGTCAATTGCTTCTGAAAATAAGTCAAGGTATGACATACAGAACAAAGTCTGTTATGAAATAAATT
TCCACTTACTTGATTAAGTTTTATACTTTCAGGTTTGGCTGGTTTGTCTACTGCAAAGTATTTGGCCGATGCAGGTCATA
AACCTATAGTGTTGGAAGCTAGGGATGTTCTGGGAGGAAAGGTAGCCAAATTATTACTCATTAGTGTTCATGAATTCCTT
GTGGCATAATGGACTGTGCCAAAGTTCAAGGAAAGTCTTTCAAAATTTTCCAGTATATGGATGTGGGAGTTGGTCTATAT
GTGTGCAAATCGAATTCCCGCGGCCGCCATGGCGGCCGGGAGCATGCGACGTCGGGCCCAATTCGCCCTATAGTGAGTCG
TATTACAATTCACTGGCCGTCGTTTTACAACGTCGTGACTGGGAAAACCCTGGCGTTACCCAACTTAATCGCCTTGCAGC
ACATCCCCCTTTCGCCAGCTGGCGTAATAGCGAAGAGGCCCGCACCGATCGCCCTTCCCAACAGTTGCGCAGCCTGAATG
GCGAATGGACGCGCCCTGTAGCGGCGCATTAAGCGCGGCGGGTGTGGTGGTTACGCGCAGCGTGACCGCTACACTTGCCA
GCGCCCTAGCGCCCGCTCCTTTCGCTTTCTTCCCTTTCCTTTCTCGCMACGTTCGCCGGCTTTCCCCGTCAAGCTCTAAW
CGGGGGGCTCCCTTTAGGTTCCKATTTRTGCTTACCGCACTCGAACCCCAAAACTTGATTAGGGGTGATKGGTTMCASSA
TRCTGCGC
>G1.C7
TRWKGCCTTGTGAATGAATTTGGGGCTGTCTTATTTAGGAGTCCTATCAGATGATTATCTGGTTTGCAAGACGGATCACT
TTTTATAGCTGATATTTTATATGTTTTAGCCTCCATTAGAACCTATGTTGTCTTATTTTGGTATTTTGTCATAAATTTGT
ATCATCGGATGTTATAAGTCAATTGCTTCTGAAAATAAGTCAAGGTATGACATACAGAACAAAGTCTGTTATGAAATAAA
TTTCCACTTACTTGATTAAGTTTTATACTTTCAGGTTTGGCTGGTTTGTCTACTGCAAAGTATTTGGCCGATGCAGGTCA
TAAACCTATAGTGTTGGAAGCTAGGGATGTTCTGGGAGGAAAGGTAGCCAAATTATTACTCATTAGTGTTCATGAATTCC
TTGTGGCATAATGGACTGTGTCAAAGTTCAAGGAAAGTCTTTCAAAATTTTCCAGTATATGGATGTGGGAGTTGGTCTAT
ATGTGTGCAAATCGAATTCCCGCGGCCGCCATGGCGGCCGGGAGCATGCGACGTCGGGCCCAATTCGCCCTATAGTGAGT
CGTATTACAATTCACTGGCCGTCGTTTTACAACGTCGTGACTGGGAAAACCCTGGCGTTACCCAACTTAATCGCCTTGCA
GCACATCCCCCTTTCGCCAGCTGGCGTAATAGCGAAGAGGCCCGCACCGATCGCCCTTCCCAACAGTTGCGCAGCCTGAA
TGGCGAATGGACGCGCCCTGTAGCGGCGCATTAAGCGCGGCGGGTGTGGTGGTTACGCGCAGCGTGACCGCTACACTTGC
CAGCGCCCTAGCGCCCGCTCCTTTCGCTTTCTTCCCTTCCTTTCTCGCCACGTTCGCCGGCTTTCCCCGTCAAGCTCTAA
TCGGGGGGCTCCCTTTAGGGTTCGATTTAGTGCTTACGGCACCTCGACCCCAAAAACTGATAGGTGATGTTCACGTTAGT
GGGCCCATTGCGCCCCCTGTGAATTRTG
>G1.C9
TCKKGCCTKAKGAATGATTTGGGGCTGTCTTATTTAGGAGTCCTATCAGATGATTATCTGGTTTGCAAGACGGATCACTT
TTTATAGCTGATATTTTATATGTTTTAGCCTCCATTAGAACCTATGTTGTCTTATTTTGGTATTTTGTCATAAATTTGTA
TCATCGGATGTTATAAGTCAATTGCTTCTGAAAATAAGTCAAGGTATGACATACAGAACAAAGTCTGTTATGAAATAAAT
TTCCACTTACTTGATTAAGTTTTATACTTTCAGGTTTGGCTGGTTTGTCTACTGCAAAGTATTTGGCCGATGCAGGTCAT
AAACCTATAGTGTTGGAAGCTAGGGATGTTCTGGGAGGAAAGGTAGCCAAATTATTACTCATTAGTGTTCATGAATTCCT
TGTGGCATAATGGACTGTGTCAAAGTTCAAGGAAAGTCTTTCAAAATTTTCCAGTATATGGATGTGGGAGTTGGTCTATA
TGTGTGCAAATCGAATTCCCGCGGCCGCCATGGCGGCCGGGAGCATGCGACGTCGGGCCCAATTCGCCCTATAGTGAGTC
GTATTACAATTCACTGGCCGTCGTTTTACAACGTCGTGACTGGGAAAACCCTGGCGTTACCCAACTTAATCGCCTTGCAG
CACATCCCCCTTTCGCCAGCTGGCGTAATAGCGAAGAGGCCCGCACCGATCGCCCTTCCCAACAGTTGCGCAGCCTGAAT
GGCGAATGGACGCGCCCTGTAGCGGCGCATTAAGCGCGGCGGGTGTGGTGGTTACGCGCAGCGTGACCGCTACACTTGCC
AGCGCCCTAGCGCCCGCTCCTTTCGCTTTCTTCCCTTCCTTTCTCGCACGTTCGCCGGCTTTCCCCGTCAAGCTCTAATC
GGGGGGCTCCTTTAGGTCGATTAGTGCTTACCGCACTCGAACCCCAAAACTGATAGGTATGTCACGGTACTGGCCCATSG
CCCGTKGTATKTGTRC
>G2.C1
GRKGGCTKGTGAATGATTTGGGGCTGTCTTATTTAGGAGTCCTATCAGATGATTATCTGGTTTGCAAGACGGATCACTTT
TTATAGCTGATATTTTATATGTTTTAGCCTCCATTAGAACCTATGTTGTCTTATTTTGGTATTTTGTCATAAATTTGTAT
CATCGGATGTTATAAGTCAATTGCTTCTGAAAATAAGTCAAGGTATGACATACAGAACAAAGTCTGTTATGAAATAAATT
TCCACTTACTTGATTAAGTTTTATACTTTCAGGTTTGGCTGGTTTGTCTACTGCAAAGTATTTGGCCGATGCAGGTCATA
AACCTATAGTGTTGGAAGCTAGGGATGTTCTGGGAGGAAAGGTAGCCAAATTATTACTCATTAGTGTTCATGAATTCCTT
GTGGCATAATGGACTGTGCCAAAGTTCAAGGAAAGTCTTTCAAAATTTTCCAGTATATGGATGAATCGAATTCCCGCGGC
CGCCATGGCGGCCGGGAGCATGCGACGTCGGGCCCAATTCGCCCTATAGTGAGTCGTATTACAATTCACTGGCCGTCGTT
TTACAACGTCGTGACTGGGAAAACCCTGGCGTTACCCAACTTAATCGCCTTGCAGCACATCCCCCTTTCGCCAGCTGGCG
TAATAGCGAAGAGGCCCGCACCGATCGCCCTTCCCAACAGTTGCGCAGCCTGAATGGCGAATGGACGCGCCCTGTAGCGG
CGCATTAAGCGCGGCGGGTGTGGTGGTTACGCGCAGCGTGMCCGCTACACTTGCCAGCGCCCTAGCGCCCGCTCCTTTCG
CTTTCTTCCCTTCCTTTCYSGCCACGTTCGCCGGCTTTCCCGTCAAGCTCTAAATCGGGGGCTCCTTTARGGTYCGATTA
ATGCTTACGGCAMCCTCGACCCCAAAAAAACATGATAAGGTGAATGGTTCCASGTAKYGGACATGCCTGKATGACCGGCT
TCTCGCCCTYGTGCARCGCTGKRGAGAACT
>G2.C4
AKRRKTGGCCTKTKAATGAATTTGGGGCTGTCTTATTTAGGAGTCCTATCAGATGATTATCTGGTTTGCAAGACSGATCA
CTTTTTATAGCTGATATTTTATATGTTTTAGCCTCCATTAGAACCTATGTTGTCTTATTTTGGTATTTTGTCATAAATTT

GTATCATCGGATGTTATAAGTCAATTGCTTCTGAAAATAAGTCAAGGTATGACATACAGAACAAAGTCTGTTATGAAATA
AATTTCCACTTACTTGATTAAGTTTTATACTTTCAGGTTTGGCTGGTTTGTCTACTGCAAAGTATTTGGCCGATGCAGGT
CATAAACCTATAGTGTTGGAAGCTAGGGATGTTCTGGGAGGAAAGGTAGCCAAATTATTACTCATTAGTGTTCATGAATT
CCTTGTGGCATAATGGACTGTGTCAAAGTTCAAGGAAAGTCTTTCAAAATTTTCCAGTATATGGATGTGGGAGTTGGTCT
ATATGTGTGCAAATCACTAGTGAATTCGCGGCCGCCTGCAGGTCGACCATATGGGAGAGCTCCCAACGCGTTGGATGCAT
AGCTTGAGTATTCTATAGTGTCACCTAAATAGCTTGGCGTAATCATGGTCATAGCTGTTTCCTGTGTGAAATTGTTATCC
GCTCACAATTCCACACAACATACGAGCCGGAAGCATAAAGTGTAAAGCCTGGGGTGCCTAATGAGTGAGCTAACTCACAT
TAATTGCGTTGCGCTCACTGCCCGCTTTCCAGTCGGGAAACCTGTCGTGCCAGCTGCATTAATGAATCGGCCAACGCGCG
GGGAGAGGCGGTTTGCGTATTGGGCGCTCTTCCGCTTCCTCGCTCACTGACTCSCTGCGCTSGGTCGTTCGGCTGCGCGA
GCGGTATCAGCTCMMTYCAAGGCGGTAWWMCGTTATTCCACMGATCAGGGTATAACGCAGGAGAACATGTTGAGCAAGTC
AGTCAAGGGCTMGGGATACCAGCSKATCG
>G2.C5
TGGARWGCCTTGTGATGATTTGGGGCTGTCTTATTTAGGAGTCCTATCAGATGATTATCTGGTTTGCAAGACGGATCACT
TTTTATAGCTGATATTTTATATGTTTTAGCCTCCATTAGAACCTATGTTGTCTTATTTTGGTATTTTGTCATAAATTTGT
ATCATCGGATGTTATAAGTCAATTGCTTCTGAAAATAAGTCAAGGTATGACATACAGAACAAAGTCTGTTATGAAATAAA
TTTCCACTTACTTGATTAAGTTTTATACTTTCAGGTTTGGCTGGTTTGTCTACTGCAAAGTATTTGGCCGATGCAGGTCA
TAAACCTATAGTGTTGGAAGCTAGGGATGTTCTGGGAGGAAAGGTAGCCAAATTATTACTCATTAGTGTTCATGAATTCC
TTGTGGCATAATGGACTGTGCCAAAGTTCAAGGAAAGTCTTTCAAAATTTTCCAGTATATGGATGTGGGAGTTGGTCTAT
ATGTGTGCAAATCGAATTCCCGCGGCCGCCATGGCGGCCGGGAGCATGCGACGTCGGGCCCAATTCGCCCTATAGTGAGT
CGTATTACAATTCACTGGCCGTCGTTTTACAACGTCGTGACTGGGAAAACCCTGGCGTTACCCAACTTAATCGCCTTGCA
GCACATCCCCCTTTCGCCAGCTGGCGTAATAGCGAAGAGGCCCGCACCGATCGCCCTTCCCAACAGTTGCGCAGCCTGAA
TGGCGAATGGACGCGCCCTGTAGCGGCGCATTAAGCGCGGCGGGTGTGGTGGTTACGCGCAGCGTGACCGCTACACTTGC
CAGCGCCCTAGCGCCCGCTCCTTTCGCTTTCTCCCTTCCTTTCTCGCACGTCSCCGGCTTTCCCCGTCAAGCTCTAATCG
GGGGCCTCCCTTTAAGGGTTCCGATTARGKGCTTACGCACCTCGACCCCAAAMCTKGATAGGCGTATGTTCMGTATGGTC
MMTTSGCCCTTGATGATATAGCACACATCG
>G2.C8
TCAKGGCCTTGTGATGAATTTGGGGCTGTCTTATTTAGGAGTCCTATCAGATGATTATCTGGTTTGCAAGACGGATCACT
TTTTATAGCTGATATTTTATATGTTTTAGCCTCCATTAGAACCTATGTTGTCTTATTTTGGTATTTTGTCATAAATTTGT
ATCATCGGATGTTATAAGTCAATTGCTTCTGAAAATAAGTCAAGGTATGACATACAGAACAAAGTCTGTTATGAAATAAA
TTTCCACTTACTTGATTAAGTTTTATACTTTCAGGTTTGGCTGGTTTGTCTACTGCAAAGTATTTGGCCGATGCAGGTCA
TAAACCTATAGTGTTGGAAGCTAGGGATGTTCTGGGAGGAAAGGTAGCCAAATTATTACTCATTAGTGTTCATGAATTCC
TTGTGGCATAATGGACTGTGCCAAAGTTCAAGGAAAGTCTTTCAAAATTTTCCAGTATATGGATGAATCGAATTCCCGCG
GCCGCCATGGCGGCCGGGAGCATGCGACGTCGGGCCCAATTCGCCCTATAGTGAGTCGTATTACAATTCACTGGCCGTCG
TTTTACAACGTCGTGACTGGGAAAACCCTGGCGTTACCCAACTTAATCGCCTTGCAGCACATCCCCCTTTCGCCAGCTGG
CGTAATAGCGAAGAGGCCCGCACCGATCGCCCTTCCCAACAGTTGCGCAGCCTGAATGGCGAATGGACGCGCCCTGTAGC
GGCGCATTAAGCGCGGCGGGTGTGGTGGTTACGCGCAGCGTGACCGCTACACTTGCCAGCGCCCTAGCGCCCGCTCCTTT
CGCTTTCTTCCCTTCCTTTCTCGCCACGTTCGCCGGCTTTCCCGTCAGCTCTAAATCGGGGGCTCCCTTTAGGGTTCGAT
TAGTGCTTACGGCACTCGACCCAAAAACTTGATAGGTGAATGGTTCACGTATGGCATCGACTGATGAACGGTTTTTYGGC
GCCCCCCTCTTGTG
>G3.C2
TGRKKGGCTGTKGATGAATTTGGGGCTGTCTTATTTAGGAGTCCTATCAGATGATTATCTGGTTTGCAAGACGGATCACT
TTTTATAGCTGATATTTTATATGTTTTAGCCTCCATTAGAACCTATGTTGTCTTATTTTGGTATTTTGTCATAAATTTGT
ATCATCGGATGTTATAAGTCAATTGCTTCTGAAAATAAGTCAAGGTATGACATACAGAACAAAGTCTGTTATGAAATAAA
TTTCCACTTACTTGATTAAGTTTTATACTTTCAGGTTTGGCTGGTTTGTCTACTGCAAAGTATTTGGCCGATGCAGGTCA
TAAACCTATAGTGTTGGAAGCTAGGGATGTTCTGGGAGGAAAGGTAGCCAAATTATTACTCATTAGTGTTCATGAATTCC
TTGTGGCATAATGGACTGTGTCAAAGTTCAAGGAAAGTCTTTCAAAATTTTCCAGTATATGGATGTGGGAGTTGGTCTAT
ATGTGTGCAAATCGAATTCCCGCGGCCGCCATGGCGGCCGGGAGCATGCGACGTCGGGCCCAATTCGCCCTATAGTGAGT
CGTATTACAATTCACTGGCCGTCGTTTTACAACGTCGTGACTGGGAAAACCCTGGCGTTACCCAACTTAATCGCCTTGCA
GCACATCCCCCTTTCGCCAGCTGGCGTAATAGCGAAGAGGCCCGCACCGATCGCCCTTCCCAACAGTTGCGCAGCCTGAA
TGGCGAATGGACGCGCCCTGTAGCGGCGCATTAAGCGCGGCGGGTGTGGTGGTTACGCGCAGCGTGACCGCTACACTTGC
CAGCGCCCTAGCGCCCGCTCTTTCGCTTTCTTCCTTCCTTTCTCGCACGTTCGCCGGCTTTCCCCGTCAAGCTCTAAATC
GGGGCTCCTTTAGGGTTCCGAATTAGTGCTTTACGCACCTCGACCCCAAAAACTGAATTAGGGTGGAATGGGTTYAMGCG
TAKGTGGGGGCACACTAYG
>G3.C3
TTKKGCTTKKKGAATKAATTTGGGGCTGTCTTATTTAGGAGTCCTATCAGATGATTATCTGGTTTGCAAGACGGATCACT
TTTTATAGCTGATATTTTATATGTTTTAGCCTCCATTAGAACCTATGTTGTCTTATTTTGGTATTTTGTCATAAATTTGT
ATCATCGGATGTTATAAGTCAATTGCTTCTGAAAATAAGTCAAGGTATGACATACAGAACAAAGTCTGTTATGAAATAAA

TTTCCACTTACTTGATTAAGTTTTATACTTTCAGGTTTGGCTGGTTTGTCTACTGCAAAGTATTTGGCCGATGCAGGTCA
TAAACCTATAGTGTTGGAAGCTAGGGATGTTCTGGGAGGAAAGGTAGCCAAATTATTACTCATTAGTGTTCATGAATTCC
TTGTGGCATAATGGACTGTGTCAAAGTTCAAGGAAAGTCTTTCAAAATTTTCCAGTATATGGATGTGGGAGTTGGTCTAT
ATGTGTGCAAATCGAATTCCCGCGGCCGCCATGGCGGCCGGGAGCATGCGACGTCGGGCCCAATTCGCCCTATAGTGAGT
CGTATTACAATTCACTGGCCGTCGTTTTACAACGTCGTGACTGGGAAAACCCTGGCGTTACCCAACTTTAATCGCCTTGC
AGMACATCCCCCTTTCGCCAGCTGGCGTAAATAGCGAAGAGGCCCGCACCGATCGCCCTTCCCAACAGTTGCGCASCCTG
AAATGGCGAATGGACGCGCCCTGTAGCGGCGCATTAAGCGCGGCGGGTGTGGTGGGTTACGCGCAGCGTGACCSYTACAC
>G3.C4
TGRKGGSATGKGATGAAATTTGGGGCTGTCTTATTTAGGAGTCCTATCAGATGATTATCTGGTTTGCAAGACGGATCACT
TTTTATAGCTGATATTTTATATGTTTTAGCCTCCATTAGAACCTATGTTGTCTTATTTTGGTATTTTGTCATAAATTTGT
ATCATCGGATGTTATAAGTCAATTGCTTCTGAAAATAAGTCAAGGTATGACATACAGAACAAAGTCTGTTATGAAATAAA
TTTCCACTTACTTGATTAAGTTTTATACTTTCAGGTTTGGCTGGTTTGTCTACTGCAAAGTATTTGGCCGATGCAGGTCA
TAAACCTATAGTGTTGGAAGCTAGGGATGTTCTGGGAGGAAAGGTAGCCAAATTATTACTCATTAGTGTTCATGAATTCC
TTGTGGCATAATGGACTGTGTCAAAGTTCAAGGAAAGTCTTTCAAAATTTTCCAGTATATGGATGTGGGAGTTGGTCTAT
ATGTGTGCAAATCGAATTCCCGCGGCCGCCATGGCGGCCGGGAGCATGCGACGTCGGGCCCAATTCGCCCTATAGTGAGT
CGTATTACAATTCACTGGCCGTCGTTTTACAACGTCGTGACTGGGAAAACCCTGGCGTTACCCAACTTAATCGCCTTGCA
GCACATCCCCCTTTCGCCAGCTGGCGTAATAGCGAAGAGGCCCGCACCGATCGCCCTTCCCAACAGTTGCGCAGCCTGAA
TGGCGAATGGACGCGCCCTGTAGCGGCGCATTAAGCGCGGCGGGGTGTGGGTGGTTACGGCGCAGCGTGACCGCTACACT
TGCCAGCGCCCTAGCGCCGCTCCTTTCGCTTTCTTCCCTTCCTWTCTCGCACGTTCGCCGGCTTTCCCCGTCAAGCTCTA
ATCGGGGGGCTCCCTTARGGGTTCCGAATTRTGCCTTTACGGCACCTCGCAACCCTAATACTGAATACGGKCAATGTCAC
GTAGKGGGCCCTACTSSCACCMSATGTATSAT
>G3.C5
TGGKKGCTTGTKGAATGAATTTGGGGCTGTCTTATTTAGGAGTCCTATCAGATGATTATCTGGTTTGCAAGACGGATCAC
TTTTTATAGCTGATATTTTATATGTTTTAGCCTCCATTAGAACCTATGTTGTCTTATTTTGGTATTTTGTCATAAATTTG
TATCATCGGATGTTATAAGTCAATTGCTTCTGAAAATAAGTCAAGGTATGACATACAGAACAAAGTCTGTTATGAAATAA
ATTTCCACTTACTTGATTAAGTTTTATACTTTCAGGTTTGGCTGGTTTGTCTACTGCAAAGTATTTGGCCGATGCAGGTC
ATAAACCTATAGTGTTGGAAGCTAGGGATGTTCTGGGAGGAAAGGTAGCCAAATTATTACTCATTAGTGTTCATGAATTC
CTTGTGGCATAATGGACTGTGTCAAAGTTCAAGGAAAGTCTTTCAAAATTTTCCAGTATATGGATGTGGGAGTTGGTCTA
TATGTGTGCAAATCGAATTCCCGCGGCCGCCATGGCGGCCGGGAGCATGCGACGTCGGGCCCAATTCGCCCTATAGTGAG
TCGTATTACAATTCACTGGCCGTCGTTTTACAACGTCGTGACTGGGAAAACCCTGGCGTTACCCAACTTAATCGCCTTGC
AGCACATCCCCCTTTCGCCAGCTGGCGTAATAGCGAAGAGGCCCGCACCGATCGCCCTTCCCAACAGTTGCGCAGCCTGA
ATGGCGAATGGACGCGCCCTGTAGCGGCGCATTAAGCGCGGCGGGTGTGGTGGTTACGCGCAGCGTGACCGCTACACTTG
CCAGCGCCCTAGCGCCCGCTCCTTTCGCTTTCTTCCCTTCCTTCTCGCACGTTCGCCGGCTTTCCCGTCAAGCTCTAATC
GGGGGCTCCTTTAGGGTTCGATTAGTGCTTACGGCACCTCGAACTCAAAACTGATAGGGGTGAAKGTTYMACGGTWAGGT
GGGCC
>S1.C2
GRKGGCTTKGKGAATGATTTGGGGCTGTCTTATTTAGGAGTCCTATCAGATGATTATCTGGTTTGCAAGACGGATCACTT
TTTATAGCTGATATTTTATATGTTTTAGCCTCCATTAGAACCTATGTTGTCTTATTTTGGTATTTTGTCATAAATTTGTA
TCATCGGATGTTATAAGTCAATTGCTTCTGAAAATAAGTCAAGGTATGACATACAGAACAAAGTCTGTTATGAAATAAAT
TTCCACTTACTTGATTAAGTTTTATACTTTCAGGTTTGGCTGGTTTGTCTACTGCAAAGTATTTGGCCGATGCAGGTCAT
AAACCTATAGTGTTGGAAGCTAGGGATGTTCTGGGAGGAAAGGTAGCCAAATTATTACTCATTAGTGTTCATGAATTCCT
TGTGGCATAATGGACTGTGTCAAAGTTCAAGGAAAGTCTTTCAAAATTTTCCAGTATATGGATGTGGGAGTTGGTCTATA
TGTGTGCAAATCGAATTCCCGCGGCCGCCATGGCGGCCGGGAGCATGCGACGTCGGGCCCAATTCGCCCTATAGTGAGTC
GTATTACAATTCACTGGCCGTCGTTTTACAACGTCGTGACTGGGAAAACCCTGGCGTTACCCAACTTAATCGCCTTGCAG
CACATCCCCCTTTCGCCAGCTGGCGTAATAGCGAAGAGGCCCGCACCGATCGCCCTTCCCAACAGTTGCGCAGCCTGAAT
GGCGAATGGACGCGCCCTGTAGCGGCGCATTAAGCGCGGCGGGTGTGGTGGTTACGCGCAGCGTGACCGCTACACTTGCC
AGCGCCCTAGCGCCCGCTCCTTTCGCTTTCTTCCCTTCCTTTCTCGCACGTTCGCCGGCTTCCCGTCAAGCTCTAAATCG
GGGGGCTCCCTTTAGGGTTCCGATTAGTGCCTTACGGCACTCGACCCAAAAACTGATATGGTGAATGGTCACGGTAGTGG
GCATTCGCACCCCCTGTKCATGT
>S1.C3
TGGRWKGCTKGTGATGATTTGGGGCTGTCTTATTTAGGAGTCCTATCAGATGATTATCTGGTTTGCAAGACGGATCACTT
TTTATAGCTGATATTTTATATGTTTTAGCCTCCATTAGAACCTATGTTGTCTTATTTTGGTATTTTGTCATAAATTTGTA
TCATCGGATGTTATAAGTCAATTGCTTCTGAAAATAAGTCAAGGTATGACATACAGAACAAAGTCTGTTATGAAATAAAT
TTCCACTTACTTGATTAAGTTTTATACTTTCAGGTTTGGCTGGTTTGTCTACTGCAAAGTATTTGGCCGATGCAGGTCAT
AAACCTATAGTGTTGGAAGCTAGGGATGTTCTGGGAGGAAAGGTAGCCAAATTATTACTCATTAGTGTTCATGAATTCCT
TGTGGCATAATGGACTGTGTCAAAGTTCAAGGAAAGTCTTTCAAAATTTTCCAGTATATGGATGTGGGAGTTGGTCTATA
TGTGTGCAAATCGAATTCCCGCGGCCGCCATGGCGGCCGGGAGCATGCGACGTCGGGCCCAATTCGCCCTATAGTGAGTC

GTATTACAATTCACTGGCCGTCGTTTTACAACGTCGTGACTGGGAAAACCCTGGCGTTACCCAACTTAATCGCCTTGCAG
CACATCCCCCTTTCGCCAGCTGGCGTAATAGCGAAGAGGCCCGCACCGATCGCCCTTCCCAACAGTTGCGCAGCCTGAAT
GGCGAATGGACGCGCCCTGTAGCGGCGCATTAAGCGCGGCGGGTGTGGTGGTTACGCGCAGCGTGACCGCTACACTTGCC
AGCGCCCTAGCGCCCGCTCCTTTCGCTTTCTTCCCTTCCTTCTCGCACGTTCGCCGGCTTTCCCGTCAAGCTCTAATCGG
GGGCTCCTTTAGGGTTCGATTTAGTGCTTACGCACTTCGACCCAAAAACTGATAGGGGKGATGGATTCCASGTWRGKGKG
GGGG
>S1.C5
TGRRKGSCTTGTGATGATTTGGGGCTGTCTTATTTAGGAGTCCTATCAGATGATTATCTGGTTTGCAAGACGGATCACTT
TTTATAGCTGATATTTTATATGTTTTAGCCTCCATTAGAACCTATGTTGTCTTATTTTGGTATTTTGTCATAAATTTGTA
TCATCGGATGTTATAAGTCAATTGCTTCTGAAAATAAGTCAAGGTATGACATACAGAACAAAGTCTGTTATGAAATAAAT
TTCCACTTACTTGATTAAGTTTTATACTTTCAGGTTTGGCTGGTTTGTCTACTGCAAAGTATTTGGCCGATGCAGGTCAT
AAACCTATAGTGTTGGAAGCTAGGGATGTTCTGGGAGGAAAGGTAGCCAAATTATTACTCATTAGTGTTCATGAATTCCT
TGTGGCATAATGGACTGTGCCAAAGTTCAAGGAAAGTCTTTCAAAATTTTCCAGTATATGGATGTGGGAGTTGGTCTATA
TGTGTGCAAATCGAATTCCCGCGGCCGCCATGGCGGCCGGGAGCATGCGACGTCGGGCCCAATTCGCCCTATAGTGAGTC
GTATTACAATTCACTGGCCGTCGTTTTACAACGTCGTGACTGGGAAAACCCTGGCGTTACCCAACTTAATCGCCTTGCAG
CACATCCCCCTTTCGCCAGCTGGCGTAATAGCGAAGAGGCCCGCACCGATCGCCCTTCCCAACAGTTGCGCAGCCTGAAT
GGCGAATGGACGCGCCCTGTAGCGGCGCATTAAGCGCGGCGGGTGTGGTGGTTACGCGCAGCGTGACCGCTACACTTGCC
AGCGCCCTAGCGCCCGCTCCTTTCGCTTTCTTCCCTTCCTTTCTCGCACGTTCGCCGGCTTTCCCCGTCAAGCTCTAATC
GGGGGCTCCCTTAGGGTTCGATTTAGTGCTTACGGCACCTCGACCCCAAAAAACTGATTAAGGGGTGATGGATTCCACGT
TRGTGKGGGCCA
>S1.C6
TKRAWGGCTGTGATGAATTTGGGGCTGTCTTATTTAGGAGTCCTATCAGATGATTATCTGGTTTGCAAGACGGATCACTT
TTTATAGCTGATATTTTATATGTTTTAGCCTCCATTAGAACCTATGTTGTCTTATTTTGGTATTTTGTCATAAATTTGTA
TCATCGGATGTTATAAGTCAATTGCTTCTGAAAATAAGTCAAGGTATGACATACAGAACAAAGTCTGTTATGAAATAAAT
TTCCACTTACTTGATTAAGTTTTATACTTTCAGGTTTGGCTGGTTTGTCTACTGCAAAGTATTTGGCCGATGCAGGTCAT
AAACCTATAGTGTTGGAAGCTAGGGATGTTCTGGGAGGAAAGGTAGCCAAATTATTACTCATTAGTGTTCATGAATTCCT
TGTGGCATAATGGACTGTGCCAAAGTTCAAGGAAAGTCTTTCAAAATTTTCCAGTATATGGATGTGGGAGTTGGTCTATA
TGTGTGCAAATCGAATTCCCGCGGCCGCCATGGCGGCCGGGAGCATGCGACGTCGGGCCCAATTCGCCCTATAGTGAGTC
GTATTACAATTCACTGGCCGTCGTTTTACAACGTCGTGACTGGGAAAACCCTGGCGTTACCCAACTTAATCGCCTTGCAG
CACATCCCCCTTTCGCCAGCTGGCGTAATAGCGAAGAGGCCCGCACCGATCGCCCTTCCCAACAGTTGCGCAGCCTGAAT
GGCGAATGGACGCGCCCTGTAGCGGCGCATTAAGCGCGGCGGGTGTGGTGGTTACGCGCAGCGTGACCGCTACACTTGCC
AGCGCCCTAGCGCCCGCTCCTTTCGCTTTCTTCCCTTCCTTTCTCGCACGTTCGCCGGCTTTCCCCGTCAGCTCTAAATC
GGGGGCTCCCTTTAGGTTCGAATTAGTGCTTAACGCACCTCGACCTAATCTGATTAGGTGATGTCACGTAGGKGGGGACC
AAYAGTG
>S2.C2
AGGGKTGGGRRGTTKGGTYTWTGTGTGCAATCGAATTCCCGCGGCCGCCATGGCGGCCGGGAGCATGCGACGTCGGGCCC
AATTCGCCCTATAGTGAGTCGTATTACAATTCACTGGCCGTCGTTTTACAACGTCGTGACTGGGAAAACCCTGGCGTTAC
CCAACTTAATCGCCTTGCAGCACATCCCCCTTTCGCCAGCTGGCGTAATAGCGAAGAGGCCCGCACCGATCGCCCTTCCC
AACAGTTGCGCAGCCTGAATGGCGAATGGACGCGCCCTGTAGCGGCGCATTAAGCGCGGCGGGTGTGGTGGTTACGCGCA
GCGTGACCGCTACACTTGCCAGCGCCCTAGCGCCCGCTCCTTTCGCTTTCTTCCCTTCCTTTCTCGCCACGTTCGCCGGC
TTTCCCCGTCAAGCTCTAAATCGGGGGCTCCCTTTAGGGTTCCGATTTAGTGCTTTACGGCACCTCGACCCCAAAAAACT
TGATTAGGGTGATGGTTCACGTAGTGGGCCATCGCCCTGATAGACGGTTTTTCGCCCTTTGACGTTGGAGTCCACGTTCT
TTAATAGTGGACTCTTGTTCCAAACTGGAACAACACTCAACCCTATCTCGGTCTATTCTTTTGATTTATAAGGGATTTTG
CCGATTTCGGCCTATTGGTTAAAAAATGAGCTGATTTAACAAAAATTTAACGCGAATTTTAACAAAATATTAACGCTTAC
AATTTCCTGATGCGGTATTTTCTCCTTACGCATCTGTGCGGTATTTCACACCGCATCAGGTGGCACTTTTCGGGGAAATG
TGCGCGGAACCCCTATTTGTTTATTTTTCTAAATACATTCAAATATGTATCCGCTCATGAGACAATAACCCTGATAAATG
CTTCATAATATGAAAAAGAGAGTATGAGTATCACATTTCCGTGTCGCCCTTATTCCCTTTTTGGCGGCAGTTTTGGCCTT
TCCKGGTTTTTK
>S2.C4
CCMCCCCCTTTTCCGCCACGCTAGGCKAATAGCGAAGAGGCCCGCACCGATCGCCCTTCCCAACAGTTGCGCAGCCTGAA
TGGCGAATGGACGCGCCCTGTAGCGGCGCATTAAGCGCGGCGGGTGTGGTGGTTACGCGCAGCGTGACCGCTACACTTGC
CAGCGCCCTAGCGCCCGCTCCTTTCGCTTTCTTCCCTTCCTTTCTCGCCACGTTCGCCGGCTTTCCCCGTCAAGCTCTAA
ATCGGGGGCTCCCTTTAGGGTTCCGATTTAGTGCTTTACGGCACCTCGACCCCAAAAAACTTGATTAGGGTGATGGTTCA
CGTAGTGGGCCATCGCCCTGATAGACGGTTTTTCGCCCTTTGACGTTGGAGTCCACGTTCTTTAATAGTGGACTCTTGTT
CCAAACTGGAACAACACTCAACCCTATCTCGGTCTATTCTTTTGATTTATAAGGGATTTTGCCRATTYCGGCCTATTGGT
TAAAAAATGAGCTGATTTACMAAMATTTAACGCGAATTTTTYCAAATATTAACGCTTACAATTTYCCTGATGCGGTATTT
TCTCCTTACSCATCTGTGCGGTATTTCACCMCGCCTCAGGTGGSACYTTTCGGGGAAATGTGCCCGGAACCACTATTT

>S2.C6
TGAAWKGGCCTGTGAATGAATTTGGGGCTGTCTTATTTAGGAGTCCTATCAGATGATTATCTGGTTTGCAAGACGGATCA
CTTTTTATAGCTGATATTTTATATGTTTTAGCCTCCATTAGAACCTATGTTGTCTTATTTTGGTATTTTGTCATAAATTT
GTATCATCGGATGTTATAAGTCAATTGCTTCTGAAAATAAGTCAAGGTATGACATACAGAACAAAGTCTGTTATGAAATA
AATTTCCACTTACTTGATTAAGTTTTATACTTTCAGGGATGTTCTGGGAGGAAAGGTAGCCAAATTATTACTCATTAGTG
TTCATGAATTCCTTGTGGCATAATGGACTGTGTCAAAGTTCAAGGAAAGTCTTTCAAAATTTTCCAGTATATGGATGTGG
GAGTTGGTCTATATGTGTGCAAATCGAATTCCCGCGGCCGCCATGGCGGCCGGGAGCATGCGACGTCGGGCCCAATTCGC
CCTATAGTGAGTCGTATTACAATTCACTGGCCGTCGTTTTACAACGTCGTGACTGGGAAAACCCTGGCGTTACCCAACTT
AATCGCCTTGCAGCACATCCCCCTTTCGCCAGCTGGCGTAATAGCGAAGAGGCCCGCACCGATCGCCCTTCCCAACAGTT
GCGCAGCCTGAATGGCGAATGGACGCGCCCTGTAGCGGCGCATTAAGCGCGGCGGGTGTGGTGGTTACGCGCAGCGTGAC
CGCTACACTTGCCAGCGCCCTAGCGCCCGCTCCTTTCGCTTTCTTCCCTTCCTTTCTCGCCACGTTCGCCGGCTTTCCCC
GTCAAGCTCTAAATCGGGGGCTCCCTTTAGGGTTCCGATTTAGTGCTTTACGGCACCTCGACCCCAAAAAACTTGATTAG
GGTGATGGGTTCACGTAGTGGGCCATCGCCTGATAGACGGTTTTCGCCGTTTGACGTGGRCTCACGCTCTATAGTGAACT
CTTGATCCAAACCGTKGGGACTAAC
>S2.C9
TGRKKGSTKGTGAAWGAATTTGGGGGCTGTCTTATTTAGGAGTCCTATCAGATGATTATCTGGTTTGCAAGACGGATCAC
TTTTTATAGCTGATATTTTATATGTTTTAGCCTCCATTAGAACCTATGTTGTCTTATTTTGGTATTTTGTCATAAATTTG
TATCATCGGATGTTATAAGTCAATTGCTTCTGAAAATAAGTCAAGGTATGACATACAGAACAAAGTCTGTTATGAAATAA
ATTTCCACTTACTTGATTAAGTTTTATACTTTCAGGTTTGGCTGGTTTGTCTACTGCAAAGTATTTGGCTGCAGGTCATA
AACCTATAGTGTTGGAAGCTAGGGATGTTCTGGGAGGAAAGGTAGCCAAATTATTACTCATTAGTGTTCATGAATTCCTT
GTGGCATAATGGACTGTGTCAAAGTTCAAGGAAAGTCTTTCAAAATTTTCCAGTATATGGATGTGGGAGTTGGTCTATAT
GTGTGCAAATCGAATTCCCGCGGCCGCCATGGCGGCCGGGAGCATGCGACGTCGGGCCCAATTCGCCCTATAGTGAGTCG
TATTACAATTCACTGGCCGTCGTTTTACAACGTCGTGACTGGGAAAACCCTGGCGTTACCCAACTTAATCGCCTTGCAGC
ACATCCCCCTTTCGCCAGCTGGCGTAATAGCGAAGAGGCCCGCACCGATCGCCCTTCCCAACAGTTGCGCAGCCTGAATG
GCGAATGGACGCGCCCTGTAGCGGCGCATTAAGCGCGGCGGGTGTGGTGGTTACGCGCAGCGTGACCGCTACACTTGCCA
GCGCCCTAGCGCCCGCTCCTTTCGCTTTCTTCCCTTCCTTTCTCGCACGTTCGCCGGCTTTCCCGTCAGCTCTAATCGGG
GGCTCCTTAGGGTTCCGATTTAGTGCTTACGCACTCGACCCAAAAAACTGAATTAGGGKTGATGGATTCAACGTWRGKGT
GGGCCAAC
>S3.C1
GKRRKKGGSCKKGGKRAAKGAATTTGGGGGCTGTCTTATTTAGGAGTCCTATCAGATGATTATCTGGTTTGCAAGACSGA
TCACTTTTTATAGCTGATATTTTATATGTTTTAAATCGAATTCCCGCGGCCGCCATGGCGGCCGGGAGCATGCGACGTCG
GGCCCAATTCGCCCTATAGTGAGTCGTATTACAATTCACTGGCCGTCGTTTTACAACGTCGTGACTGGGAAAACCCTGGC
GTTACCCAACTTAATCGCCTTGCAGCACATCCCCCTTTCGCCAGCTGGCGTAATAGCGAAGAGGCCCGCACCGATCGCCC
TTCCCAACAGTTGCGCAGCCTGAATGGCGAATGGACGCGCCCTGTAGCGGCGCATTAAGCGCGGCGGGTGTGGTGGTTAC
GCGCAGCGTGACCGCTACACTTGCCAGCGCCCTAGCGCCCGCTCCTTTCGCTTTCTTCCCTTCCTTTCTCGCCACGTTCG
CCGGCTTTCCCCGTCAAGCTCTAAATCGGGGGCTCCCTTTAGGGTTCCGATTTAGTGCTTTACGGCACCTCGACCCCAAA
AAACTTGATTAGGGTGATGGTTCACGTAGTGGGCCATCGCCCTGATAGACGGTTTTTCGCCCTTTGACGTTGGAGTCCAC
GTTCTTTAATAGTGGACTCTTGTTCCAAACTGGAACAACACTCAACCCTATCTCGGTCTATTCTTTTGATTTATAAGGGA
TTTTGCCGATTTCGGCCTATTGGTTAAAAAATGAGCTGATTTAACAAAAATTTAACGCGAATTTTAACAAAATATTAACGC
TTACATTTCCTGATGCGGTATTTTCTCCTTACGCATCTGTGCGGTATTTCACACCGCATCAGGTGGCACTTTTCGGGGAA
TGTGCGCGSAMCCCCTATTTGTTTATTTTTCTAATACATTCCAATWKGTATCGCTCATGAGAAMAATAACCCTGGAWAAA
KGCTTTYMA
>S3.C3
AGRRRWGSCTTGTKGAATGAATTTGGGGGCTGTCTTATTTAGGAGTCCTATCAGATGATTATCTGGTTTGCAAGACGGAT
CACTTTTTATAGCTGATATTTTATATGTTTTAGCCTCCATTAGAACCTATGTTGTCTTATTTTGGTATTTTGTCATAAAT
TTGTATCATCGGATGTTATAAGTCAATTGCTTCTGAAAATAAGTCAAGGTATGACATACAGAACAAAGTCTGTTATGAAA
TAAATTTCCACTTACTTGATTAAGTTTTATACTTTCAGGTTTGGCTGGTTTGTCTACTGCAAAGTATTTGGCTGCAGGTC
ATAAACCTATAGTGTTGGAAGCTAGGGATGTTCTGGGAGGAAAGGTAGCCAAATTATTACTCATTAGTGTTCATGAATTC
CTTGTGGCATAATGGACTGTGTCAAAGTTCAAGGAAAGTCTTTCAAAATTTTCCAGTATATGGATGTGGGAGTTGGTCTA
TATGTGTGCAAATCACTAGTGAATTCGCGGCCGCCTGCAGGTCGACCATATGGGAGAGCTCCCAACGCGTTGGATGCATA
GCTTGAGTATTCTATAGTGTCACCTAAATAGCTTGGCGTAATCATGGTCATAGCTGTTTCCTGTGTGAAATTGTTATCCG
CTCACAATTCCACACAACATACGAGCCGGAAGCATAAAGTGTAAAGCCTGGGGTGCCTAATGAGTGAGCTAACTCACATT
AATTGCGTTGCGCTCACTGCCCGCTTTCCAGTCGGGAAACCTGTCGTGCCAGCTGCATTWATGAATCGGCCAACGCGCGG
GGAGAGGCGGTTTGCGTATTGGGCGCTCTTCCGCTTCCTCGCTCACTGACTCGCTGCGCTCGGTCGTTCGGCTGGCGGSG
AGCGATATCAGCTCACTCAAAGGGCGGTAATACGGTTATCCACAGAATCAGGGGATAACGCCATGAAGAAMCATGKTGTA
GCAAARGGGCCGAGCCTAAG
>S3.C4

TRWKGGCTGTGATGAATTTGGGGCTGTCTTATTTAGGAGTCCTATCAGATGATTATCTGGTTTGCAAGACGGATCACTTT
TTATAGCTGATATTTTATATGTTTTAGCCTCCATTAGAACCTATGTTGTCTTATTTTGGTATTTTGTCATAAATTTGTAT
CATCGGATGTTATAAGTCAATTGCTTCTGAAAATAAGTCAAGGTATGACATACAGAACAAAGTCTGTTATGAAATAAATT
TCCACTTACTTGATTAAGTTTTATACTTTCAGGTTTGGCTGGTTTGTCTACTGCAAAGTATTTGGCCGATGCAGGTCATA
AACCTATAGTGTTGGAAGCTAGGGATGTTCTGGGAGGAAAGGTAGCCAAATTATTACTCATTAGTGTTCATGAATTCCTT
GTGGCATAATGGACTGTGTCAAAGTTCAAGGAAAGTCTTTCAAAATTTTCCAGTATATGGATGTGGGAGTTGGTCTATAT
GTGTGCAAATCGAATTCCCGCGGCCGCCATGGCGGCCGGGAGCATGCGACGTCGGGCCCAATTCGCCCTATAGTGAGTCG
TATTACAATTCACTGGCCGTCGTTTTACAACGTCGTGACTGGGAAAACCCTGGCGTTACCCAACTTAATCGCCTTGCAGC
ACATCCCCCTTTCGCCAGCTGGCGTAATAGCGAAGAGGCCCGCACCGATCGCCCTTCCCAACAGTTGCGCAGCCTGAATG
GCGAATGGACGCGCCCTGTAGCGGCGCATTAAGCGCGGCGGGTGTGGTGGTTACGCGCAGCGTGACCGCTACACTTGCCA
GCGCCCTAGCGCCCGCTCCTTTCGCTTTCTTCCCTTCCTTCTCGCACGTTCGCGGCTTCCCGTCAAGCTCTAATCGGGGG
CTCCTTAGGGTTCGATTTAGTGCTTACGGCCACTCGAACCCCAAAAAACTGATTAGGKATGGTCGGTACTGGCCAATSGC
CGTGTATAGTRC
>S3.C5
GAWGCTGTGATGATTTGGGGCTGTCTTATTTAGGAGTCCTATCAGATGATTATCTGGTTTGCAAGACGGATCACTTTTTA
TAGCTGATATTTTATATGTTTTAGCCTCCATTAGAACCTATGTTGTCTTATTTTGGTATTTTGTCATAAATTTGTATCAT
CGGATGTTATAAGTCAATTGCTTCTGAAAATAAGTCAAGGTATGACATACAGAACAAAGTCTGTTATGAAATAAATTTCC
ACTTACTTGATTAAGTTTTATACTTTCAGGTTTGGCTGGTTTGTCTACTGCAAAGTATTTGGCTGCAGGTCATAAACCTA
TAGTGTTGGAAGCTAGGGATGTTCTGGGAGGAAAGGTAGCCAAATTATTACTCATTAGTGTTCATGAATTCCTTGTGGCA
TAATGGACTGTGTCAAAGTTCAAGGAAAGTCTTTCAAAATTTTCCAGTATATGGATGTGGGAGTTGGTCTATATGTGTGC
AAATCGAATTCCCGCGGCCGCCATGGCGGCCGGGAGCATGCGACGTCGGGCCCAATTCGCCCTATAGTGAGTCGTATTAC
AATTCACTGGCCGTCGTTTTACAACGTCGTGACTGGGAAAACCCTGGCGTTACCCAACTTAATCGCCTTGCAGCACATCC
CCCTTTCGCCAGCTGGCGTAATAGCGAAGAGGCCCGCACCGATCGCCCTTCCCAACAGTTGCGCAGCCTGAATGGCGAAT
GGACGCGCCCTGTAGCGGCGCATTAAGCGCGGCGGGTGTGGTGGTTACGCGCAGCGTGACCGCTACACTTGCCAGCGCCC
TAGCGCCCGCTCCTTTCGCTTTCTTCCCTTCCTTTCTCGCCACGTTCGCCGGCTTTCCCCGTCAAGCTCTAATCGGGGGG
CTCCTTTTAGGGTTCCGATTAGTGCTTACGGCACTTCGAACCCCAAAAAACTTGATTAGGTGATGGATTCMMSGTWARCC
G
>S4.C2
TGWKGCATGTGATGATTTGGGGCTGTCTTATTTAGGAGTCCTATCAGATGATTATCTGGTTTGCAAGACGGATCACTTTT
TATAGCTGATATTTTATATGTTTTAGCCTCCATTAGAACCTATGTTGTCTTATTTTGGTATTTTGTCATAAATTTGTATC
ATCGGATGTTATAAGTCAATTGCTTCTGAAAATAAGTCAAGGTATGACATACAGAACAAAGTCTGTTATGAAATAAATTT
CCACTTACTTGATTAAGTTTTATACTTTCAGGTTTGGCTGGTTTGTCTACTGCAAAGTATTTGGCCGATTGCAGGTCATA
AACCTATAGTGTTGGAAGCTAGGGATGTTCTGGGAGGAAAGGTAGCCAAATTATTACTCATTAGTGTTCATGAATTCCTT
GTGGCATAATGGACTGTGTCAAAGTTCAAGGAAAGTCTTTCAAAATTTTCCAGTATATGGATGTGGGAGTTGGTCTATAT
GTGTGCAAATCGAATTCCCGCGGCCGCCATGGCGGCCGGGAGCATGCGACGTCGGGCCCAATTCGCCCTATAGTGAGTCG
TATTACAATTCACTGGCCGTCGTTTTACAACGTCGTGACTGGGAAAACCCTGGCGTTACCCAACTTAATCGCCTTGCAGC
ACATCCCCCTTTCGCCAGCTGGCGTAATAGCGAAGAGGCCCGCACCGATCGCCCTTCCCAACAGTTGCGCAGCCTGAATG
GCGAATGGACGCGCCCTGTAGCGGCGCATTAAGCGCGGCGGGTGTGGTGGTTACGCGCAGCGTGACCGCTACACTTGCCA
GCGCCCTAGCGCCCGCTCCTTTCGCTTTCTTCCTTCTTTCTCGCACGTTCGCCGGCTTTCCCCGTCAGCTCTAATCGGGG
CTCCCTTTAGGTTCGATTAGTGCTTAMCGCACCTCGACCCCAAAAAACTTGGATTAGGGGKAATGGGGTTYA
>S4.C3
AWGGSYYTTKKKAATGAATTTGGGGCTGTCTTATTTAGGAGTCCTATCAGATGATTATCTGGTTTGCAAGACGGATCTCT
TTTTATAGCTGATATTTTATATGTTTTAGCCTCCATTATTAACCTATGTTGTCTTATTTTGGTATTTTGTCATAAATTTG
TATCATCGGATGTTATAAGTCAATTGCTTCTGAAAATAARTCAAGGTATGACATACAGAACAAAGTCTGTTATGAAATAA
ATTTCCACTTACTTGATTAAGTTTTATACTTTCAGGTTTGGCTGGTTTGTCTACTGYAAAGTATTTGGCCGACTGCRGGT
CATAAACCTATAGTGTTGGAAGCTAGGGATGTYCTGGGAGGAAAGGWARCCAAATTATTACTCATTAGTGWTCATGAATT
CCT
>S4.C4
GAWKGCATGTGATGATTTGGGGCTGTCTTATTTAGGAGTCCTATCAGATGATTATCTGGTTTGCAAGACGGATCACTTTT
TATAGCTGATATTTTATATGTTTTAGCCTCCATTAGAACCTATGTTGTCTTATTTTGGTATTTTGTCATAAATTTGTATC
ATCGGATGTTATAAGTCAATTGCTTCTGAAAATAAGTCAAGGTATGACATACAGAACAAAGTCTGTTATGAAATAAATTT
CCACTTACTTGATTAAGTTTTATACTTTCAGGTTTGGCTGGTTTGTCTACTGCAAAGTATTTGGCCGACTGCAGGTCAAA
TCGAATTCCCGCGGCCGCCATGGCGGCCGGGAGCATGCGACGTCGGGCCCAATTCGCCCTATAGTGAGTCGTATTACAAT
TCACTGGCCGTCGTTTTACAACGTCGTGACTGGGAAAACCCTGGCGTTACCCAACTTAATCGCCTTGCAGCACATCCCCC
TTTCGCCAGCTGGCGTAATAGCGAAGAGGCCCGCACCGATCGCCCTTCCCAACAGTTGCGCAGCCTGAATGGCGAATGGA
CGCGCCCTGTAGCGGCGCATTAAGCGCGGCGGGTGTGGTGGTTACGCGCAGCGTGACCGCTACACTTGCCAGCGCCCTAG
CGCCCGCTCCTTTCGCTTTCTTCCCTTCCTTTCTCGCCACGTTCGCCGGCTTTCCCCGTCAAGCTCTAAATCGGGGGCTC

CCTTTAGGGTTCCGATTTAGTGCTTTACGGCACCTCGACCCCAAAAAACTTGATTAGGGTGATGGTTCACGTAGTGGGCC
ATCGCCCTGATAGACGGTTTTTCGCCCTTTGACGTTGGAGTCCACGTTCTTTAATAGTGGACTCTTGTTCCAACTGGGAA
CAACACTCAACCCTATCTCGTCTATTCTTTGATTATAGGCATTTTGCCGATTTCGGCCTATTTGAATTTAAAAAAAAT
>S4.C5
CRGGSCTKTTGAWTGAATTTGGGGCTGTCTTATTTAGGAGTCCTATCAGATGATTATCTGGTTTGCAAGACGGATCACTT
TTTATAGCTGATATTTTATATGTTTTAGCCTCCATTAGAACCTATGTTGTCTTATTTTGGTATTTTGTCATAAATTTGTA
TCATCGGATGTTATAAGTCAATTGCTTCTGAAAATAAGTCAAGGTATGACATACAGAACAAAGTCTGTTATGAAATAAAT
TTCCACTTACTTGATTAAGTTTTATACTTTCAGGTTTGGCTGGTTTGTCTACTGCAAAGTATTTGGCCGATTGCAGGTCA
TAAACCTATAGTGTTGGAAGCTAGGGATGTTCTGGGAGGAAAGGTAGCCAAATTATTACTCATTAGTGTTCATGAATTCC
TTGTGGCATAATGGACTGTGTCAAAGTTCAAGGAAAGTCTTTCAAAATTTTCCAGTATATGGATGTGGGAGTTGGTCTAT
ATGTGTGCAAATCGAATTCCCGCGGCCGCCATGGCGGCCGGGAGCATGCGACGTCGGGCCCAATTCGCCCTATAGTGAGT
CGTATTACAATTCACTGGCCGTCGTTTTACAACGTCGTGACTGGGAAAACCCTGGCGTTACCCAACTTAATCGCCTTGCA
GCACATCCCCCTTTCGCCAGCTGGCGTAATAGCGAAGAGGCCCGCACCGATCGCCCTTCCCAACAGTTGCGCAGCCTGAA
TGGCGAATGGACGCGCCCTGTAGCGGCGCATTAAGCGCGGCGGGTGTGGTGGTTACGCGCAGCGTGACCGCTACACTTGC
CAGCGCCCTAGCGCCCGCTCCTTTCGCTTTCTTCCCTTCCTTTCTCGCCACGTTCGCCGGCTTTCCCCGTCAAGCTCTAA
TCGGGGGGCTCCCTTTAGGGTTCCGAATTAGTGCTTTACGCACTTCGACCCCAAACCTTGATTAGGGGTGAAAKGATTTC
ACMGSTKA

Supplementary dataset 2: Partial genomic DNA sequencing of *CcPDS* corresponding to sgRNA target 2 (exon 5). Abbreviations refer to the following samples: P: albino somatic seedlings, WT: control, untransformed; G: green seedlings (transformed); S: variegated seedlings (transformed); and C: different clones sequenced.

>P2.C1
CCYKKKGRSRGTWAWACCMTCTTGGTGCCTCAACATAAGATTGTCCACCCAGAATTGCTGGCAAGAGTCCAATTGCAAAT
TTGACTTTCTCTGGCCAAGTAAGCATGTCATTATTCTTCAAGATGGCCCATATTCCTGCAAGACCAATTTGAACACAATA
TCCACATAAAAACAAGTTACTTCATTTGAAATTTGGCTGCACAAATTAGCTCACCATTTAATGGTGCTGGTAGCACCTCA
GGAAAATCAAATCGACTGAACTCTCCAGGCTTATTTGGCATTGCAAATATCATTGAATGCTCCTTCCACTGACAACCGAT
CATTAATTCCTAGTTCTCCAAACAGGTTCTGCATATTTGGGTAAGCCCCAACTTAAGGTGGATCAAGAATCAATGTCACA
AGTTATTTGCTGCATCATAATGTATCTTAAAAAGAAACAAGAAGAATCATCTACATTCATGCTCATAAGAAATTCAAAAA
AGCACGGAACAAGATAAGGCAAATAATCGAATTCCCGCGGCCGCCATGGCGGCCGGGAGCATGCGACGTCGGGCCCAATT
CGCCCTATAGTGAGTCGTATTACAATTCACTGGCCGTCGTTTTACAACGTCGTGACTGGGAAAACCCTGGCGTTACCCAA
CTTAATCGCCTTGCAGCACATCCCCCTTTCGCCAGCTGGCGTAATAGCGAAGAGGCCCGCACCGATCGCCCTTCCCAACA
GTTGCGCAGCCTGAATGGCGAATGGACGCGCCCTGTAGCGGCGCATTAAGCGCGGCGGGTGTGGTGGTTACGCGCAGCGT
GACCGCTACACTTGCCAGCGCCCTAGCGCCGCTCTTTCGCTTTCTTCCCTTCTTTCTCGCACGTTCGCCGGCTTTCCCGT
CAGCTCTAATCGGGGGCTCCTTAGGGTTCGATTAGTGCTTACGGCACTCGACCCAAAAACTGATAGGGTATGTCMGGTAC
KGGGACATGCCCGTGTATATAGAGCCG
>P6.C3
GWYCCMMMCCCCMRGAAATTTGCTGGCAAGAGTCCAATTGCMAWCTTTWGAMTTTCTCTGGCCAAGTAAGCATGTCATTA
TTCTTCAWGATGGCCCATATTCCTGCAAGACCAATTTGAACACAATATCCACATAAAAACAAGTTACTTCATTTGAAATT
TGGCTGCACAAATTAGCTCACCATTTAATGGTGCTGGTAGCACCTCAGGAAAATCAAATCGACTGAACTCTCCAGGCTTA
TTTGGCATTGCAAATATCATTGAATGCTCCTTCCACTGCCAACCGATCATTAATTCCTAGTTCTCCAAACAGGTTCTGCA
TATTTGGGTAAGCCCCAACTTAAGGTGGATCAAGAATCAATGTCACAAGTTATTTGCTGCATCATAATGTATCTTAAAAA
GAAACAAGAAGAATCATCTACMTTCATGCTCATAARAAATTCMAAAAAGCWCGGAACWAGATAAGGCAAATAATCTAATT
CYCGCGGKCGCCATGGCGGCCGGAATCATGCRACGTCG
>P6.C4
ACYTKKMMGTTWWACCATCTTGTGCCTCAACATAAGATTGTCCACCCAGAATTGCTGGCAAGAGTCCAATTGCAAATTTG
ACTTTCTCTGGCCAAGTAAGCATGTCATTATTCTTCAAGATGGCCCATATTCCTGCAAGACCAATTTGAACACAATATCC
ACATAAAAACAAGTTACTTCATTTGAAATTTGGCTGCACAAATTAGCTCACCATTTAATGGTGCTGGTAGCACCTCAGGA
AAATCAAATCGACTGAACTCTCCAGGCTTATTTGGCATTGCAAATATCATTGAATGCTCCTTCCACTGCCAACCGATCAT
TAATTCCTAGTTCTCCAAACAGGTTCTGCATATTTGGGTAAGCCCCAACTTAAGGTGGATCAAGAATCAATGTCACAAGT
TATTTGCTGCATCATAATGTATCTTAAAAAGAAACAAGAAGAATCATCTACATTCATGCTCATAAGAAATTCAAAAAAGC
ACGGAACAAGATAAGGCAAAAATCGAATTCCCGCGGCCGCCATGGCGGCCGGGAGCATGCGACGTCGGGCCCAATTCGCC
CTATAGTGAGTCGTATWACAATTCACTGGCCGTCGTTTTACAACGTCGTGACTGGGAAAACCCTGGCGTTACCCAACTTA
ATCGCCTTGCAGCACATCCCCCTTTCSCCCAGCTGGCGTAAATASC
>P7.C1
ACTKTMRGTWWACCATCTTGTGCCTCAACATAAGATTGTCCACCCAGAATTGCTGGCAAGAGTCCAATTGCAAATTTGAC
TTTCTCTGGCCAAGTAAGCATGTCATTATTCTTCAAGATGGCCCATATTCCTGCAAGACCAATTTGAACACAATATCCAC
ATAAAAACAAGTTACTTCATTTGAAATTTGGCTGCACAAATTAGCTCACCATTTAATGGTGCTGGTAGCACCTCAGGAAA
ATCAAATCGACTGAACTCTCCAGGCTTATTTGGCATTGCAAATATCATTGAATGCTCCTTCCACAACCGATCATTAATTC
CTAGTTCTCCAAACAGGTTCTGCATATTTGGGTAAGCCCCAACTTAAGGTGGATCAAGAATCAATGTCACAAGTTATTTG
CTGCATCATAATGTATCTTAAAAAGAAACAAGAAGAATCATCTACATTCATGCTCATAAGAAATTCAAAAAAGCACGGAA
CAAGATAAGGCAAATAAATCGAATTCCCGCGGCCGCCATGGCGGCCGGGAGCATGCGACGTCGGGCCCAATTCGCCCTAT
AGTGAGTCGTATTACAATTCACTGGCCGTCGTTTTACAACGTCGTGACTGGGAAAACCCTGGCGTTACCCAACTTAATCG
CCTTGCAGCACATCCCCCTTTCGCCAGCTGGCGTAATAGCGAAGAGGCCCGCACCGATCGCCCTTCCCAACAGTTGCGCA
GCCTGAATGGCGAATGGACGCGCCCTGTAGCGGCGCATTAAGCGCGGCGGGGTGTGGTGGTTACGCGCAGCGTGACCGCT
ACACTTGCCAGCGCCCTAGCGCCCGCTCCTTTCGCTTTCTTCCCTTCCTTTCTCGCCACGTTSGCCGGCTTTCCCCGTCA
AGCTCTAAATCGGGGGGGCTCCCTTTAGGGTTCGAATTAGTGCCTTACCGCACCTTCGACCCAAAACCTTGGATTARCGA
TGTAGTGAGTTYAS
>P7.C2
GWMRGKTTWWWCMATCTTGTGGCCTCAACATAAGATTGTCCACCCAGAATTGCTGGCAAGAGTCCAATTGCAAATTTGAC
TTTCTCTGGCCAAGTAAGCATGTCATTATTCTTCAAGATGGCCCATATTCCTGCAAGACCAATTTGAACACAATATCCAC
ATAAAAACAAGTTACTTCATTTGAAATTTGGCTGCACAAATTAGCTCACCATTTAATGGTGCTGGTAGCACCTCAGGAAA
ATCAAATCGACTGAACTCTCCAGGCTTATTTGGCATTGCAAATATCATTGAATGCTCCTTCCACAACCGATCATTAATTC
CTAGTTCTCCAAACAGGTTCTGCATATTTGGGTAAGCCCCAACTTAAGGTGGATCAAGAATCAATGTCACAAGTTATTTG

CTGCATCATAATGTATCTTAAAAAGAAACAAGAAGAATCATCTACATTCATGCTCATAAGAAATTCAAAAAAGCACGGAA
CAAGATAAGGCAAATAAATCGAATTCCCGCGGCCGCCATGGCGGCCGGGAGCATGCGACGTCGGGCCCAATTCGCCCTAT
AGTGAGTCGTATTACAATTCACTGGCCGTCGTTTTACTCGTCGTGACTGGGAAAACCCTGGCGTTACCCAACTTAATCGC
CTTGCAGCACATCCCCCTTTCGCCAGCTGGCGTAATAGCKAAGAGGCCCGCACCGATCGCCCWTCCCAACAGTTYGCCCA
CCTGAAGGMMAATGGGACA
>P7.C3
GCTKKKMSGTWWCMTCTTGTGCCTCAACATAAGATTGTCCACCCAGAATTGCTGGCAAGAGTCCAATTGCAAATTTGACT
TTCTCTGGCCAAGTAAGCATGTCATTATTCTTCAAGATGGCCCATATTCCTGCAAGACCAATTTGAACACAATATCCACA
TAAAAACAAGTTACTTCATTTGAAATTTGGCTGCACAAATTAGCTCACCATTTAATGGTGCTGGTAGCACCTCAGGAAAA
TCAAATCGACTGAACTCTCCAGGCTTATTTGGCATTGCAAATATCATTGAATGCTCCTTCCACAACCGATCATTAATTCC
TAGTTCTCCAAACAGGTTCTGCATATTTGGGTAAGCCCCAACTTAAGGTGGATCAAGAATCAATGTCACAAGTTATTTGC
TGCATCATAATGTATCTTAAAAAGAAACAAGAAGAATCATCTACATTCATGCTCATAAGAAATTCAAAAAAGCACGGAAC
AAGATAAGGCAAATAAATCGAATTCCCGCGGCCGCCATGGCGGCCGGGAGCATGCGACGTCGGGCCCAATTCGCCCTATA
GTGAGTCGTATTACAATTCACTGGCCGTCGTTTTACAACGTCGTGACTGGGAAAACCCTGGCGTTACCCAACTTAATCGC
CTTGCAGCACATCCCCCTTTCGCCAGCTGGCGTAATAGCGAAGAGGCCCGCACCGATCGCCCTTCCCAACAGTTGCGCAG
CCTGAATGGCGAATGGACGCGCCCTGTAGCGGCGCATTAAGCGCGGCGGGTGTGGTGGTTACGCGCAGCGTGACCGCTAC
ACTTGCCAGCGCCCTAGCGCCCGCTCCTTTCGCTTTCTTCCCTTCCTTTCTCGCCACGTTCGCCGGCTTTCCCCGTCAAGC
TCTAAATCGGGGGCTCCCTTTAAGGTTCGATTTAGTGCTTACGGCACCTCGAACCCAAAAAAACTTGGATWRRGGGGTGA
ATGGGGAT
>P7.C4
ACTKKKMGKTWWCMTCTTGTGCCTCAACATAAGATTGTCCACCCAGAATTGCTGGCAAGAGTCCAATTGCAAATTTGACT
TTCTCTGGCCAAGTAAGCATGTCATTATTCTTCAAGATGGCCCATATTCCTGCAAGACCAATTTGAACACAATATCCACA
TAAAAACAAGTTACTTCATTTGAAATTTGGCTGCACAAATTAGCTCACCATTTAATGGTGCTGGTAGCACCTCAGGAAAA
TCAAATCGACTGAACTCTCCAGGCTTATTTGGCATTGCAAATATCATTGAATGCTCCTTCCACAACCGATCATTAATTCC
TAGTTCTCCAAACAGGTTCTGCATATTTGGGTAAGCCCCAACTTAAGGTGGATCAAGAATCAATGTCACAAGTTATTTGC
TGCATCATAATGTATCTTAAAAAGAAACAAGAAGAATCATCTACATTCATGCTCATAAGAAATTCAAAAAAGCACGGAAC
AAGATAAGGCAAATAAATCGAATTCCCGCGGCCGCCATGGCGGCCGGGAGCATGCGACGTCGGGCCCAATTCGCCCTATA
GTGAGTCGTATTACAATTCACTGGCCGTCGTTTTACAACGTCGTGACTGGGAAAACCCTGGCGTTACCCAACTTAATCGC
CTTGCAGCACATCCCCCTTTCGCCAGCTGGCGTAATAGCGAAGAGGCCCGCACCGATCGCCCTTCCCAACAGTTGCGCAG
CCTGAATGGCGAATGGACGCGCCCTGTAGCGGCGCATTAAGCGCGGCGGGTGTGGTGGTTACGCGCAGCGTGACCGCTA
CACTTGCCAGCGCCCTAGCGCCCGCTCCTTTTCGCTTTCTTCCCTTCCTTTCTCGCCACGTTCGCCGGCTTTCCCCGTCAA
GCTCTAAATYSGGGGGCTCCCTTTAAGGTCCGATTAGTGCCTTACGCACCCTCGACCCMAAAAAACTKGAATTAAGGGGG
KGRATAGT
>P10.C1
ACYYTKKRMGGTWAWACCATCTTGTGCCTCAACATAAGATTGTCCACCCAGAATTGCTGGCAAGAGTCCAATTGCAAATT
TGACTTTCTCTGGCCAAGTAAGCATGTCATTATTCTTCMAGATGGCCCATATTCCTGCAAGACCAATTTGAACACAATAT
CCACATAAAAACAAGTTACTTCATTTGAAATTTGGCTGCACAAATTAGCTCACCATTTAATGGTGCTGGTAGCACCTCAG
GAAAATCAAATCGACTGAACTCTCCAGGCTTATTTGGCATTGCAAATATCATTGAATGCTCCTTCCACTGGCAACCGATC
ATTAATTCCTAGTTCTCCAAACAGGTTCTGCATATTTGGGTAAGCCCCAACTTAAGGTGGATCAAGAATCAATGTCACAA
GTTATTTGCTGCATCATAATGTATCTTAAAAAGAAACAAGAAGAATCATCTACATTCATGCTCATAAGAAATTCAAAAAA
GCACGGAACAAGATAAGGCAAAAATCGAATTCCCGCGGCCGCCATGGCGGCCGGGAGCATGCGACGTCGGGCCCAATTCG
CCCTATAGTGAGTCWTATTACAATTCACTGGCCGTCGTTTTACAACGTCATGACTGGGAAAACCCTGGCATTACCCAACT
TAAT
>P10.C2
CGTAAAAACASGAKGGGGARKGTTCTCTGGCCAAGTAAGCATGTCATTATTCTCCCAARAWAGGCCCATATTCCTGCAAG
ACCAATTTGAACACAATATCCACATAAAAACAAGTTACTTCATTTGAAATTTGGCTGCACAAATTASCTCACCATTTAAT
GGTGCTGGTAGCACCTCAGGAAAATCAAATCGACTGAACTCTCCAGGCTTATTTGGCATTGCAAATATCATTGAATGCTC
CTTCCACTGGCAACCGATCATTAATTCCTAGTTCTCCAAACAGGTTCTGCATATTTGGGTAAGCCCCAACTTAAGGTGGA
TCAAGAATCAATGTCACAAGTTATTTGCTGCATCATAATGTATCTTAAAAAGAAACAAGAAGAATCATCTACATTCATGC
TCATAAGAAATTCAAAAAAGCACGGAACAAGATAAGGCAAATAATCGAATTCCCGCGGCCGCCATGGCGGCCGGGAGCAT
GCGACGTCGGGCCCAATTCGCCCTATAGTGAGTCGTATTACAATTCACTGGCCGTCGTTTTACAACGTCGTGACTGGGAA
AACCCTGGCGTTACCCAACTTAATCGCCTTGCAGCACATCCCCCTTTCGCCAGCTGGCGTAAATAGCGAAGAGGCCCGCA
CCGATCGCCCTTCCCAACAGTTGCGCAGCCTGAATGGCGAAWGGACGCGCCCTGTAGCGGCGCATTAAGCGCGGCGGGGT
GTGGTGGTTACGCGCAGCGTGACCGCTACACTTGCCAGCGCCCTAGCGCCCGCTCTTTCGCTTTCTCCCTTCTTTCTCGC
CASTTYGCCGGCTTYCCCGTCAARCTMTAAATCGGGGGCTTCC
>P10.C3
TMRGTTWWCCATCTTGTGCCTCAACATAAGATTGTCCACCCAGAATTGCTGGCAAGAGTCCAATTGCAAATTTGACTTTC

TCTGGCCAAGTAAGCATGTCATTATTCTTCAAGATGGCCCATATTCCTGCAAGACCAATTTGAACACAATATCCACATAA
AAACAAGTTACTTCATTTGAAATTTGGCTGCACAAATTAGCTCACCATTTAATGGTGCTGGTAGCACCTCAGGAAAATCA
AATCGACTGAACTCTCCAGGCTTATTTGGCATTGCAAATATCATTGAATGCTCCTTCCACTGGCAACCGATCATTAATTC
CTAGTTCTCCAAACAGGTTCTGCATATTTGGGTAAGCCCCAACTTAAGGTGGATCAAGAATCAATGTCACAAGTTATTTG
CTGCATCATAATGTATCTTAAAAAGAAACAAGAAGAATCATCTACATTCATGCTCATAAGAAATTCAAAAAAGCACGGAA
CAAGATAAGGCAAATAATCGAATTCCCGCGGCCGCCATGGCGGCCGGGAGCATGCGACGTCGGGCCCAATTCGCCCTATA
GTGAGTCGTATTACAATTCACTGGCCGTCGTTTTACAACGTCGTGACTGGGAAAACCCTGGCGTTACCCAACTTAATCGC
CTTGCAGCACATCCCCCTTTCGCCAGYTGGCGTAAATAAKCGAA
>P10.C5
GACGKGGTWWCMTCTTGTGCCTCAACATAAGATTGTCCACCCAGAATTGCTGGCAAGAGTCCAATTGCAAATTTGACTTT
CTCTGGCCAAGTAAGCATGTCATTATTCTTCAAGATGGCCCATATTCCTGCAAGACCAATTTGAACACAATATCCACATA
AAAACAAGTTACTTCATTTGAAATTTGGCTGCACAAATTAGCTCACCATTTAATGGTGCTGGTAGCACCTCAGGAAAATC
AAATCGACTGAACTCTCCAGGCTTATTTGGCATTGCAAATATCATTGAATGCTCCTTCCACTGGCAACCGATCATTAATT
CCTAGTTCTCCAAACAGGTTCTGCATATTTGGGTAAGCCCCAACTTAAGGTGGATCAAGAATCAATGTCACAAGTTATTT
GCTGCATCATAATGTATCTTAAAAAGAAACAAGAAGAATCATCTACATTCATGCTCATAAGAAATTCAAAAAAGCACGGA
ACAAGATAAGGCAAATAATCGAATTCCCGCGGCCGCCATGGCGGCCGGGAGCATGCGACGTCGGGCCCAATTCGCCCTAT
AGTGAGTCGTATTACAATTCACTGGCCGTCGTTTTACAACGTCGTGACTGGGAAAACCCTGGCGTTACCCAACTTAATCG
CCTTGCAGCACATCCCCCTTTCGCCAGCTGGCGTAATAGCGAAGAGGCCCGCACCGATCGCCCTTCCCAACAGTTGCGCA
GCCTGAATGGCGAATGGACGCGCCCTGTAGCGGCGCATTAAGCGCGGCGGGTGTGGTGGTTACGCGCAGCGTGACCGCTA
CACTTGCCAGCGCCCTAGCGCCCGCTCCTTTCGCTTTCTTCCCTTCCTTTCTCGCCACGTTCGCGGCTTTCCCCGTCAAG
CTCTAAATCGGGGGGGCCTYCCCTTTTAGGGGGTTTCCCGAT
>P2.C2
GKGGSSCTTCAACMGTRAAGATTGTCCACCCRGAATTGCTGGCAAGAGTCCAATTGCAAATTTGACTTTCTCTGGCCAAG
TAAGCATGTCATTATTCTTCAAGATGGCCCATATTCCTGCAAGACCAATTTGAACACAATATCCACATAAAAACAAGTTA
CTTCATTTGAAATTTGGCTGCACAAATTAGCTCACCATTTAATGGTGCTGGTAGCACCTCAGGAAAATCAAATCGACTGA
ACTCTCCAGGCTTATTTGGCATTGCAAATATCATTGAATGCTCCTTCCACTGTCAACCGATCATTAATTCCTAGTTCTCC
AAACAGGTTCTGCATATTTGGGTAASCCCCAACTTAAGGTGGATCAAGAATCAATGTCACAAGTTATTTGCTGCATCATA
ATGTATCTTAAAAAGAAACAAGAAGAATCATCTACATTCATGCTCATAAGAAATTCAAAAAAGCACGGAACAAGATAAGG
CAAATAATCGAATTCCCGCGGCCGCCATGGCGGCCGGGAGCATGCGACGTCGGGCCCAATTCGCCCTATAGTGAGTCGTA
TTACAATTCACTGGCCGTCGTTTTACAACGTCGTGACTGGGAAAACCCTGGCGTTACCCAACTTAATCGCCTTGCAGCAC
ATCCCCCTTTCGCCAGCTGGCGTAATAGCGAAGAGGCCCGCACCGATCGCCCTTCCCAACAGTTGCGCAGCCTGAATGGC
GAATGGACGCGCCCTGTAGCGGCGCATTAAGCGCGGCGGGTGTGGTGGTTACGCGCAGCGTGACCGCTACACTTGCCAGC
GCCCTAGCGCCCGCTCCTTTCGCTTTCTTCCCTTCCTTTCTCGCCACGTTCGCCGGCTTTCCCGTCAAGCTCTAAATCGG
GGGCTCCTTTWAGGTTTCGATTTAGTGCTTACGGCACTCGACCCCAAACTGATAGGGTGAATGGTCACGTAGKGGGCCAY
GCCCCCGKAGWAR
>P2.C3
TGTTCTTTKGGKTGGCCTCAACATAAGATTGTCCACCCAGAATTGCTGGCAAGAGTCCAATTGCMWATTTGACTTTCTCT
GGCCAAGTAAGCATGTCATTATTCTTCAAGATGGCCCATATTCCTGCAAGACCAATTTGAACACAATATCCACATAAAAA
CAAGTTACTTCATTTGAAATTTGGCTGCACAAATTAGCTCACCATTTAATGGTGCTGGTAGCACCTCAGGAAAATCAAAT
CGACTGAACTCTCCAGGCTTATTTGGCATTGCAAATATCATTGAATGCTCCTTCCACTGACAACCGATCATTAATTCCTA
GTTCTCCAAACAGGTTCTGCATATTTGGGTAAGCCCCAACTTAAGGTGGATCAAGAATCAATGTCACAAGTTATTTGCTG
CATCATAATGTATCTTAAAAAGAAACAAGAAGAATCATCTACATTCATGCTCATAAGAAATTCAAAAAAGCACGGAACAA
GATAAGGCAAATAATCGAATTCCCGCGGCCGCCATGGCGGCCGGGAGCATGCGACGTCGGGCCCAATTCGCCCTATAGTG
AGTCGTATTACAATTCACTGGCCGTCGTTTTACAACGTCGTGACTGGGAAAACCCTGGCGTTACCCAACTTAATCGCCTT
GCAGCACATCCCCCTTTCGCCAGCTGGCGTAATAGCGAAGAGGCCCGCACCGATCGCCCTTCCCAACAGTTGCGCAGCCT
GAATGGCGAATGGACGCGCCCTGTAGCGGCGCATTAAGCGCGGCGGGTGTGGTGGTTACGCGCAGCGTGACCGCTACACT
TGCCAGCGCCCTAGCGCCGCTCCTTTCGCTTTCTTCCCTTTCTTTCTCGCACGTTCGCGGCTTTCCCGTCAAGCTCTAAT
CGGGGGCTCCTTTAGGGTCGATTAGTGCTTACGGCACTCGACCAAAAACTGATAGGTGATGGTTCASGTARTGTGGGCCA
TCSCCCACGT
>P2.C4
AWWRRRWKSYWMYRMRMMSTWWRRAYTKGSGTTATWCMTCTTGTGCCTCAACATAAGATTGTCCACCCAGAATTGCTGGC
AAGAGTCCAATTGCWWWTTTGACTTTCTCTGGCCAAGTAAGCATGTCATTATTCTTCAAGATGGCCCATATTCCTGCAAG
ACCAATTTGAACACAATATCCACATAAAAACAAGTTACTTCATTTGAAATTTGGCTGCACAAATTAGCTCACCATTTAAT
GGTGCTGGTAGCACCTCAGGAAAATCAAATCGACTGAACTCTCCAGGCTTATTTGGCATTGCAAATATCATTGAATGCTC
CTTCCACTGACAACCGATCATTAATTCCTAGTTCTCCAAACAGGTTCTGCATATTTGGGTAAGCCCCAACTTAAGGTGGA
TCAAGAATCAATGTCACAAGTTATTTGCTGCATCATAATGTATCTTAAAAAGAAACAAGAAGAATCATCTACATTCATGC
TCATAAGAAATTCAAAAAAGCACGGAACAAGATAAGGCAAATAATCGAATTCCCGCGGCCGCCATGGCGGCCGGGAGCAT

GCGACGTCGGGCCCAATTCGCCCTATAGTGAGTCGTATTACAATTCACTGGCCGTCGTTTTACAACGTCGTGACTGGGAA
AACCCTGGCGTTACCCAACTTAATCGCCTTGCARCACATCCCCCTTTCGCCAGCTGGCGTAATAGCGAAGAGGCCCGCAC
CGATCGCCCTTCCCAACAGTTGCGCAGCCTGAATGGCGAATGGACGCGCCCTGTAGCGGCGCATTAAGCGCGGCGGGTGT
GGTGGTTACGCGCAGCGTGACCGCTACACTTGCAGCGCCCTAGCGCCGCTCCTTCGCTTCTTCCTTCCTTTYYCGCCMGT
TCGCGGCTTTCCCGTCAGCTCTAAATCGGGGCTCGTTTAGGATCGAATTTATGGCTTACGTACCTCGACCCCMAAAACTK
GATATATCGG
>P3.C1
TMMAAMATAAAGAATTGTCCCACCCAGAATTGCTGGCAAGAGTCCAATTGCAAATTTGACTTTCTCTGGCCAAGTAAGCA
TGTCATTATTCTTCAAGATGGCCCATATTCCTGCAAGACCAATTTGAACACAATATCCACATAAAAACAAGTTACTTCAT
TTGAAATTTGGCTGCACAAATTAGCTCACCATTTAATGGTGCTGGTAGCACCTCARGAAAATCAAATCRACTGAACTCTC
CAGGCTTATTTGGCATTGCAAATATCATTGAATGCTCCTTCCACTGTCAACCGATCATTAATTCCTAGTTCTCCAAACAG
GTTCTGCATAWTTGGGTAARCCCCAACTTAAGGTGGAT
>P3.C2
GWGGCCCMWMACATAAARATTGTCCACCCAGAAWTGCTGGCAAGAGTCCAATTGCWWATTTGACTTTCTCTGGCCAAGTA
AGCATGTCATTATTCYTCAARATGGCCCATATTCCTGCAAGACCAATTTGAACACAATATCCACATAAAAACAAGTTACT
TCATTTGAAATTTGGCTGCACAAATTAGCTCACCATTTAATGGTGCWGGTASCACCTCAGGAAAATCAAATCAACWGA
>P3.C3
TTKGGTKGGCCTCAACATAAGGATTGTCCACCCAGAATTGCTGGCAAGAGTCCAATTGCAAATTTGACTTTCTCTGGCCA
AGTAAGCATGTCATTATTCTTCAAGATGGCCCATATTCCTGCAAGACCAATTTGAACACAATATCCACATAAAAACAAGT
TACTTCATTTGAAATTTGGCTGCACAAATTAGCTCACCATTTAATGGTGCTGGTAGCACCTCAGGAAAATCAAATCGACT
GAACTCTCCAGGCTTATTTGGCATTGCAAATATCATTGAATGCTCCTTCCACTGTCAACCGATCATTAATTCCTAGTTCT
CCAAACAGGTTCTGCATATTTGGGTAASCCCCAACTTAAGGTGGATCAAGAATCAATGTCACAAGTTATTTGCTGCATCA
TAATGTATCTTAAAAAGAAACAAGAAGAATCATCTACATTCATGCTCATAAGAAATTCAAAAAAGCACGGAACAAGATAA
GGCAAATAATCGAATTCCCGCGGCCGCCATGGCGGCCGGGAGCATGCGACGTCGGGCCCAATTCGCCCTATAGTGAGTCG
TATTACAATTCACTGGCCGTCGTTTTACAACGTCGTGACTGGGAAAACCCTGGCGTTACCCAACTTAATCGCCTTGCAGC
ACATCCCCCTTTCGCCAGCTGGCGTAATAGCGAAGAGGCCCGCACCGATCGCCCTTCCCAACAGTTGCGCAGCCTGAATG
GCGAATGGACGCGCCCTGTAGCGGCGCATTAAGCGCGGCGGGTGTGGTGGTTACGCGCAGCGTGACCGCTACACTTGCCA
GCGCCCTAGCGCCGCTCCTTTCGCTTTCTTCCCTTCCTTTCTCGCACGTTCGCGGCTTTCCCGTCAAGCTCTAAATCGGG
GGGCTCCTTWAGGATYCGATTARTGCTTTACGGCACTCGAACCCAGAACTGGATTAGGGTGATGTTMMGTAAGTGGCACT
CGCCCGTGTATK
>P3.C4
TCCCSSMRYRYYTTTKWMRTTWTMCATCTTGTGCCTCAACATAAAGATTGTCCACCCAGAATTGCTGGCAAGAGTCCAAT
TGCAAATTTGACTTTCTCTGGCCAAGTAAGCATGTCATTATTCTTCAAGATGGCCCATATTCCTGCAAGACCAATTTGAA
CACAATATCCACATAAAAACAAGTTACTTCATTTGAAATTTGGCTGCACAAATTAGCTCACCATTTAATGGTGCTGGTAG
CACCTCAGGAAAATCAAATCGACTGAACTCTCCAGGCTTATTTGGCATTGCAAATATCATTGAATGCTCCTTCCACTGTC
AACCGATCATTAATTCCTAGTTCTCCAAACAGGTTCTGCATATTTGGGTAAGCCCCAACTTAAGGTGGATCAAGAATCAA
TGTCACAAGTTATTTGCTGCATCATAATGTATCTTAAAAAGAAACAAGAAGAATCATCTACATTCATGCTCATAAGAAAT
TCAAAAAAGCACGGAACAAGATAAGGCAAATAATCGAATTCCCGCGGCCGCCATGGCGGCCGGGAGCATGCGACGTCGGG
CCCAATTCGCCCTATAGTGAGTCGTATTACAATTCACTGGCCGTCGTTTTACAACGTCGTGACTGGGAAAACCCTGGCGT
TACCCAACTTAATCGCCTTGCAGCACATCCCCCTTTCGCCAGCTGGCGTAATAGCGAAGAGGCCCGCACCGATCGCCCTT
CCCAACAGTTGCGCAGCCTGAATGGCGAATGGACGCGCCCTGTAGCGGCGCATTAAGCGCGGCGGGTGTGGTGGTTACGC
GCAGCGTGACCGCTACACTTGCCAGCGCCCTAGCGCCCGCTCCTTTCGCTTTCTTCCCTTTCTTTCTCGCACGTTCGCCG
GCTTTCCCCGTCAGCTCTAATCGGGGGCTCCTTTAGATCGATTAGTGCTTACGCACCTCGAACCCAAAAACTTGATTAGG
KKAAKGTTTMACG
>P3.C5
TAAACKKMSTWWWCMATCTTGTGCCTCAACATAAGATTGTCCACCCAGAATTGCTGGCAAGAGTCCAATTGCAAATTTGA
CTTTCTCTGGCCAAGTAAGCATGTCATTATTCTTCAAGATGGCCCATATTCCTGCAAGACCAATTTGAACACAATATCCA
CATAAAAACAAGTTACTTCATTTGAAATTTGGCTGCACAAATTAGCTCACCATTTAATGGTGCTGGTAGCACCTCAGGAA
AATCAAATCGACTGAACTCTCCAGGCTTATTTGGCATTGCAAATATCATTGAATGCTCCTTCCACTGTCAACCGATCATT
AATTCCTAGTTCTCCAAACAGGTTCTGCATATTTGGGTAAGCCCCAACTTAAGGTGGATCAAGAATCAATGTCACAAGTT
ATTTGCTGCATCATAATGTATCTTAAAAAGAAACAAGAAGAATCATCTACATTCATGCTCATAAGAAATTCAAAAAAGCA
CGGAACAAGATAAGGCAAAAATCGAATTCCCGCGGCCGCCATGGCGGCCGGGAGCATGCGACGTCGGGCCCAATTCGCCC
TATAGTGAGTCGTATTACAATTCACTGGCCGTCGTTTTACAACGTCGTGACTGGGAAAACCCTGGCGTTACCCAACTTAA
TCGCCTTGCAGCACATCCCCCTTTYGCCAGCTGGCGTAATAGCGAAGAGGCCCGCACCGATCGCCCTTCCCAACAGTTGC
GCAGCCTGAATGGGCGAATGGACGCGCCCTGTAGCGGCGCATTAAGCGCGGSGGGGTGTGGTGGTTACGCGCAGCGTGAC
CRCTAMACTTGCCAGCGCCCTAGCGCCCGCTYCTTTCGCTTTCATCMCGTTCCTTTTCTTCSCCACGATCTCRYCCGGMC
TMTCCCCGGAT

>P5.C1
GTTCCCMMCCCCCAGGAAATTGCTGGCAAGAGTCCAATTYSCWWWTTTGACTTTCTCTGGCCAAGTAAGCATGTCATTAT
TCTTCAAGATGGCCCATATTCCTGCAAGACCAATTTGAACACAATATCCACATAAAAACAAGTTACTTCATTTGAAATTT
GGCTGCACAAATTAGCTCACCATTTAATGGTGCTGGTAGCACCTCAGGAAAATCAAATCGACTGAACTCTCCAGGCTTAT
TTGGCATTGCAAATATCATTGAATGCTCCTTCCACTGAACCGATCATTAATTCCTAGTTCTCCAAACAGGTTCTGCATAT
TTGGGTAAGCCCCAACTTAAGGTGGATCAAGAATCAATGTCACAAGTTATTTGCTGCATCATAATGTATCTTAAAAAGAA
ACAAGAAGAATCATCTACATTCATGCTCATAAGAAATTCAAAAAAGCACGGAACAAGATAAGGCAAATAATCGAATTCCC
GCGGCCGCCATGGCGGCCGGGAGCATGCGACGTCGGGCCCAATTCRCCCTATAGTGAGTCGTATTACAATTCACTGGCCG
TCGTTTTACAACGTCGTGACTGGGAAAACCCTGGCGTTACCCAACTTAATCGCCTTGCAGCACATCCCCCTTTCGCCAGC
TGGCGTAATAGCGAAGAGGCCCGCACCGATCGCCCTTCCCAACAGTTGCGCACCTGAATGGCGAATGGACGCSCCCTGTA
SCGGCGCATTTAAKCGCGGCGGGTGWGGTGGTTACGCGCAGCGTGACCGCTACCTTGCAGCGCCCTAACCMCCGCTCCTT
TTASCTTT
>P5.C4
GATWWWCCMTCTTGTGCCTCAACATAAGATTGTCCACCCAGAATTGCTGGCAAGAGTCCAATTGCAAATTTGACTTTCTC
TGGCCAAGTAAGCATGTCATTATTCTTCAAGATGGCCCATATTCCTGCAAGACCAATTTGAACACAATATCCACATAAAA
ACAAGTTACTTCATTTGAAATTTGGCTGCACAAATTAGCTCACCATTTAATGGTGCTGGTAGCACCTCAGGAAAATCAAA
TCGACTGAACTCTCCAGGCTTATTTGGCATTGCAAATATCATTGAATGCTCCTTCCACTGAACCGATCATTAATTCCTAG
TTCTCCAAACAGGTTCTGCATATTTGGGTAAGCCCCAACTTAAGGTGGATCAAGAATCAATGTCACAAGTTATTTGCTGC
ATCATAATGTATCTTAAAAAGAAACAAGAAGAATCATCTACATTCATGCTCATAAGAAATTCAAAAAAGCACGGAACAAG
ATAAGGCAAATAATCGAATTCCCGCGGCCGCCATGGCGGCCGGGAGCATGCGACGTCGGGCCCAATTCGCCCTATAGTGA
GTCGTATTACAATTCACTGGCCGTCGTTTTACAACGTCGTGACTGGGAAAACCCTGGCGTTACCCAACTTAATCGCCTTG
CAGCACATCCCCCTTTCGCCAGCTGGCGTAAATAGCGAAGAGGCCCGCACCGATCGCCCTTCCCMAACAGTTGCGCAGCC
TGAATGGCGAATGGACGCGCCCTGTAGCGGCGCATWAAGCGCGGCGGGTGTGGTGGTTACGCGCAGCGKGACCGCTACAC
TTGCCARCGCCCTTASCGCCMGCTCCMTTTCGMTTTCTTCCCTTCCTWTTCTCGCCACSKTYCGCSGGCTTTCCCCGWCA
AGCTCTAAATCGGGGGGCTCCYWTTAGGGTCCGAATTAGTGSTTTTACGGCACSCTYSTACCCCCMAAAAAAACATTGRA
ATTAKRGRKGGAGAATGAKGATYCATACGRMMKATASTS
>P5.C5
ATTWRCTWWCMTCTTGTGCCTCACATAAGATTGTCCACCCAGAATTGCTGGCAAGAGTCCAATTGCAAATTTGACTTTCT
CTGGCCAAGTAAGCATGTCATTATTCTTCAAGATGGCCCATATTCCTGCAAGACCAATTTGAACACAATATCCACATAAA
AACAAGTTACTTCATTTGAAATTTGGCTGCACAAATTAGCTCACCATTTAATGGTGCTGGTAGCACCTCAGGAAAATCAA
ATCGACTGAACTCTCCAGGCTTATTTGGCATTGCAAATATCATTGAATGCTCCTTCCACTGAACCGATCATTAATTCCTA
GTTCTCCAAACAGGTTCTGCATATTTGGGTAAGCCCCAACTTAAGGTGGATCAAGAATCAATGTCACAAGTTATTTGCTG
CATCATAATGTATCTTAAAAAGAAACAAGAAGAATCATCTACATTCATGCTCATAAGAAATTCAAAAAAGCACGGAACAA
GATAAGGCAAATAATCGAATTCCCGCGGCCGCCATGGCGGCCGGGAGCATGCGACGTCGGGCCCAATTCGCCCTATAGTG
AGTCGTATTACAATTCACTGGCCGTCGTTTTACAACGTCGTGACTGGGAAAACCCTGGCGTTACCCAACTTAATCGCCTT
GCAGCACATCCCCCTTTCGCCAGCTGGCGTAATAGCGAAGAGGCCCGCACCGATCGCCCTTCCCAACAGTTGCGCAGCCT
GAATGGCGAATGGACGCGCCCTGTAGCGGCGCATTAAGCGCGGCGGGTGTGGTGGTTACGCGCAGCGTGACCGCTACACT
TGCCAGCGCCCTAGCGCCCGCTCCTTTCGCTTTCTTCCCTTCCTTTCTCGCCACGTCGCCGGCTTCCCCGTCAAGCTCTA
AATCGGGGGCTTCCCTTTAGGATTCCGGAATTTWRKKTGCCT
>P5.C6
AMCCKKWMRGTWAWMCATCTTGTGCCTCAACATAAGATTGTCCACCCAGAATTGCTGGCAAGAGTCCAATTGCAAATTTG
ACTTTCTCTGGCCAAGTAAGCATGTCATTATTCTTCAAGATGGCCCATATTCCTGCAAGACCAATTTGAACACAATATCC
ACATAAAAACAAGTTACTTCATTTGAAATTTGGCTGCACAAATTAGCTCACCATTTAATGGTGCTGGTAGCACCTCAGGA
AAATCAAATCGACTGAACTCTCCAGGCTTATTTGGCATTGCAAATATCATTGAATGCTCCTTCCACTGAACCGATCATTA
ATTCCTAGTTCTCCAAACAGGTTCTGCATATTTGGGTAAGCCCCAACTTAAGGTGGATCAAGAATCAATGTCACAAGTTA
TTTGCTGCATCATAATGTATCTTAAAAAGAAACAAGAAGAATCATCTACATTCATGCTCATAAGAAATTCAAAAAAGCAC
GGAACAAGATAAGGCAAATAATCGAATTCCCGCGGCCGCCATGGCGGCCGGGAGCATGCGACGTCGGGCCCAATTCGCCC
TATAGTGAGTCGTATTACAATTCACTGGCCGTCGTTTTACAACGTCGTGACTGGGAAAACCCTGGCGTTACCCAACTTAA
TCGCCTTGCAGCACATCCCCCTTTCGCCAGCTGGCGTAATAGCGAAGAGGCCCGCACCGATCGCCCTTCCCAACAGTTGC
GCAGCCTGAATGGCGAATGGACGCGCCCTGTAGCGGCGCATTAAGCGCGGCGGGTGTGGTGGTTACGCGCAGCGTGACCG
CTACACTTGCCAGCGCCCTAGCGCCCGCTCCTTTCGCTTTCTTCCCTTCCTTTTCTCGCCACGTTCGCGGCTTCCCGTCA
GCTCTAAATCGGGGGCTTCCCTTTAGGGCTTCCCRATTTTAGTKGCTTTTTACCGSGCG
>P6.C1
ATYKKKRMGGTTWWCMTTCTTGKGCCTCAACATAAGATTGTCCACCCAGAATTGCTGGCAAGAGTCCAATTGCAAATTTG
ACTTTCTCTGGCCAAGTAAGCATGTCATTATTCTTCAAGATGGCCCATATTCCTGCAAGACCAATTTGAACACAATATCC
ACATAAAAACAAGTTACTTCATTTGAAATTTGGCTGCACAAATTAGCTCACCATTTAATGGTGCTGGTAGCACCTCAGGA
AAATCAAATCGACTGAACTCTCCAGGCTTATTTGGCATTGCAAATATCATTGAATGCTCCTTCCACTGCCAACCGATCAT

TAATTCCTAGTTCTCCAAACAGGTTCTGCATATTTGGGTAAGCCCCAACTTAAGGTGGATCAAGAATCAATGTCACAAGT
TATTTGCTGCATCATAATGTATCTTAAAAAGAAACAAGAAGAATCATCTACATTCATGCTCATAAGAAATTCAAAAAAGC
ACGGAACAAGATAAGGCAAATAATCGAATTCCCGCGGCCGCCATGGCGGCCGGGARCATGCGACGTCGGGCCCAATTCGC
CCTATAGTGAGTCGTATTACAATTCACTGGSCGTCGTTTTACAACGTCGTGACTGGGAAAACCCTGGCGTTACCCAACTT
AATCGCCTTGCAGCACATCCCCCTTTCGCCAGCTGGCGTAATARCGAAGAGGCCCGCACCGATCGCCCTTCCCAACAGTT
GCGCAGCCTGAATGGSGRAWGGRCGCGSCCCTGTAGCGGCGCAYTWAAGCGCGGCGGGTGTGGKGGGTWACGMGCARSGT
GACCGCTMCACTTGCCAGCGCCCTARCGSMCGCTCCCTTSGCTTTTCTCCCTTWCTTTTCTTSSCCASTTCGCCGGSYTT
CCCMSTCAAGCTCTAAAWCCGGGGGCGCCCTTTAGGGTTCCTGATTWATGTGSTRTAYGMGCA
>P6.C2
CSWGTKKKYRRWWWWMCCMMTCYTTGTGCCTCACATAAGATTGTCCACCCAGAATTGCTGGCAAGAGTCCAATTGCMAAT
TTGACTTTCTCTGGCCAAGTAAGCATGTCATTATTCTTCAMGATGGCCCATATTCCTGCAAGACCAATTTGAACACAATA
TCCACATAAAAACAAGTTACTTCATTTGAAATTTGGCTGCACAAATTAGCTCACCATTTAATGGTGCTGGTAGCACCTCA
GGAAAATCAAATCGACTGAACTCTCCAGGCTTATTTGGCATTGCAAATATCATTGAATGCTCCTTCCACTGCCAACCGAT
CATTAATTCCTAGTTCTCCAAACAGGTTCTGCATATTTGGGTAAGCCCCAACTTAAGGTGGATCAAGAATCAATGTCACA
AGTTATTTGCTGCATCATAATGTATCTTAAAAAGAAACAAGAAGAATCATCTACATTCATGCTCATAAGAAATTCAAAAA
AGCACGGAACAAGATAAGGCAAAAATCGAATTCCCGCGGCCGCCATGGCGGCCGGGAGCATGCGACGTCGGGCCCAATTC
GCCCTATAGTGAGTCGTATTACAATTCACTGGCCGTCGTTTTACAACGTCGTGACTGGGAAAACCCTGGCGTTACCCAAC
TTAATCGCCTTGCAGCACATCCCCCTTTCGCCAGCTGGCGTAATAGCGAAGAGGCCCGCACCGATCGCCCTTCCCAACAG
TTGCGCAGCCTGAATGGCGAATGGACGCGCCCTGTAGCGGCGCATTAAGCGCGGCGGGTGTGGTGGTTACGCGCAGCGTG
ACCGCTACACTTGCCAGCGCCCTAGCGCCCGCTCCTTTCGCTTTCTTCCCTTCCTTTCTCGCCACGTTCGCCGGCTTTCC
CGTCAAGCTCTAAATCGGGGGGCTYCCTTTAGGATCGAATTARTGCTTTACGCACCTCGACCCCAAAAAACCTKGAATTA
GGTGARATAGGCTA
>WT1.C1
GCTKKARGTWWWCMATCTTGTGCCTCAACATAAGATTGTCCACCCAGAATTGCTGGCAAGAGTCCAATTGCAAATTTGAC
TTTCTCTGGCCAAGTAAGCATGTCATTATTCTTCAAGATGGCCCATATTCCTGCAAGACCAATTTGAACACAATATCCAC
ATAAAAACAAGTTACTTCATTTGAAATTTGGCTGCACAAATTAGCTCACCATTTAATGGTGCTGGTAGCACCTCAGGAAA
ATCAAATCGACTGAACTCTCCAGGCTTATTTGGCATTGCAAATATCATTGAATGCTCCTTCCACTGCAACCGATCATTAA
TTCCTAGTTCTCCAAACAGGTTCTGCATATTTGGGTAAGCCCCAACTTAAGGTGGATCAAGAATCAATGTCACAAGTTAT
TTGCTGCATCATAATGTATCTTAAAAAGAAACAAGAAGAATCATCTACATTCATGCTCATAAGAAATTCAAAAAAGCACG
GAACAAGATAAGGCAAATAATCGAATTCCCGCGGCCGCCATGGCGGCCGGGAGCATGCGACGTCGGGCCCAATTCGCCCT
ATAGTGAGTCGTATTACAATTCACTGGCCGTCGTTTTACAACGTCGTGACTGGGAAAACCCTGGCGTTACCCAACTTAAT
CGCCTTGCAGCACATCCCCCTTTCGCCAGCTGGCGTAATAGCGAAGAGGCCCGCACCGATCGCCCTTCCCAACAGTTGCG
CAGCCTGAATGGCGAATGGACGCGCCCTGTAGCGGCGCATTAAGCGCGGCGGGTGTGGTGGTTACGCGCAGCGTGACCGC
TACACTTGCCAGCGCCCTAGCGCCCGCTCCTTTCGCTTTCTTCCTTCTTTCTCGCCACGTTCGCCGGCTTTCCCGTCAGC
TCTAATCGGGGGGCTCCCTTAAGGGTTCCGATTAGTGCTTACGCACCTCGAACCCCAAAAAACTTGATTWGAGGGGGKGA
WGTGGG
>WT1.C2
CWRGTWWWCCATCTTGTGCCTCAACATAAGATTGTCCACCCAGAATTGCTGGCAAGAGTCCAATTGCAAATTTGACTTTC
TCTGGCCAAGTAAGCATGTCATTATTCTTCAAGATGGCCCATATTCCTGCAAGACCAATTTGAACACAATATCCACATAA
AAACAAGTTACTTCATTTGAAATTTGGCTGCACAAATTAGCTCACCATTTAATGGTGCTGGTAGCACCTCAGGAAAATCA
AATCGACTGAACTCTCCAGGCTTATTTGGCATTGCAAATATCATTGAATGCTCCTTCCACTGCAACCGATCATTAATTCC
TAGTTCTCCAAACAGGTTCTGCATATTTGGGTAAGCCCCAACTTAAGGTGGATCAAGAATCAATGTCACAAGTTATTTGC
TGCATCATAATGTATCTTAAAAAGAAACAAGAAGAATCATCTACATTCATGCTCATAAGAAATTCAAAAAAGCACGGAAC
AAGATAAGGCAAAAATCGAATTCCCGCGGCCGCCATGGCGGCCGGGAGCATGCGACGTCGGGCCCAATTCGCCCTATAGT
GAGTCGTATTACAATTCACTGGCCGTCGTTTTACAACGTCGTGACTGGGAAAACCCTGGCGTTACCCAACTTAATCGCCT
TGCAGCACATCCCCCTTTCGCCAGCTGGCGTAATAGCGAAGAGGCCCGCACCGATCGCCCTTCCCAACAGTTGCGCAGCC
TGAATGGCGAATGGACGCGCCCTGTAGCGGCGCATTAAGCGCGGCGGGTGTGGTGGTWACGCGCAGCGTGACCGCTAMAC
TTGCCAGCGCCCTAGCGCCCGCTCCTTTCRCTTTCTTCCCTTCCTTTCTCGCCACGTTCGCCAGCTTTCCCRGTCAAGCT
CTAAATCRGGGGCTCCCKTTAGGGTTTCGAATTAGTGCCTTTACGGCACCTCGAACCCAAAAAACTTGRATTAGGGGTGA
WGGGKTMTMACSARTAWG
>WT1.C3
ATTGTAAGKTAAACCMRTKCWWTGRAGCCTCAACATAAGATTGTCCACCCAGAATTGCTGGCAAGAGTCCAATTGCMAWT
TTGACTTTCTCTGGCCAAGTAAGCATGTCATTATTCTTCAAGATGGCCCATATTCCTGCAAGACCAATTTGAACACAATA
TCCACATAAAAACAAGTTACTTCATTTGAAATTTGGCTGCACAAATTAGCTCACCATTTAATGGTGCTGGTAGCACCTCA
GGAAAATCAAATCGACTGAACTCTCCAGGCTTATTTGGCATTGCAAATATCATTGAATGCTCCTTCCACTGCAACCGATC
ATTAATTCCTAGTTCTCCAAACAGGTTCTGCATATTTGGGTAAGCCCCAACTTAAGGTGGATCAAGAATCAATGTCACAA
GTTATTTGCTGCATCATAATGTATCTTAAAAAGAAACAAGAAGAATCATCTACATTCATGCTCATAAGAAATTCAAAAAA

GCACGGAACAAGATAAGGCAAATAATCGAATTCCCGCGGCCGCCATGGCGGCCGGGAGCATGCGACGTCGGGCCCAATTC
GCCCTATAGTGAGTCGTATTACAATTCACTGGCCGTCGTTTTACAACGTCGTGACTGGGAAAACCCTGGCGTTACCCAAC
TTAATCGCCTTGCAGCACATCCCCCTTTCGCCAGCTGGCGTAATAGCGAAGAGGCCCGCACCGATCGCCCTTCCCAACAG
TTGCGCAGCCTGAATGGCGAATGGACGCGCCCTGTAGCGGCGCATTAAGCGCGGCGGGTGTGGTGGTTACGCGCAGCGTG
ACCGCTACACTTGCCAGCGCCCTAGCGCCCGCTCCTTTCGCTTTCTCCCTTCCTTTCTCGCCACGTTCGCGGCTTTCCCG
TCAGCTCTAATCGGGGGCTCCCGTTAGGGTTCGATTAGTGCCTTAMCGCACCTCGACCCCAAAAACTTGAATTAGGGGTG
AATGGATTTA
>WT1.C4
ASKSKYKRSSGGKKRWWWCMMYYMCTKTGGKKGSCTCAACATAAGATTGTCCACCCAGAATTGCTGGCAAGAGTCCAATT
GCAAATTTGACTTTCTCTGGCCAAGTAAGCATGTCATTATTCTTCAAGATGGCCCATATTCCTGCAAGACCAATTTGAAC
ACAATATCCACATAAAAACAAGTTACTTCATTTGAAATTTGGCTGCACAAATTAGCTCACCATTTAATGGTGCTGGTAGC
ACCTCAGGAAAATCAAATCGACTGAACTCTCCAGGCTTATTTGGCATTGCAAATATCATTGAATGCTCCTTCCACTGCAA
CCGATCATTAATTCCTAGTTCTCCAAACAGGTTCTGCATATTTGGGTAAGCCCCAACTTAAGGTGGATCAAGAATCAATG
TCACAAGTTATTTGCTGCATCATAATGTATCTTAAAAAGAAACAAGAAGAATCATCTACATTCATGCTCATAAGAAATTC
AAAAAAGCACGGAACAAGATAAGGCAAATAATCGAATTCCCGCGGCCGCCATGGCGGCCGGGAGCATGCGACGTCGGGCC
CAATTCGCCCTATAGTGAGTCGTATTACAATTCACTGGCCGTCGTTTTACAACGTCGTGACTGGGAAAACCCTGGCGTTA
CCCAACTTAATCGCCTTGCAGCACATCCCCCTTTCGCCAGCTGGCGTAATAGCGAAGAGGCCCGCACCGATCGCCCTTCC
CAACAGTTGCGCAGCCTGAATGGCGAATGGACGCGCCCTGTAGCGGCGCATTAAGCGCGGCGGGTGTGGTGGTTACGCGC
AGCGTGACCGCTACACTTGCCAGCGCCCTAGCGCCCGCTCCTTTCGCTTTCTTCCCTTTCCTTTCTCGCMCGTTCGCCGG
CTTTCCCGTCAAGCTCTAAATCGGGGGGCTCCCTTTAGGGATCGATTTAGTGCCTTACGCACTCGACCCCAAAAAACTTG
AATTTAGGGGGTGGAAT
>WT2.C3
GGAAATTTGGKTCCCMCCCYCAGAATTTGCTGGCAAGAGTCCAATTGCAMCMYTTTKACTTTCTCTGGCCAAGTAAGCAT
GTCATTATTCTTCAASATGGCCCATATTCCTGCAAGACCAATTTGAACACAATATCCACATAAAAACAAGTTACTTCATT
TGAAATTTGGCTGCMCAAATTAGCTCACCATTTAATGGTGCTGGTAGCACCTCAGGAAAATCAAATCSACTGAACTCTCC
AGGCTTATTTGGCATTGCAAATATCATTGAATGCTCCTTCCACTGCAACCGATCATTAATTCCTAGTTCTCCAAACAGGT
TCTGCATATTTGGGTAAGCCCCAACTTAAGGTGGATCAAGAATCAATGTCACAAGTTATTTGCTGCATCATAATGTATCT
TAAAAAGAAACAAKAAGAATCATCTACATTCATGCTCATAAGAAATTCAAAAAAGCACGGAACAAGATAAGGCAAATAAT
CKAATTCCCGCGGCCGCCATGGCGGCCGGGAGCATGCGACGTCSGGCCCAATTCGCCCTATAKTGAGTCGTATTACAATT
TTCTGGCCGTCGTTTTACAACGTCGTGACTGGGAAAACCCTG
>WT2.C4
CCYTKKKMSRGTTTWWWCCMTTCTTGTGCCTCAACATAAGATTGTCCACCCAGAATTGCTGGCAAGAGTCCAATTGCAAA
TTTGACTTTCTCTGGCCAAGTAAGCATGTCATTATTCTTCAAGATGGCCCATATTCCTGCAAGACCAATTTGAACACAAT
ATCCACATAAAAACAAGTTACTTCATTTGAAATTTGGCTGCACAAATTAGCTCACCATTTAATGGTGCTGGTAGCACCTC
AGGAAAATCAAATCGACTGAACTCTCCAGGCTTATTTGGCATTGCAAATATCATTGAATGCTCCTTCCACTGCAACCGAT
CATTAATTCCTAGTTCTCCAAACAGGTTCTGCATATTTGGGTAAGCCCCAACTTAAGGTGGATCAAGAATCAATGTCACA
AGTTATTTGCTGCATCATAATGTATCTTAAAAAGAAACAAGAAGAATCATCTACATTCATGCTCATAAGAAATTCAAAAA
AGCACGGAACAAGATAAGGCAAATAATCGAATTCCCGCGGCCGCCATGGCGGCCGGGAGCATGCGACGTCGGGCCCAATT
CGCCCTATAGTGAGTCGTATTACAATTCACTGGCCGTCGTTTTACAACGTCGTGACTGGGAAAACCCTGGCGTTACCCAA
CTTAATCGCCTTGCAGCACATCCCCCTTTCGCCAGCTGGCGTAATAGCGAAGAGGCCCGCACCGATCGCCCTTCCCAACA
GTTGCGCAGCCTGAATGGCGAATGGACGCGCCCTGTAGCGGCGCATTAAGCGCGGCGGGTGTGGTGGTTACGCGCAGCGT
GACCGCTACACTTGCCAGCGCCCTAGCGCCCGCTCCTTTCGCTTTCTTCCCTTCCTTTCTCGCCACGTTCGCCGGCTTTC
CCGTCAAGCTCTAAATCGGGGGCTCCCTTTAGGTTCGAATTAGTGCTTTACGGCACCCTCGAACCCCAAAAAACCTTGGA
TTWAG
>WT2.C6
AMYTKKKSSGTTWWCCMATCCTTGTGCCTCAACATAAGATTGTCCACCCAGAATTGCTGGCAAGAGTCCAATTGCAAATT
TGACTTTCTCTGGCCAAGTAAGCATGTCATTATTCTTCAAGATGGCCCATATTCCTGCAAGACCAATTTGAACACAATAT
CCACATAAAAACAAGTTACTTCATTTGAAATTTGGCTGCACAAATTAGCTCACCATTTAATGGTGCTGGTAGCACCTCAG
GAAAATCAAATCGACTGAACTCTCCAGGCTTATTTGGCATTGCAAATATCATTGAATGCTCCTTCCACTGCAACCGATCA
TTAATTCCTAGTTCTCCAAACAGGTTCTGCATATTTGGGTAASCCCCAACTTAAGGTGGATCAAGAATCAATGTCACAAG
TTATTTGCTGCATCATAATGTATCTTAAAAAGAAACAAGAAGAATCATCTACATTCATGCTCATAAGAAATTCAAAAAAG
CACGGAACAAGATAAGGCAAATAATCACTAGTGAATTCGCGGCCGCCTGCAGGTCGACCATATGGGAGAGCTCCCAACGC
GTTGGATGCATAGCTTGAGTATTCTATAGTGTCACCTAAATAGCTTGGCGTAATCATGGTCATAGCTGTTTCCTGTGTGA
AATTGTTATCCGCTCACAATTCCACACAACATACGAGCCGGAAGCATAAAGTGTAAAGCCTGGGGTGCCTAATGAGTGAG
CTAACTCACATTAATTGCGTTGCGCTCACTGCCCGCTTTCCAGTCGGGAAACCTGTCGTGCCAGCTGCATTAATGAATCG
GCCAACGCGCGGGGAGAGGCGGTTTGCGTATTGGGCGCTCTTCGCTTCCTCGCTCACTGACTCGCTGCGCTCGGTCGTTC
GGCTGGCGGCGAGCGGTATCAGCTCACTCAAGGCGCTATACGGGCTATTCCACAGATCAGGCATACGCAGGAGAACATGG

KGAGGCCAAAAAGAGTGGATA
>G1.C1
GYTKKMSGTWWACMATCTTGTGCCTCAACATAAGATTGTCCACCCAGAATTGCTGGCAAGAGTCCAATTGCAAATTTGAC
TTTCTCTGGCCAAGTAAGCATGTCATTATTCTTCAAGATGGCCCATATTCCTGCAAGACCAATTTGAACACAATATCCAC
ATAAAAACAAGTTACTTCATTTGAAATTTGGCTGCACAAATTAGCTCACCATTTAATGGTGCTGGTAGCACCTCAGGAAA
ATCAAATCGACTGAACTCTCCAGGCTTATTTGGCATTGCAAATATCATTGAATGCTCCTTCCACTGCAACCGATCATTAA
TTCCTAGTTCTCCAAACAGGTTCTGCATATTTGGGTAAGCCCCAACTTAAGGTGGATCAAGAATCAATGTCACAAGTTAT
TTGCTGCATCATAATGTATCTTAAAAAGAAACAAGAAGAATCATCTACATTCATGCTCATAAGAAATTCAAAAAAGCACG
GAACAAGATAAGGCAAATAATCGAATTCCCGCGGCCGCCATGGCGGCCGGGAGCATGCGACGTCGGGCCCAATTCGCCCT
ATAGTGAGTCGTATTACAATTCACTGGCCGTCGTTTTACAACGTCGTGACTGGGAAAACCCTGGCGTTACCCAACTTAAT
CGCCTTGCAGCACATCCCCCTTTCGCCAGCTGGCGTAATAGCGAAGAGGCCCGCACCGATCGCCCTTCCCAACAGTTGCG
CAGCCTGAATGGCGAATGGACGCGCCCTGTAGCGGCGCATTAAGCGCGGCGGGTGTGGTGGTTACGCGCAGCGTGACCGC
TACACTTGCCAGCGCCCTAGCGCCCGCTCCTTTCGCTTTCTTCCCTTTCCTTTCTCGCCACGTTCGCCGGCTTTCCCGTC
AAGCTCTAATCGGGGGGCTCCTTTAGGATTCGAATTTAGTGCTTTACGGCACCTCGAACCCCCTAAAAAAACCATGKA
>G1.C2
GMYTYCTTKGTTGGSCCTMAMMATAAGATTGTCCACCCAGAATTGCTGGCAAGAGTCCAATTGCAAMYTTKACTTTCTCT
GGCCAAGTAAGCATGTCATTATTCTTCAAGATGGCCCATATTCCTGCAAGACCAATTTGAACACAATATCCACATAAAAA
CAAGTTACTTCATTTGAAATTTGGCTGCACAAATTAGCTCACCATTTAATGGTGCTGGTAGCACCTCAGGAAAATCAAAT
CGACTGAACTCTCCAGGCTTATTTGGCATTGCAAATATCATTGAATGCTCCTTCCACTGCAACCGATCATTAATTCCTAG
TTCTCCAAACAGGTTCTGCATATTTGGGTAAGCCCCAACTTAAGGTGGATCAAGAATCAATGTCACAAGTTATTTGCTGC
ATCATAATGTATCTTAAAAAGAAACAAGAAGAATCATCTACATTCATGCTCATAAGAAATTCAAAAAAGCACGGAACAAG
ATAAGGCAAATAATCGAATTCCCGCGGCCGCCATGGCGGCCGGGAGCATGCGACGTCGGGCCCAATTCGCCCTATAGTGA
GTCGTATTACAATTCACTGGCCGTCGTTTTACAACGTCGTGACTGGGAAAACCCTGGCGTTACCCAACTTAATCGCCTTG
CAGCACATCCCCCTTTCGCCAGCTGGCGTAATARCGAAGAGGCCCGCACCGATCGCCCTTCCCAACAGTTGCGCAGCCTG
AATGGCGAATGGACGCGCCCTGTAGCGGCGCATTAAGCGCGCGGGTGTGGTGGTTACGCGCAGCGTGACCGCTWMACTTG
CCAGCGCCCTAGCGCCCGCTCCTTTCGCTTTCCTTCCCTCCTTTCTCGCCACGTTCGCCGGCTTTCCCCGTCAAGCTCTA
AATCGGGGGGCYTCCCTTTTAGGTCCGAATTTARTGCGTTACGGCACTTCGACCCCCAAAATACTWGGAYATGACGGGCG
TGAMATC
>G1.C3
GTTWWMCCMWTTYCTTTGGTGGCCTCAACATAAGATTGTCCACCCAGAATTGCTGGCAAGAGTCCAATTGCAAATTTGAC
TTTCTCTGGCCAAGTAAGCATGTCATTATTCTTCAAGATGGCCCATATTCCTGCAAGACCAATTTGAACACAATATCCAC
ATAAAAACAAGTTACTTCATTTGAAATTTGGCTGCACAAATTAGCTCACCATTTAATGGTGCTGGTAGCACCTCAGGAAA
ATCAAATCGACTGAACTCTCCAGGCTTATTTGGCATTGCAAATATCATTGAATGCTCCTTCCACTGCAACCGATCATTAA
TTCCTAGTTCTCCAAACAGGTTCTGCATATTTGGGTAAGCCCCAACTTAAGGTGGATCAAGAATCAATGTCACAAGTTAT
TTGCTGCATCATAATGTATCTTAAAAAGAAACAAGAAGAATCATCTACATTCATGCTCATAAGAAATTCAAAAAAGCACG
GAACAAGATAAGGCAAATAATCGAATTCCCGCGGCCGCCATGGCGGCCGGGAGCATGCGACGTCGGGCCCAATTCGCCCT
ATAGTGAGTCGTATTACAATTCACTGGCCGTCGTTTTACAACGTCGTGACTGGGAAAACCCTGGCGTTACCCAACTTAAT
CGCCTTGCAGCACATCCCCCTTTCGCCAGCTGGCGTAATARCGAAGAGGCCCGCACCGATCGCCCTTCCCAAACAGTTGC
GCAGCCTGAATGGCGAATGGACGCGGCCCTGTAGCGGCGCATTAAGCGCGGGCGGGTGTGGTGGGTTACGCGCAGCGTGA
CCGCTACACTTGCAGCGCCCTAGCGCCCGCTCCTTTCGGCTTTCTCCTTCCTTCTCGCAMGTTCGCCGGCTTTCCCGTCA
AGCTCTAARTCGGGGGCTCCCTTTAGGGTCGAATTAGTGCTTACGGCACCTCGACCCMAAAAAACTKGAAATTAAGGGGG
GTGGAT
>G2.C1
CTKKWRRGKTWWWAMCMATCTTGTGCCTCAACATAAGATTGTCCACCCAGAATTGCTGGCAAGAGTCCAATTGCAAATTT
GACTTTCTCTGGCCAAGTAAGCATGTCATTATTCTTCAAGATGGCCCATATTCCTGCAAGACCAATTTGAACACAATATC
CACATAAAAACAAGTTACTTCATTTGAAATTTGGCTGCACAAATTAGCTCACCATTTAATGGTGCTGGTAGCACCTCAGG
AAAATCAAATCGACTGAACTCTCCAGGCTTATTTGGCATTGCAAATATCATTGAATGCTCCTTCCACTGCAACCGATCAT
TAATTCCTAGTTCTCCAAACAGGTTCTGCATATTTGGGTAAGCCCCAACTTAAGGTGGATCAAGAATCAATGTCACAAGT
TATTTGCTGCATCATAATGTATCTTAAAAAGAAACAAGAAGAATCATCTACATTCATGCTCATAAGAAATTCAAAAAAGC
ACGGAACAAGATAAGGCAAATAATCGAATTCCCGCGGCCGCCATGGCGGCCGGGAGCATGCGACGTCGGGCCCAATTCGC
CCTATAGTGAGTCGTATTACAATTCACTGGCCGTCGTTTTACAACGTCGTGACTGGGAAAACCCTGGCGTTACCCAACTT
AATCGCCTTGCAGCACATCCCCCTTTCGCCAGCTGGCGTAATAGCGAAGAGGCCCGCACCGATCGCCCTTCCCAACAGTT
GCGCAGCCTGAATGGCGAATGGACGCGCCCTGTAGCGGCGCATTAAGCGCGGCGGGTGTGGTGGTTACGCGCAGCGTGAC
CGCTACACTTGCCAGCGCCCTAGCGCCCGCTCCTTTCGCTTTCTTCCTTCCTTTCTCGCCACGTTCGCCGGCTTCCCGTC
AAGCTCTAAATCGGGGGCTCCCTTTAGGTTCGATTAGTGCTTACGCACCTCGAACCCCAAAAAAAACTTKGAATTWACGA
GT
>G2.C2

CCMAMAMMYYYTTTTWGAMCTTTYTCTCTMGGCCAAGTAAGCATGTCATTATTCTTCMMGATGGCCCATATTCCTGCAAG
ACCAATTTGAACACAATATCCACATAAAAACAAGTTACTTCATTTGAAATTTGGCTGCACAAATTAGCTCACCATTTAAT
GGTGCTGGTAGCACCTCAGGAAAATCAAATCGACTGAACTCTCCAGGCTTATTTGGCATTGCAAATATCATTGAATGCTC
CTTCCACTGCAACCGATCATTAATTCCTAGTTCTCCAAACAGGTTCTGCATATTTGGGTAAGCCCCAACTTAAGGTGGAT
CAAGAATCAATGTCACAAGTTATTTGCTGCATCATAATGTATCTTAAAAAGAAACAAGAAGAATCATCTACATTCATGCT
CATAAGAAATTCAAAAAAGCACGGAACAAGATAAGGCAAATAATCGAATTCCCGCGGCCGCCATGGCGGCCGGGAGCATG
CGACGTCGGGCCCAATTCGCCCTATAGTGAGTCGTATTACAATTCACTGGCCGTCGTTTTACAACGTCGTGACTGGGAAA
ACCCTGGCRTTACCCAACTTAATCGCCTTGCASCACATCCCCTTTCGCCAGCTGGCGTAATAKCGAAGAGGMCCGCACCG
ATCGCCCTTCCCACMGTTGCGCAGCCTGAATGGCGAAGGGACSCGCCCGTARCGGCGCATTAGGCGCGGCGGGWGTGG
>G2.C3
AKGGCCCCTYMAACAWTAARATTTGTCCACCCAGAATTGCTGGCAAGAGTCCAATTGCAAATTTGACTTTCTCTGGCCAA
GTAAGCATGTCATTATTCTTCAAGATGGCCCATATTCCTGCAAGACCAATTTGAACACAATATCCACATAAAAACAAGTT
ACTTCATTTGAAATTTGGCTGCACAAATTAGCTCACCATTTAATGGTGCTGGTAGCACCTCAGGAAAATCAAATCGACTG
AACTCTCCAGGCTTATTTGGCATTGCAAATATCATTGAATGCTCCTTCCACTGCAACCGATCATTAATTCCTAGTTCTCC
AAACAGGTTCTGCATATTTGGGTAASCCCCAACTTAAGGTGGATCAAGAATCAATGTCACAAGTTATTTGCTGCATCATA
ATGTATCTTAAAAAGAAACAAGAAGAATCATCTACATTCATGCTCATAAGAAATTCAAAAAAGCACGGAACAAGATAAGG
CAAATAATCGAATTCCCGCGGCCGCCATGGCGGCCGGGAGCATGCGACGTCGGGCCCAATTCGCCCTATAGTGAGTCGTA
TTACAATTCACTGGCCGTCGTTTTACAACGTCGTGACTGGGAAAACCCTGGCGTTACCCAACTTAATCGCCTTGCAGCAC
ATCCCCCTTTCGCCAGCTGGCGTAATAGCGAAGAGGCCCGCACCGATCGCCCTTCCMAACAGTTGCGCAGCCTGAATGGC
GAATGGACGCGCCCTGTAGCGGCGCATTAAGCGCGGCGGGTGTGGTGGTTACGCGCAGCGTGACCGCTACACTTGCCAGC
GCCCTAGCGCCCGCTCCTTTCGCTTTCTTCCCTTCCTTTCTCGCCACGTTCGCGGCTTCCCCGRTCAAGCTCTAAATCGG
GGGGCTCCCGTWAGGCTTCCGATTAGTGCTTTACCGACACCTCGAACCCCAAAAACTTGAATTAGGGTGGAATGGGCTAY
A
>G2.C4
TKKKWMSGTTWWWCCMATCTTGTGCCTCAACATAAGATTGTCCACCCAGAATTGCTGGCAAGAGTCCAATTGCASATTTG
ACTTTCTCTGGCCAAGTAAGCATGTCATTATTCTTCAAGATGGCCCATATTCCTGCAAGACCAATTTGAACACAATATCC
ACATAAAAACAAGTTACTTCATTTGAAATTTGGCTGCACAAATTAGCTCACCATTTAATGGTGCTGGTAGCACCTCAGGA
AAATCAAATCGACTGAACTCTCCAGGCTTATTTGGCATTGCAAATATCATTGAATGCTCCTTCCACTGCAACCGATCATT
AATTCCTAGTTCTCCAAACAGGTTCTGCATATTTGGGTAAGCCCCAACTTAAGGTGGATCAAGAATCAATGTCACAAGTT
ATTTGCTGCATCATAATGTATCTTAAAAAGAAACAAGAAGAATCATCTACATTCATGCTCATAAGAAATTCAAAAAAGCA
CGGAACAAGATAAGGCAAAAATCGAATTCCCGCGGCCGCCATGGCGGCCGGGAGCATGCGACGTCGGGCCCAATTCGCCC
TATAGTGAGTCGTATTACAATTCACTGGCCGTCGTTTTACAACGTCGTGACTGGGAAAACCCTGGCGTTACCCAACTTAA
TCGCCTTGCAGCACATCCCCCTTTCGCCAGCTGGCGTAATAGCGAAGAGGCCCGCACCGATCGCCCTTCCCAACAGTTGC
GCAGCCTGAATGGCGAATGGACGCGCCCTGTAGCGGCGCATTAAGCGCGGCGGGTGTGGTGGTTACGCGCAGCGTGACCG
CTACACTTGCCAGCGCCCTAGCGCCCGCTCCTTTCGCTTTCTTCCCTTCCTTTCTCGCCACGTTCGCCGGCTTTCCCGTC
AAGCTCTAAATCGGGGGGGCTCCCTTTAGGGATCGAATTTAGTGCTTTACGGCACCTCGACCCCAAAAAAAAMTTTGAAG
T
>G3.C1
TRGGTTWWWWCMATCTTGTGCCTCAACATAAGATTGTCCACCCAGAATTGCTGGCAAGAGTCCAATTGCAAATTTGACTT
TCTCTGGCCAAGTAAGCATGTCATTATTCTTCAAGATGGCCCATATTCCTGCAAGACCAATTTGAACACAATATCCACAT
AAAAACAAGTTACTTCATTTGAAATTTGGCTGCACAAATTAGCTCACCATTTAATGGTGCTGGTAGCACCTCAGGAAAAT
CAAATCGACTGAACTCTCCAGGCTTATTTGGCATTGCAAATATCATTGAATGCTCCTTCCACTGCAACCGATCATTAATT
CCTAGTTCTCCAAACAGGTTCTGCATATTTGGGTAAGCCCCAACTTAAGGTGGATCAAGAATCAATGTCACAAGTTATTT
GCTGCATCATAATGTATCTTAAAAAGAAACAAGAAGAATCATCTACATTCATGCTCATAAGAAATTCAAAAAAGCACGGA
ACAAGATAAGGCAAATAATCGAATTCCCGCGGCCGCCATGGCGGCCGGGAGCATGCGACGTCGGGCCCAATTCGCCCTAT
AGTGAGTCGTATTACAATTCACTGGCCGTCGTTTTACAACGTCGTGACTGGGAAAACCCTGGCGTTACCCAACTTAATCG
CCTTGCAGCACATCCCCCTTTCGCCAGCTGGCGTAATAGCGAAGAGGCCCGCACCGATCGCCCTTCCCAACAGTTGCGCA
GCCTGAATGGCGAATGGACGCGCCCTGTAGCGGCGCATTAAGCGCGGCGGGTGTGGTGGTTACGCGCAGCGTGACCGCTA
CACTTGCCAGCGCCCTAGCGCCCGCTCCTTTCGCTTTCTTCCCTTCCTTTCTCGCCACGTTCGCCGGGCTTTCCCGTCAA
GCTCTAATCGGGGGGCTCCCTTAGGGTTCGATTAGTGCCTTTACGGCACCCTCGAACCCCTAAAAAACCTTTKGGATT
>G3.C2
CTTYCYTTTGGTKGCCTCAACATAAGATTGTCCACCCAGAATTGCTGGCAAGAGTCCAATTGCAAATTTGACTTTCTCTG
GCCAAGTAAGCATGTCATTATTCTTCAAGATGGCCCATATTCCTGCAAGACCAATTTGAACACAATATCCACATAAAAAC
AAGTTACTTCATTTGAAATTTGGCTGCACAAATTAGCTCACCATTTAATGGTGCTGGTAGCACCTCAGGAAAATCAAATC
GACTGAACTCTCCAGGCTTATTTGGCATTGCAAATATCATTGAATGCTCCTTCCACTGCAACCGATCATTAATTCCTAGT
TCTCCAAACAGGTTCTGCATATTTGGGTAAGCCCCAACTTAAGGTGGATCAAGAATCAATGTCACAAGTTATTTGCTGCA
TCATAATGTATCTTAAAAAGAAACAAGAAGAATCATCTACATTCATGCTCATAAGAAATTCAAAAAAGCACGGAACAAGA

TAAGGCAAATAAATCGAATTCCCGCGGCCGCCATGGCGGCCGGGAGCATGCGACGTCGGGCCCAATTCGCCCTATAGTGA
GTCGTATTACAATTCACTGGCCGTCGTTTTACAACGTCGTGACTGGGAAAACCCTGGCGTTACCCAACTTAATCGCCTTG
CAGCACATCCCCCTTTCGCCAGCTGGCGTAATAGCGAAGAGGCCCGCACCGATCGCCCTTCCCAACAGTTGCGCAGCCTG
AATGGCGAATGGACGCGCCCTGTAGCGRCGCATTAAGCGCGGCGGGTGTGGTGGTTACGCGCAGCGTGACCGCTACACTT
GCCAGCGCCCTAGCGCCCGCTCCTTTCGCTTTCTTCCCTCCCTTTCTCGGCACGTTCSGCGGCTTTCCCCGTCAAGMTCT
AAATCGGGGGCCTCCCTTTAAGGGTTCCGATTAGAGCTTTACGGCACCTCGAACCCAAAAACGTTGATAGGGTATGGTCA
CGTAGAKGGGCTCTCAGC
>G3.C3
CTKRSCCCTYMAACMATAAARAAWTGTTCCACCCAGAATTGCTGGCAAGAGTCCAATTGCAMMTTTGACTTTCTCTGGCC
AAGTAAGCATGTCATTATTCTTCAMCSATGGCCCATATTCCTGCAAGACCAATTTGAACACAATATCCACATAAAAACAA
GTTACTTCATTTGAAATTTGGCTGCACAAATTAGCTCACCATTTAATGGTGCTGGTAGCACCTCAGGAAAATCAAATCGA
CTGAACTCTCCAGGCTTATTTGGCATTGCAAATATCATTGAATGCTCCTTCCACTGCAACCGATCATTAATTCCTAGTTC
TCCAAACAGGTTCTGCATATTTGGGTAASCCCCAACTTAAGGTGGATCAAGAATCAATGTCACAAGTTATTTGCTGCATC
ATAATGTATCTTAAAAAGAAACAAGAAGAATCATCTACATTCATGCTCATAAGAAATTCAAAAAAGCACGGAACAAGATA
AGGCAAATAATCGAATTCCCGCGGCCGCCATGGCGGCCGGRAGCATGCGACGTCGGGCCCAATTCGCCCTATAGTGAGTC
GTATTACAATTCACTGGCCGTCGTTTTACAACGTCGTGACTGGGAAAACCCTGGCGTTACCCAACTTAATCGCCTTGCAS
CACATCCCCCTTTCGCCAGCTGGCGTAATAGCSAAGAGGCCCGCACCGATCGCCCTTCCCAACAGTTGCSCASCCTGAAT
>G3.C4
GKKTWKYMCCMTYCCTTGGKTGGCCTCMACATAAGATTGTCCACCCAGAATTGCTGGCAAGAGTCCAATTGCAAATTTGA
CTTTCTCTGGCCAAGTAAGCATGTCATTATTCTTCAAGATGGCCCATATTCCTGCAAGACCAATTTGAACACAATATCCA
CATAAAAACAAGTTACTTCATTTGAAATTTGGCTGCACAAATTAGCTCACCATTTAATGGTGCTGGTAGCACCTCAGGAA
AATCAAATCGACTGAACTCTCCAGGCTTATTTGGCATTGCAAATATCATTGAATGCTCCTTCCACTGCAACCGATCATTA
ATTCCTAGTTCTCCAAACAGGTTCTGCATATTTGGGTAASCCCCAACTTAAGGTGGATCAAGAATCAATGTCACAAGTTA
TTTGCTGCATCATAATGTATCTTAAAAAGAAACAAGAAGAATCATCTACATTCATGCTCATAAGAAATTCAAAAAAGCAC
GGAACAAGATAAGGCAAAAATCGAATTCCCGCGGCCGCCATGGCGGCCGGGAGCATGCGACGTCGGGCCCAATTCGCCCT
ATAGTGAGTCGTATTACAATTCACTGGCCGTCGTTTTACAACGTCGTGACTGGGAAAACCCTGGCGTTACCCAACTTAAT
CGCCTTGCAGCACATCCCCCTTTCGCCAGCTGGCGTAATAGCGAAGAGGCCCGCACCGATCGCCCTTCCCAACAGTTGCG
CAGCCTGAATGGCGAATGGACGCGCCCTGTAGCGGCGCATTWARGCGCGGCGGGTGTGGTGGTTACGCGCAGCGTGACCG
CTACACTTGCCAGCGCCCTAGCGCCCGCTCCTTTCGCTTTCTTCCCTTCCTTTCTCGCCACGTTCGCCGGCTTTCCCGTC
AAGCTYTAATCGGGGGCTCCGTTTAGGTTCCGATTTAGTGCTTAACGGCACCTCGAACCCCAAAAAACCTGTGAATTWAA
GGGGGTSTG
>S1.C3
GCCAMMMMMCYCTTTTTGAMCTKTTCTCTGGCCAAGTAAGCATGTCATTAATTCTTCMMSYATGGCCCATATTCCTGCAA
GACCAATTTGAACACAATATCCACATAAAAACAAGTTACTTCATTTGAAATTTGGCTGCACAAATTAGCTCACCATTTAA
TGGTGCTGGTAGCACCTCAGGAAAATCAAATCGACTGAACTCTCCAGGCTTATTTGGCATTGCAAATATCATTGAATGCT
CCTTCCACTGGCAACCGATCATTAATTCCTAGTTCTCCAAACAGGTTCTGCATATTTGGGTAAGCCCCAACTTAAGGTGG
ATCAAGAATCAATGTCACAAGTTATTTGCTGCATCATAATGTATCTTAAAAAGAAACAAGAAGAATCATCTACATTCATG
CTCATAAGAAATTCAAAAAAGCACGGAACAAGATAAGGCAAAAATCGAATTCCCGCGGCCGCCATGGCGGCCGGGAGCAT
GCGACGTCSGGCCCAATTCGCCCTATAGTGAGTCGTATTACAATTCACTGGCCGTCGTTTTACAACGTCGTGACTGGRAA
AACCCTGGCGTTACCCAACTTAATCGCCTTGCAGCACATCCCCCTTTCGCCAGCTGGCGTAATAGCGAAGAGGCCCGCAC
CGATCSYCCTTCCCAAYAGTTGCGCAGCCTGAATGGCGAATGGACGCGCCTWGTAGCRGCGCATAAGCGCGRCGGGTGTG
GWGGTTACGCGCAGCGTGWCCGCTACACTTGCYRYGCCTAACGCCRCTCATTCRCTTACTCCCTCTTTMTYGCCMCGTTC
GACGGCATCCCGTCAAGCTYAAATCGGGGTTCCTTAGGATCGATATTGCCTTACCGGACCTYRACTCCAAAACTGATAGG
GTGATGSTMCG
>S1.C4
GTCCCTTTKGKTGGSCCTMAACATAAGGATTGTCCACCCAGAATTGCTGGCAAGAGTCCAATTGCAAATTTGACTTTCTC
TGGCCAAGTAAGCATGTCATTATTCTTCAAGATGGCCCATATTCCTGCAAGACCAATTTGAACACAATATCCACATAAAA
ACAAGTTACTTCATTTGAAATTTGGCTGCACAAATTAGCTCACCATTTAATGGTGCTGGTAGCACCTCAGGAAAATCAAA
TCGACTGAACTCTCCAGGCTTATTTGGCATTGCAAATATCATTGAATGCTCCTTCCACTGCAACCGATCATTAATTCCTA
GTTCTCCAAACAGGTTCTGCATATTTGGGTAAGCCCCAACTTAAGGTGGATCAAGAATCAATGTCACAAGTTATTTGCTG
CATCATAATGTATCTTAAAAAGAAACAAGAAGAATCATCTACATTCATGCTCATAAGAAATTCAAAAAAGCACGGAACAA
GATAAGGCAAATAATCGAATTCCCGCGGCCGCCATGGCGGCCGGGAGCATGCGACGTCGGGCCCAATTCGCCCTATAGTG
AGTCGTATTACAATTCACTGGCCGTCGTTTTACAACGTCGTGACTGGGAAAACCCTGGCGTTACCCAACTTAATCGCCTT
GCAGCACATCCCCCTTTCGCCAGCTGGCGTAATAGCGAAGAGGCCCGCACCGATCGCCCTTCCCAACAGTTGCGCAGCCT
GAATGGCGAATGGACGCGCCCTGTAGCGGCGCATTAAGCGCGGCGGGTGTGGTGGTTACGCGCAGCGTGACCGCTACACT
TGCCAGCGCCCTAGCGCCCGCTCCTTTCGCTTTCTTCCTTTCTTTCTCGCACGTTCGCCGGCTTTCCCCGTCAGCTCTAA
ATCGGGGGCTCCTTAGGGTCGAATTAGTGCCTTTACCGGCACCTCGACCCCTAAAACTTRGAATTAAGGGGKGRATA

>S2.C1
AYKKKKRGGTTWWCMTCTTGGTGCCTCAACATAAGATTGTCCACCCAGAATTGCTGGCAAGAGTCCAATTGCAAATTTGA
CTTTCTCTGGCCAAGTAAGCATGTCATTATTCTTCAAGATGGCCCATATTCCTGCAAGACCAATTTGAACACAATATCCA
CATAAAAACAAGTTACTTCATTTGAAATTTGGCTGCACAAATTAGCTCACCATTTAATGGTGCTGGTAGCACCTCAGGAA
AATCAAATCGACTGAACTCTCCAGGCTTATTTGGCATTGCAAATATCATTGAATGCTCCTTCCACTGCAACCGATCATTA
ATTCCTAGTTCTCCAAACAGGTTCTGCATATTTGGGTAAGCCCCAACTTAAGGTGGATCAAGAATCAATGTCACAAGTTA
TTTGCTGCATCATAATGTATCTTAAAAAGAAACAAGAAGAATCATCTACATTCATGCTCATAAGAAATTCAAAAAAGCAC
GGAACAAGATAAGGCAAATAATCGAATTCCCGCGGCCGCCATGGCGGCCGGGAGCATGCGACGTCGGGCCCAATTCGCCC
TATAGTGAGTCGTATTACAATTCACTGGCCGTCGTTTTACAACGTCGTGACTGGGAAAACCCTGGCGTTACCCAACTTAA
TCGCCTTGCAGCACATCCCCCTTTCGCCAGCTGGCGTAATAGCGAAGAGGCCCGCACCGATCGCCCTTCCCAACAGTTGC
GCAGCCTGAATGGCGAATGGACGCGCCCTGTAGCGGCGCATTAAGCGCGGCGGGTGTGGTGGTTACGCGCAGCGTGACCG
CTACACTTGCCAGCGCCCTAGCGCCCGCTCCTTTCGCTTTCTTCCCTTCCTTTCTCGCACGTTCGCCGGCTTTCCCGTCA
GCTCTAAATCGGGGGGCTCCCTTTAGGGATCGAATTAGTGCTTACGGCMACCTCGAACCCCAAAAAACTKGAATTAGGGG
KKGAATW
>S2.C2
GKKWKYRRGTTWWWCCMTCCTTTGTGGCCTCAACATAAGATTGTCCACCCAGAATTGCTGGCAAGAGTCCAATTGCAAAT
TTGACTTTCTCTGGCCAAGTAAGCATGTCATTATTCTTCAAGATGGCCCATATTCCTGCAAGACCAATTTGAACACAATA
TCCACATAAAAACAAGTTACTTCATTTGAAATTTGGCTGCACAAATTAGCTCACCATTTAATGGTGCTGGTAGCACCTCA
GGAAAATCAAATCGACTGAACTCTCCAGGCTTATTTGGCATTGCAAATATCATTGAATGCTCCTTCCACTGCAACCGATC
ATTAATTCCTAGTTCTCCAAACAGGTTCTGCATATTTGGGTAAGCCCCAACTTAAGGTGGATCAAGAATCAATGTCACAA
GTTATTTGCTGCATCATAATGTATCTTAAAAAGAAACAAGAAGAATCATCTACATTCATGCTCATAAGAAATTCAAAAAA
GCACGGAACAAGATAAGGCAAATAATCGAATTCCCGCGGCCGCCATGGCGGCCGGGAGCATGCGACGTCGGGCCCAATTC
GCCCTATAGTGAGTCGTATTACAATTCACTGGCCGTCGTTTTACAACGTCGTGACTGGGAAAACCCTGGCGTTACCCAAC
TTAATCGCCTTGCAGCACATCCCCCTTTCGCCAGCTGGCGTAATAGCGAAGAGGCCCGCACCGATCGCCCTTCCCAACAG
TTGCGCAGCCTGAATGGCGAATGGACGCGCCCTGTAGCGGCGCATTAAGCGCGGCGGGTGTGGTGGTTACGCGCAGCGTG
ACCGCTACACTTGCCAGCGCCCTAGCGCCCGCTCCTTTCGCTTTCTTCCTTCCTTTCTCGCACGTTCGCCGGGCTTTCCC
CGTCAGCTCTAAATCGGGGGGCTCCCTTTAGGTTCCGAATTAGTGCTTACGGCACCCTCGACCCCCCAAAAAAAMCTTTG
GAKTTTG
>S2.C3
TCMWWKGGTGGCCYTCMWCATAAGATTGTCCACCCAGAATTGCTGGCAAGAGTCCAATTGCAAATTTGACTTTCTCTGGC
CAAGTAAGCATGTCATTATTCTTCAAGATGGCCCATATTCCTGCAAGACCAATTTGAACACAATATCCACATAAAAACAA
GTTACTTCATTTGAAATTTGGCTGCACAAATTAGCTCACCATTTAATGGTGCTGGTAGCACCTCAGGAAAATCAAATCGA
CTGAACTCTCCAGGCTTATTTGGCATTGCAAATATCATTGAATGCTCCTTCCACTGCAACCGATCATTAATTCCTAGTTC
TCCAAACAGGTTCTGCATATTTGGGTAAGCCCCAACTTAAGGTGGATCAAGAATCAATGTCACAAGTTATTTGCTGCATC
ATAATGTATCTTAAAAAGAAACAAGAAGAATCATCTACATTCATGCTCATAAGAAATTCAAAAAAGCACGGAACAAGATA
AGGCAAATAATCGAATTCCCGCGGCCGCCATGGCGGCCGGGAGCATGCGACGTCGGGCCCAATTCGCCCTATAGTGAGTC
GTATTACAATTCACTGGCCGTCGTTTTACAACGTCGTGACTGGGAAAACCCTGGCGTTACCCAACTTAATCGCCTTGCAG
CACATCCCCCTTTCGCCAGCTGGCGTAATAGCGAAGAGGCCCGCACCGATCGCCCTTCCCAACAGTTGCGCAGCCTGAAT
GGCGAATGGACGCGCCCTGTAGCGGCGCATTAAGCGCGGCGGGTGTGGTGGTTACGCGCAGCGTGACCGCTACACTTGCC
AGCGCCCTAGCGCCCGCTCCTTTCGCTTTCTTCCCTTCCTTTCTCGCACGTTCGCCGGCTTTCCCGTCAAGCTCTAAATC
GGGGGGCTCCCTTTAGGTTCCGATTAGTGCCTTACGGCACTCGAACCCCAAAACTTGATAAGTGAATGGTCACGTAGTGG
GACCAATCKCACCCCGCT
>S2.C4
CMACACAACCCTTATTGAACTTTGCTCCTGGCCAAGTAAGCATGTCATTATTCTTCMCCSATWGGCCCATATTCCTGCAA
GACCAATTTGAACACAATATCCACATAAAAACAAGTTACTTCATTTGAAATTTGGCTGCMCAAATTAGCTCACCATTTAA
TGGTGCTGGTAGCACCTCAGGAAAATCAAATCGACTGAACTCTCCAGGCTTATTTGGCATTGCAAATATCATTGAATGCT
CCTTCCACTGCAACCGATCATTAATTCCTAGTTCTCCAAACAGGTTCTGCATATTTGGGTAAGCCCCAACTTAAGGTGGA
TCAAGAATCAATGTCACAAGTTATTTGCTGCATCATAATGTATCTTAAAAAGAAACAAGAAGAATCATCTACATTCATGC
TCATAAGAAATTCAAAAAAGCACGGAACAAGATAAGGMAAAAATCGAATTCCCGCGGCCGCCATGGCGGCCGGGAGCATG
CGACGTCGGGCCCAATTCRCCCTATAGTGAGTCGYATTACAATTCACTGGCCGTCGTTTTACAACGTCGTGACTGGGAAA
ACCCTGGCGTTACCCAACTTAATCGCCTTGCAGCACATCCCCCTTTCGCCAGCTGGCGTAATAGCGAAGAGGCCCGCACC
GATCGCCCTTCCCAACAGTTGCGCAGCCTGAATGGCGAATGGACGCGCCCTGTAGCGGCGCATTAAGCGCGGCGGGTGTG
GKGGTTACGCGCAGCRTGACCRCTWCAMTTGCCAGCGCCCTAGCGCMCGCTCCTTCGCTTYCTCCCTTCCTTCWCGCACG
TTCGCCGGCTTTCCCCRTCAGCTCWAATCGGGGGCTCCTTTWARGGATTCGATTAATGCTACGCACCYCSACCCCAAAAA
ACTGRATTAARGGKWATGTTACACGWAGKGGGCCM
>S3.C1
CGTWTWCCMMTTCCTTTGTGCCTCAACATAAGATTGTCCACCCAGAATTGCTGGCAAGAGTCCAATTGCAAATTTGACTT

TCTCTGGCCAAGTAAGCATGTCATTATTCTTCAAGATGGCCCATATTCCTGCAAGACCAATTTGAACACAATATCCACAT
AAAAACAAGTTACTTCATTTGAAATTTGGCTGCACAAATTAGCTCACCATTTAATGGTGCTGGTAGCACCTCAGGAAAAT
CAAATCGACTGAACTCTCCAGGCTTATTTGGCATTGCAAATATCATTGAATGCTCCTTCCACTGCAACCGATCATTAATT
CCTAGTTCTCCAAACAGGTTCTGCATATTTGGGTAAGCCCCAACTTAAGGTGGATCAAGAATCAATGTCACAAGTTATTT
GCTGCATCATAATGTATCTTAAAAAGAAACAAGAAGAATCATCTACATTCATGCTCATAAGAAATTCAAAAAAGCACGGA
ACAAGATAAGGCAAATAATCGAATTCCCGCGGCCGCCATGGCGGCCGGGAGCATGCGACGTCGGGCCCAATTCGCCCTAT
AGTGAGTCGTATTACAATTCACTGGCCGTCGTTTTACAACGTCGTGACTGGGAAAACCCTGGCGTTACCCAACTTAATCG
CCTTGCAGCACATCCCCCTTTCGCCAGCTGGCGTAATAGCGAAGAGGCCCGCACCGATCGCCCTTCCCAACAGTTGCGCA
GCCTGAATGGCGAATGGACGCGCCCTGTAGCGGCGCATTAAGCGCGGCGGGTGTGGTGGTTACGCGCAGCGTGACCGCTA
CACTTGCCAGCGCCCTAGCGCCCGCTCCTTTCGCTTTCTTCCCTTCCTTTCTCGCCACGTTCGCCSGGCTTTCCCCGTCA
AGCTCTAATCGGGGGGCTCCCTTTAAGGATCGATTTAGTGCTTTACGGCACCCTYCGACCCAAAAACTGGAATTAGGKGA
TGGTTCCACGTATACTGTGCCCTCACYACT
>S3.C3
GAAGKTTCCCMAATTTTGGCAAATTTTGACTTTCTCTGGCCAAGTAAGCATGTCATTATTCTTCAAGATGGCCCATATTC
CTGCAAGACCAATTTGAACACAATATCCACATAAAAACAAGTTACTTCATTTGAAATTTGGCTGCACAAATTAGCTCACC
ATTTAATGGTGCTGGTAGCACCTCAGGAAAATCAAATCGACTGAACTCTCCAGGCTTATTTGGCATTGCAAATATCATTG
AATGCTCCTTCCACTGCAACCGATCATTAATTCCTAGTTCTCCAAACAGGTTCTGCATATTTGGGTAAGCCCCAACTTAA
GGTGGATCAAGAATCAATGTCACAAGTTATTTGCTGCATCATAATGTATCTTAAAAAGAAACAAGAAGAATCATCTACAT
TCATGCTCATAAGAAATTCAAAAAAGCACGGAACAAGATAAGGCAAATAATCGAATTCCCGCGGCCGCCATGGCGGCCGG
GAGCATGCGACGTCGGGCCCAATTCGCCCTATAGTGAGTCGTATTACAATTCACTGGCCGTCGTTTTACAACGTCRTGAC
TGGRAAAACCCTGGCGTTACCCAACTTAATCGCCTTGCAGCACATCCCCCTTTCGCCAGCTGGCGTAATAGCGAAGAGGC
CCCGCACCGATCGCCCTTTCCCAACAGTTGCGCAGCCTGAATGGCGAATGGACGCGCCCTGTAGCGGCGCATTAAGCGCG
GCGGGTGTGGTGGTTACGCGCAGCGTGACCGGCTACACTTGCARCGCCCTAGCGCCCGCTCCTTTCGCTTTCTCCCTTCC
TTTMTCGCMMSTTCRCCGGCTTTCCCGTCAAGCATCWAATCGGGGGCCTCCTTWRRGGTCCGATTWGKSCTTACGGCMCC
TYCGACMCCMAAAACTGGATGAGGGKGAKTGATCACGTYGATGRGCATCGCCCKRGATARMCGGGTTCTTCGCCCCTMKG
ARCGT
>S4.C1
GKKKKRRGKTTTWYCMTTCTTTGGTGCCCTCAACATAARGATTGTCCACCCAGAATTGCTGGCAAGAGTCCAATTGCAAA
TTTGACTTTCTCTGGCCAAGTAAGCATGTCATTATTCTTCAAGATGGCCCATATTCCTGCAAGACCAATTTGAACACAAT
ATCCACATAAAAACAAGTTACTTCATTTGAAATTTGGCTGCACAAATTAGCTCACCATTTAATGGTGCTGGTAGCACCTC
AGGAAAATCAAATCGACTGAACTCTCCAGGCTTATTTGGCATTGCAAATATCATTGAATGCTCCTTCCACTGGCAACCGA
TCATTAATTCCTAGTTCTCCAAACAGGTTCTGCATATTTGGGTAAGCCCCAACTTAAGGTGGATCAAGAATCAATGTCAC
AAGTTATTTGCTGCATCATAATGTATCTTAAAAAGAAACAAGAAGAATCATCTACATTCATGCTCATAAGAAATTCAAAA
AAGCACGGAACAAGATAAGGCAAATAATCGAATTCCCGCGGCCGCCATGGCGGCCGGGAGCATGCGACGTCGGGCCCAAT
TCGCCCTATAGTGAGTCGTATTACAATTCACTGGCCGTCGTTTTACAACGTCGTGACTGGGAAAACCCTGGCGTTACCCA
ACTTAATCGCCTTGCAGCACATCCCCCTTTCGCCAGCTGGCGTAATAGCGAAGAGGCCCGCACCGATCGCCCTTCCCAAC
AGTTGCGCAGCCTGAATGGCGAATGGACGCGCCCTGTAGCGGCGCATTAAGCGCGGCGGGTGTGGTGGTTACGCGCAGCG
TGACCGCTACACTTGCCAGCGCCCTAGCGCCCGCTCCTTTCGCTTTCTTCCCTTCCTTTCTCGCACGTTCGCCGGCTTTC
CCGTCAAGCTCTAATCGGGGGGCTCCCTTTAGGATCGAATTAGTGCTTACGCMCCTCGACCCCCAAAAACTTGAATTRAG
GGGGTKGATG
>S4.C3
GWTKWWRGTTWWMMMATCTKGGTGCCTMAACATAAGATTGTCCACCCAGAATTGCTGGCAAGAGTCCAATTGCAAATTTG
ACTTTCTCTGGCCAAGTAAGCATGTCATTATTCTTCAAGATGGCCCATATTCCTGCAAGACCAATTTGAACACAATATCC
ACATAAAAACAAGTTACTTCATTTGAAATTTGGCTGCACAAATTAGCTCACCATTTAATGGTGCTGGTAGCACCTCAGGA
AAATCAAATCGACTGAACTCTCCAGGCTTATTTGGCATTGCAAATATCATTGAATGCTCCTTCCACTGSMAACCRATMAT
TAWTTCYTAKTTCTCCAAAMRGGTTCTGCATATTTGGGTAACCCCCAACTTARGGGGAATCAAAAATCAWGGYMMCAAGT
TATTTGCTGCWTCATAATGWATCTAAAAAAAAAACAAAAAAAATCATCTACWTTCWTGCTCATAAAAAATTCAAAAAASC
ASGAAACAAAATAAGGMAAATAAYCRAATTCCCSCGGCCGCCWTGGSGGCCGGAASCATGCRACKYCGGGCCCAATTCSC
CCTATAKGGAGTCKAATTACAATTMACGGGCCGTCKTTTTACAACKYCKGGACTGGAAAAACCYTGSSKTTACCCAMCTW
AWTCGCYTKGMASMACATCCCCCTTTCGCMASCTGSCKWAWTAGCRAAAAGGCCSGMACCRATCGCCCTTCCMAACAGTT
GCGMAGCCTGAWGGCGAATGAACGCGCCCTGWAGCGGCGMATTAAGCGCGCGGTGTGTGTTAMSCGCAGCGTGAACGCTA
CACTGCAGSCCTACGCCGCTCTTTCGCTCTCATCTYAGCMGTCGCGCTCCGTCAGYTAATCGGCTCGTAGATCGATAKCT
ACGACCTGACCAAAACTGAATA
>S4.C4
ASCMMMMMMMYYYTTWWGAAMTTTCTCTGGCCAAGTAAGCATGTCATTATTCTTCAMSYATGGCCCATATTCCTGCAAGA
CCAATTTGAACACAATATCCACATAAAAACAAGTTACTTCATTTGAAATTTGGCTGCACAAATTAGCTCACCATTTAATG
GTGCTGGTAGCACCTCAGGAAAATCAAATCGACTGAACTCTCCAGGCTTATTTGGCATTGCAAATATCATTGAATGCTCC

TTCCACTGCAACCGATCATTAATTCCTAGTTCTCCAAACAGGTTCTGCATATTTGGGTAAGCCCCAACTTAAGGTGGATC
AAGAATCAATGTCACAAGTTATTTGCTGCATCATAATGTATCTTAAAAAGAAACAAGAAGAATCATCTACATTCATGCTC
ATAAGAAATTCAAAAAAGCACGGAACAAGATAAGGCAAATAATCGAATTCCCGCGGCCGCCATGGCGGCCGGGAGCATGC
GACGTCGGGCCCAATTCGCCCTATAGTGAGTCGTATTACAATTCACTGGCCGTCGTTTTACAACGTCGTGACTGGGAAAA
CCCTGGCGTTACCCAACTTAATCGCCTTGCAGCACATCCCCCTTTCGCCAGCTGGCGTAATAGCGAAGAGGCCCGCACCG
ATCGCCCTTCCCAACAGTTGCGCAGCCTGAATGGCGAATGGACGCGCCCTGTAGCGGCGCATTAAGCGCGGCGGGTGTGG
TGGTTACGCGCAGCGTGACCGCTACACTTGCCAGCGCCCTAGCGCCCGCTCTTTCGCTTTCTTCCCTTCTTCTCGCCACG
TTCGCCGGCTTCCCGTCAGCTCTAAATCGGGGGCTCCCTTAGGTTCGATTARTGCTTTACGGCAMCTCGAACCCAAAACT
GATAGGGTGAWYGTCMGGTAGTGGGCCATCGCCTGAWGACGTTTCGCTTGACGTTGARTCMGTCTCTTAAKATG
>S3.C5
CYKKKYSSRGGTWWWWMCMCTCTTGTGCCTCAACATAAGATTGTCCACCCAGAATTGCTGGCAAGAGTCCAATTGCAAAT
TTGACTTTCTCTGGCCAAGTAAGCATGTCATTATTCTTCAAGATGGCCCATATTCCTGCAAGACCAATTTGAACACAATA
TCCACATAAAAACAAGTTACTTCATTTGAAATTTGGCTGCACAAATTAGCTCACCATTTAATGGTGCTGGTAGCACCTCA
GGAAAATCAAATCGACTGAACTCTCCAGGCTTATTTGGCATTGCAAATATCATTGAATGCTCCTTCCACTGCAACCGATC
ATTAATTCCTAGTTCTCCAAACAGGTTCTGCATATTTGGGTAAGCCCCAACTTAAGGTGGATCAAGAATCAATGTCACAA
GTTATTTGCTGCATCATAATGTATCTTAAAAAGAAACAAGAAGAATCATCTACATTCATGCTCATAAGAAATTCAAAAAA
GCACGGAACAAGATAAGGCAAATAATCGAATTCCCGCGGCCGCCATGGCGGCCGGGAGCATGCGACGTCGGGCCCAATTC
GCCCTATAGTGAGTCGTATTACAATTCACTGGCCGTCGTTTTACAACGTCGTGACTGGGAAAACCCTGGCGTTACCCAAC
TTAATCGCCTTGCAGCACATCCCCCTTTCGCCAGCTGGCGTAATAGCGAAGAGGCCCGCACCGATCGCCCTTCCCAACAG
TTGCGCAGCCTGAATGGCGAATGGACGCGCCCTGTAGCGGCGCATTAAGCGCGGCGGGTGTGGTGGTTACGCGCAGCGTG
ACCGCTACACTTGCCAGCGCCCTAGCGCCCGCTCCTTCGCTTTCTTCCCTTCCTTTCTCGCCACGTTCGCCGGCTTCCCG
TCAAGCTCTAAATCGGGGGGGCTCCCTTTAGGGTTCGATTAGTGCTTTACGCACTCGACCAAACCTTGAATTAAGGGCGT
GAAKWGGCTGATA
>S3.C6
GRMMRYMMTAAMACTACRACCTKCRTWWMMTCTTGTGCCTCACATAAGATTGTCCACCCRGAATTGCTGGCAAGAGTCCA
ATTGCAAATTTGACTTTCTCTGGCCAAGTAAGCATGTCATTATTCTTCAAGATGGCCCATATTCCTGCAAGACCAATTTG
AACACAATATCCACATAAAAACAAGTTACTTCATTTGAAATTTGGCTGCACAAATTAGCTCACCATTTAATGGTGCTGGT
AGCACCTCAGGAAAATCAAATCGACTGAACTCTCCAGGCTTATTTGGCATTGCAAATATCATTGAATGCTCCTTCCACTG
CAACCGATCATTAATTCCTAGTTCTCCAAACAGGTTCTGCATATTTGGGTAAGCCCCAACTTAAGGTGGATCAAGAATCA
ATGTCACAAGTTATTTGCTGCATCATAATGTATCTTAAAAAGAAACAAGAAGAATCATCTACATTCATGCTCATAAGAAA
TTCAAAAAAGCACGGAACAAGATAAGGCAAATAATCGAATTCCCGCGGCCGCCATGGCGGCCGGGAGCATGCGACGTCGG
GCCCAATTCGCCCTATAGTGAGTCGTATTACAATTCACTGGCCGTCGTTTTACAACGTCGTGACTGGGAAAACCCTGGCG
TTACCCAACTTAATCGCCTTGCAGCACATCCCCCTTTCGCCAGCTGGCGTAATAGCGAAGAGGCCCGCACCGATCGCCCT
TCCCAACAGTTGCGCAGCCTGAATGGCGAATGGACGCGCCCTGTAGCGGCGCATTAAGCGCGGCGGGTGTGGTGGTTACG
CGCAGCGTGACCGCTACACTTGCCAGCGCCCTAGCGCCCGCTCCTTTCGCTTTCTTCCCTTCCTTTCTCGCCACGTTCGC
CGGCTTTCCCCGTCAAGCTCTAATCGGGGGGCTCCCTTTTAGGATCCGAATATGCCTTACGCACTCGGACCCCCCAGAGC
AA
>S4.C5
GKKWMRGWWWCCMTCTTGTGCCTCAACATAAGATTGTCCACCCAGAATTGCTGGCAAGAGTCCAATTGCAAATTTGACTT
TCTCTGGCCAAGTAAGCATGTCATTATTCTTCAAGATGGCCCATATTCCTGCAAGACCAATTTGAACACAATATCCACAT
AAAAACAAGTTACTTCATTTGAAATTTGGCTGCACAAATTAGCTCACCATTTAATGGTGCTGGTAGCATCTCAGGAAAAT
CAAATCGACTGAACTCTCCAGGCTTATTTGGCATTGCAAATATCATTGAATGCTCCTTCCACTGGCAACCGATCATTAAT
TCCTAGTTCTCCAAACAGGTTCTGCATATTTGGGTAAGCCCCAACTTAAGGTGGATCAAGAATCAATGTCACAAGTTATT
TGCTGCATCATAATGTATCTTAAAAAGAAACAAGAAGAATCATCTACATTCATGCTCATAAGAAATTCAAAAAAGCACGG
AACAAGATAAGGCAAATAATCGAATTCCCGCGGCCGCCATGGCGGCCGGGAGCATGCGACGTCGGGCCCAATTCGCCCTA
TAGTGAGTCGTATTACAATTCACTGGCCGTCGTTTTACAACGTCGTGACTGGGAAAACCCTGGCGTTACCCAACTTAATC
GCCTTGCAGCACATCCCCCTTTCGCCAGCTGGCGTAATAGCGAAGAGGCCCGCACCGATCGCCCTTCCCAACAGTTGCGC
AGCCTGAATGGCGAATGGACGCGCCCTGTAGCGGCGCATTAAGCGCGGCGGGTGTGGTGGTTACGCGCAGCGTGACCGCT
ACACTTGCCAGCGCCCTAGCGCCCGCTCCTTTCGCTTTCTTCCTTTCCTTTCTCGCCACGTTCGCCGGCTTCCCGTCAAG
CTCTAATCGGGGGCTCCGTTAGGATTCGAATTAGTGCTTTACGCCACCCTCGCACCCCAAAAAACYTTKGAACWG
>S4.C6
CRMMMAMSMMMKMMKMMAMMCCMSCARAYKKCGTWTMCMTCTTGTGCCTCACATAAGATTGTCCACCCAGAATTGCTGG
C
AAGAGTCCAATTGCAAATTTGACTTTCTCTGGCCAAGTAAGCATGTCATTATTCTTCAAGATGGCCCATATTCCTGCAAG
ACCAATTTGAACACAATATCCACATAAAAACAAGTTACTTCATTTGAAATTTGGCTGCACAAATTAGCTCACCATTTAAT
GGTGCTGGTAGCACCTCAGGAAAATCAAATCGACTGAACTCTCCAGGCTTATTTGGCATTGCAAATATCATTGAATGCTC
CTTCCACTGGMAACCGATMWTTAWTTCCTAKTTCTCCAAACRGGTTCTGCWTATTKGGGWAASCCCCAMCTWARGGGGGA

TCARRAATMAWGGTCMCARGTTATTTGCTGCATCATAATGTATCTAAAAAAAAAACAAAAAAAAATCWTCTACATTCWTGC
TCATAAAAAAATTCAAAAAASCMCGGAACAAAATAAGGMAAATAAYCRAATTCCCSCGGCCGCCAGGGSGGCCGGAASCAT
GCAACKYCGGGCCCAATTCSCCCTATAKKGAGTCKAATTACAATTCACGGGCCGYCKTTTTACAACGYCKKGACTGGAAA
AACCCTGSSKTTACCCAACTTAATCSCCTTGMASMACATCCCCCTTTCGCCAGYTGGSKWAAWASCAAAAAGGCCCSMAC
CGWTCGCCCTTCCMAACAGTKSCGCAGCCKGAAKGGCRAAKGRACGCGCCYTGWASCGSCSMATTARSSGCGCCGGKTGT
GGTGGTWMCGCGCAGSCGTGACCGYWAMCACTTGGCCARSCGCCCTAAGCGCCCGCYTCTTTCCGCTTTYTTCCCTTTCC
TTTCTCGCCACGTTCGCCGGGCTTTCCCGGTCAAGCTCTAGATCCGGGACTCTTTAAGGTCCCGAATTTAGKGCTTTAAC
SGSGCAC
